# Recent advances in bifunctional synthesis gas conversion to chemicals and fuels with a comparison to monofunctional processes†

**DOI:** 10.1039/d4cy00437j

**Published:** 2024-07-15

**Authors:** J. L. Weber, C. Hernández Mejía, K. P. de Jong, P. E. de Jongh

**Affiliations:** a Materials Chemistry and Catalysis, Universiteit Utrecht Universiteitsweg 99 Utrecht Netherlands p.e.dejongh@uu.nl

## Abstract

In order to meet the climate goals of the Paris Agreement and limit the potentially catastrophic consequences of climate change, we must move away from the use of fossil feedstocks for the production of chemicals and fuels. The conversion of synthesis gas (a mixture of hydrogen, carbon monoxide and/or carbon dioxide) can contribute to this. Several reactions allow to convert synthesis gas to oxygenates (such as methanol), olefins or waxes. In a consecutive step, these products can be further converted into chemicals, such as dimethyl ether, short olefins, or aromatics. Alternatively, fuels like gasoline, diesel, or kerosene can be produced. These two different steps can be combined using bifunctional catalysis for direct conversion of synthesis gas to chemicals and fuels. The synergistic effects of combining two different catalysts are discussed in terms of activity and selectivity and compared to processes based on consecutive reaction with single conversion steps. We found that bifunctional catalysis can be a strong tool for the highly selective production of dimethyl ether and gasoline with high octane numbers. In terms of selectivity bifunctional catalysis for short olefins or aromatics struggles to compete with processes consisting of single catalytic conversion steps.

## Abstract 2

The direct conversion of synthesis gas to chemicals and fuels using bifunctional catalysis has been reviewed. Some novel bifunctional pathways show promising advantages over the classic approach utilizing several reactor in series.

## Graphical abstract

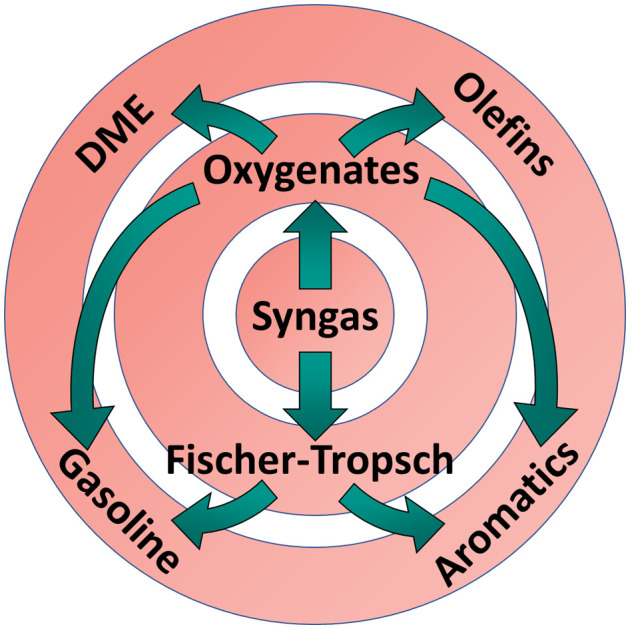

## 1. Introduction

Increasing worldwide demand for chemicals and transportation fuels, combined with the urgent need to move to more sustainable production processes, has spurred research towards alternatives to the traditional crude oil-based processes. Implementation is driven by geopolitical, economic, and environmental considerations. Processes such as gas-to-liquids (GTL) and coal-to-liquids (CTL) have been developed as a result of these considerations in the course of the 20th century with more recent stimuli being *i.e.*, the shale gas revolution in the USA, and the demand for transportation fuels and chemicals in China.^1–3^ GTL and CTL plants produce ultra-clean fuels and the possibility to shift to more sustainable feedstocks such as biomass or CO_2_ combined with renewable hydrogen.^4–6^

**Fig. 1:**
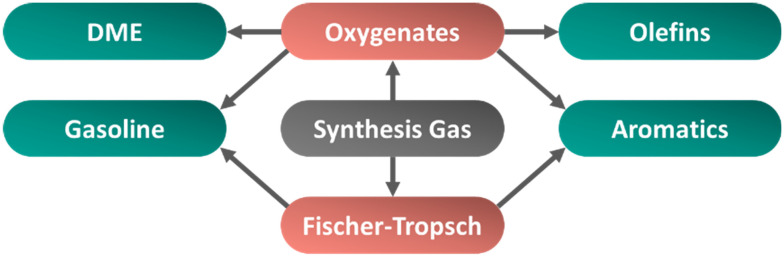
Schematic overview of pathways to convert synthesis gas (center) to dimethyl ether (DME), olefins, aromatics, or gasoline *via* oxygenate or Fischer–Tropsch intermediates.

Another advantage is the variety of products that can be selectively obtained from synthesis gas (Fig. 1), potentially playing a pivotal role in future chemical and energy industries. Synthesis gas can be directly or indirectly transformed to alcohols, long-chain hydrocarbons, olefins and aromatics, which constitute a sizable portion of industrial bulk chemicals and precursors for ultra-clean synthetic fuels.^7–9^ Currently, these transformations are performed in industry by thermally catalyzed processes (although other approaches such as electrochemical or plasma driven processes are being examined^10,11^) and largely rely on solid catalysts.^7,12^ A catalyst, typically a late-transition metal or metal carbide, that can hydrogenate molecules (a “hydrogenation function”) can help to selectively produce chemicals such as alcohols, olefins or paraffins. Addition of a second catalyst can be employed to couple reactions and expand the diversity of products to ethers, aromatics or branched hydrocarbons.^9,13^ Synergy between the functionalities in a catalyst mixture is particularly important for the desired performance, and is feasible by selecting the appropriate chemical properties and an optimal degree of intimacy.^14–17^ However, achieving the ideal composition while avoiding negative interference remains a challenge in these multifunctional catalytic systems.

In this review, we highlight the developments over the past ten years in bifunctional catalysis systems for the transformation of synthesis gas. In particular, we compare in detail bifunctional catalysis approaches to processes comprising two or more reactors with individual conversion steps. We start with general considerations from an academic and fundamental point of view, followed by a discussion of the relevance of bifunctionally-catalyzed processes of the most important industrial fuels and chemicals. Dimethyl ether (DME), light olefins, aromatics, and liquid fuels (gasoline, kerosene, and diesel) were selected based on their high demand and maturity of their production process. For each of these product classes background information is given, followed by discussing the recent developments concerning the catalysts for their direct production from synthesis gas and the associated challenges. The advantages and drawbacks are highlighted, considering activity, selectivity, and stability, but also taking the resulting product quality into consideration. An overview and critical analysis of yield and conversion of the latest reported data is discussed in each section, contributing to a more quantitative comparison. Finally, we summarize the key points and give a perspective for the utilization of bifunctional systems.

### 1.1. Bifunctional catalysis

The process conditions and type of catalyst determine the products derived from synthesis gas. The initial products obtained after direct carbon monoxide hydrogenation (referred here as Primary conversion processes, section 1.4) vary according to the degree of hydrogen addition and carbon–carbon coupling. A strong hydrogenation catalyst such as nickel can yield methane, the smallest of hydrocarbons. This is interesting for the hydrogenation of captured carbon dioxide with hydrogen which is produced by electrolysis using renewable power to provide sustainable fuels.^18–21^ A hydrogenation catalyst like iron or cobalt that removes the oxygen to form water and enables carbon–carbon coupling, leads to the formation of long-chain hydrocarbons. These hydrocarbons can be further processed to fuel-range compounds such as gasoline, kerosene, or diesel (Fischer–Tropsch route Fig. 1), or to olefins. If the carbon–oxygen bond is maintained, for instance using a copper-based catalyst, this leads to methanol, or with polymerization to long-chain oxygenates (oxygenates route Fig. 1). Addition of a second functionality (typically an acid site) during reaction can further transform these initial products or intermediates. These subsequent reactions (referred here as “Secondary conversion processes”, section 1.5) can lead to ethers, olefines, carboxylic acids, aromatics, or branched hydrocarbons. A single catalyst combining these functionalities is referred to as a bifunctional catalyst.

Applying a bifunctional catalyst or two different catalysts in a single reactor might reduce investment costs, energy requirements and complexity in comparison to two sequential reactors with individual monofunctional catalysts.^22^ Additionally, the combination of primary and secondary conversion catalysts can boost the overall synthesis gas conversion if the primary products are removed from this equilibrium effectively by the secondary conversion step. The combination of two catalytic functions in a single reactor can, however, also pose challenges. Undesired side reactions might emerge, for instance, the target products can further react on the primary catalytic function, or the feed might directly react on the secondary catalytic function. Examples of these side reactions are discussed in section 1.6, side reactions. The two catalytic functions can also negatively influence each other by electronic effects or migration of mobile species from one to the other. Another challenge is to find common reaction conditions in terms of reaction temperature, pressure, reaction atmosphere or space velocity for the two different catalysts. These challenges will be discussed more in detail throughout sections 2 to 5.

### 1.2. Relevant products

**Fig. 2:**
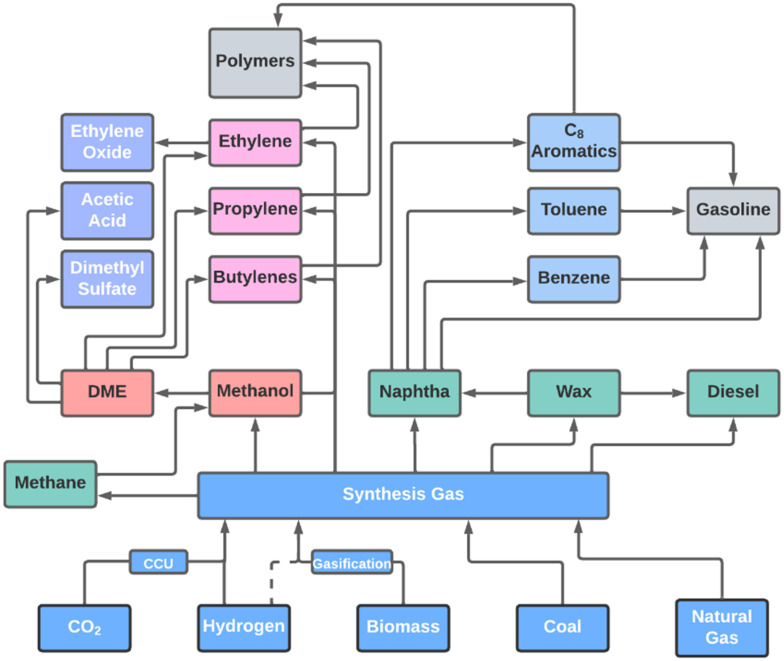
Overview of possible production pathways involving synthesis gas production, primary and secondary synthesis gas conversion processes. This scheme consists of synthesis gas conversion and the production of synthesis gas (light blue), the primary hydrocarbon products (methane, naphtha, wax, and diesel; given in green), oxygenates (red), short olefins as primary and secondary products (pink), other hydrocarbon secondary products (dark blue), examples of final product groups (grey), and final chemicals (purple).

One of the potential products of bifunctional synthesis gas conversion is dimethyl ether (DME), which has a total annual production capacity of 10 million tons per year and a wide variety of applications (Fig. 2).^23,24^ More recently, DME is increasingly used to substitute liquefied petroleum gas, or as blend in a fuel mixture. The attractiveness of DME for use as a fuel lies in its excellent ignition and combustion properties (cetane number = 55–60), and ease of storage and handling as a liquid under a pressure of only 5–6 bar. Another advantage is that no soot is formed upon combustion. Major efforts are underway mostly in Asia and North America to further develop the infrastructure and broad introduction of DME as a clean transportation fuel.^25^

Light olefins, namely ethylene, propylene, and butylenes, are fundamental building blocks for the chemical industry.^22^ More than 50% of ethylene and 60% of propylene produced worldwide is used for fabrication of polyolefins (Fig. 2). Butadiene and 1-butene are used in the production of polymers and rubbers and as precursor of various chemicals.^26^ Light olefins are currently made from fossil resources, have a high energy demand and associated emission of pollutants.^27,28^ Several renewable alternatives to produce light olefines have been proposed.^22,28^ Also, the increase in C_1_ and C_2_ feedstocks derived from shale gas has promoted alternative pathways for olefins production.^29^

Aromatics like benzene, toluene, xylenes, and ethylbenzene are important precursors for intermediates and polymers (Fig. 2).^26,30,31^ The good anti-knocking properties of some aromatic compounds also makes them a good octane-enhancer for gasoline.^32^ The use as anti-knocking agent depends on availability and price, for instance toluene is blended in regularly, while this is less often the case for xylene, as the latter has a higher value for other chemical applications.^26,33^

Liquid transportation fuels (diesel, kerosine and gasoline) have a total annual consumption of ∼2.8 billion tons (in 2019).^34^ Diesel mainly consists of linear paraffins in the range of C_10_–C_22_ and a cetane number of 48–55 (the cetane number is an indicator for the willingness of diesel fuel to self-ignite).^35–38^ Kerosene consists of C_8_–C_16_ paraffins with a higher content of iso-paraffins than diesel, which decreases its freezing point and makes it suitable for application as aviation fuel.^39,40^ Gasoline usually comprises hydrocarbons in the range of C_5_–C_11_.^41^ The specifications for gasoline are that it should have a research octane number (RON, classification number for spark-ignition characteristics) between 91 and 102, a maximum olefin content of 10–18 vol% and maximum aromatics content of 35–40 vol%, depending on the category of the gasoline fuel.^35^ Liquid fuels are in general a blend to meet the needs of the transportation industry while adhering to the requirements of environmental regulations.^42–44^ The latter are especially stringent regarding sulfur content, requiring ultra-low sulfur concentrations of 10 ppm or less.^45^ Fuels derived from synthesis gas, *via* the Fischer–Tropsch synthesis (FTS) process do not contain significant amounts of sulfur.^46^ Large FTS plants with consecutive hydroprocessing have been operated for decades by Shell, SASOL, Chevron and others, each producing yearly between 500 kt and 7.5 Mt of synthetic hydrocarbons including high quality diesel and kerosene.^47–51^

### 1.3. Production of synthesis gas

Synthesis gas (or in short “syngas”) can be produced from virtually any carbon-containing source. The present production of synthesis gas is mainly based on coal^52,53^ and natural gas.^54,55^ In the past years biomass-derived synthesis gas (bio-syngas) has gained significance.^56–59^1
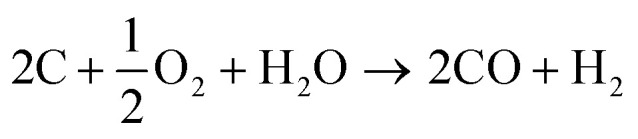

2 CH_4_ + CO_2_ → 2CO + 2H_2_

3
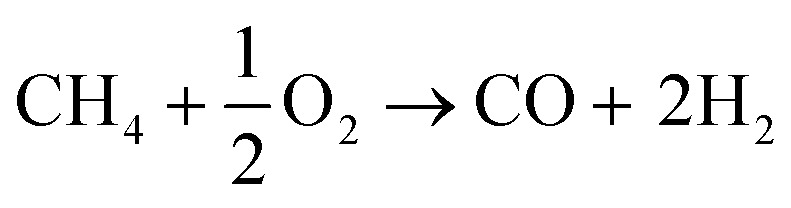

4 CH_4_ + H_2_O → CO + 3H_2_

5 (C_*x*_H_*y*_O_*z*_)_*n*_ + H_2_O/O_2_ → CO + H_2_ + CO_2_

6 CO + H_2_O ↔ CO_2_ + H_2_

An important implication of the source for producing synthesis gas is the resulting H_2_ to CO ratio. Typically, coal is converted by gasification as shown in eqn (1) resulting in a 1 : 2 molar ratio.^60^ The production of synthesis gas from methane yields an H_2_ : CO ratio between 1–3 mol mol^−1^, depending on the process: *via* dry reforming (eqn (2)) methane is converted together with carbon dioxide to synthesis gas with H_2_ : CO = 1 mol mol^−1^,^61,62^ whereas *via* partial oxidation (eqn (3)) and steam reforming of methane (eqn (4)) show H_2_ : CO ratios of 2 mol mol^−1^ and 3 mol mol^−1^, respectively.^63–66^ The gasification of biomass using either steam, oxygen, or a combination of both gives a high concentration of CO_2_ in the resulting bio-syngas (eqn (5)). This is related to the relatively high oxygen content in the biomass feedstock.^67,68^ The H_2_ : CO ratio and presence of CO_2_ are relevant for the follow-up processes. If needed the composition of the synthesis gas can be adjusted using the (reverse) water-gas-shift reaction (eqn (6)).

Sulfur compounds (*e.g.*, H_2_S or COS) in synthesis gas act as a poison to most synthesis gas conversion catalysts and might result from the feedstock.^69,70^ These can be removed using an absorber column with amine scrubbing.^71^ In contrast, removal of sulfur from heavier hydrocarbons in conventional refinery processes requires extensive effort. Here, the feedstock needs to be treated in the hydrodesulfurization (HDS) process.^72^

A major challenge with the production of synthesis gas from biomass is the competition with food and the impact on the environment by the use of monocropping and possible damage to the biodiversity.^73^ Furthermore, the availability of biomass for large scale synthesis gas production can be a hurdle considering the costs for transportation and the efficiency of land use.^74^ A technical challenge in bio-syngas production is catalyst deactivation by the formation of tar during biomass gasification. However, the use of a suitable catalyst in the steam reforming of biomass gives a tool to reduce the formation of tar drastically.^68,75^ Further impurities such as hydrochloric acid can be removed with amine scrubbing and an additional chloride guard bed.^71,76^

### 1.4. Primary conversion processes

#### 1.4.1. Methanol synthesis

Methanol is routinely produced with high selectivity from synthesis gas. The process to convert synthesis gas to methanol is typically operated at 30–50 bar and 220–300 °C. The methanol selectivity is larger than 99%.^77–79^ Methanol can be synthetized by hydrogenation of CO or CO_2_ (eqn (7) and (8)).^80–83^ Typically, a CO_2_-enriched (1–4% CO_2_ in the synthesis gas) synthesis gas is used.^84,85^

7 CO + 2H_2_ ↔ CH_3_OH

8 CO_2_ + 3H_2_ ↔ CH_3_OH + H_2_O

The main catalyst used in industry is copper based, with a typical composition of ∼50–60 wt% Cu, ∼30 wt% ZnO, and 10 wt% Al_2_O_3_.^77,86^ Copper in itself can catalyze the synthesis of methanol, but promotion with ZnO boosts its activity by more than an order of magnitude.^87–89^ It is well established that in CO_2_ enriched synthesis gas, CO_2_ is the predominant source of methanol formation. CO_2_ is formed during the reaction by the water gas shift reaction (eqn (6)) from CO and H_2_O, keeping the water level low.^84^ However, understanding of the nature of the synergetic interaction between Cu and ZnO remain the focus of a strong debate.^90^ Currently two main theories are prevalent: the first proposes that the active sites emerge from structural and/or electronic interactions at the Cu–ZnO interface^91–95^ and the second theory attributes the active sites to the presence of metallic Zn forming a Zn–Cu alloy or decorating the Cu surface.^96–102^

Copper-based methanol synthesis catalysts are employed by the industry due to their high activity at milder reaction conditions.^103^ Recently, research has focused on finding a methanol synthesis catalyst for using CO_2_ as main carbon source.^104^ Compared to the traditional feed three challenges must be met: decreased catalyst stability due to the high-water concentrations, a less favorable equilibrium and hence driving force for the reaction, and the side reaction forming CO *via* the reverse water gas shift reaction. A wide variety of materials has been proposed as candidates: intermetallic compounds such as Ni–Ga (ref. 105) or In–Pd,^106^ supported metal oxide nanoparticles MnO_*x*_/Co_3_O_4_ (ref. 107) or In_2_O_3_/ZrO_2_,^108^ solid solutions of metal oxides ZnO–ZrO_2_ (ref. 109) and transition-metal phosphide catalysts such as MoP.^110^

Methanol can be used as the starting point to produce DME (see chapter 2) or hydrocarbons in processes generally known as methanol-to-hydrocarbons (MTH), methanol-to-olefins (MTO), methanol-to-gasoline (MTG) and methanol-to-aromatics (MTA).^24,25^

#### 1.4.2. Fischer–Tropsch synthesis

Fischer–Tropsch synthesis (FTS) allows to convert synthesis gas into a mixture of hydrocarbons such as short olefins or paraffinic waxes. The mechanism involves a reaction of CH_*x*_ species on the catalyst's surface, a competition between C–C coupling and hydrogenation (chain growth and chain termination, respectively). The ratio of the rates of these processes is described as the chain growth probability (*α*) of the Anderson–Schulz–Flory (ASF) distribution.^111^ The ASF model allows to predict the distribution of products from the chain growth probability. A low value of *α* means the formation of mainly light products, whereas liquid or wax products are predominantly formed at medium and high values of *α*, respectively. The FTS always leads to a mixture of hydrocarbons with different chain lengths, with limits selectivity to certain product fractions.^112^

High temperature Fischer–Tropsch synthesis (HT-FTS) operates at 300–350 °C and about 20 bar utilizing an iron-based catalyst to produce hydrocarbons in the gasoline range (C_5_–C_11_) and light (C_2_–C_4_) olefins (eqn (9)).^49^ The active phase of catalyst is iron carbide.^113–117^ In industry, iron-based catalysts are often promoted with alkaline metals, such as potassium or sodium to increase activity, and selectivity to olefins.^118^ Additionally, copper is employed as promoter to increase the reducibility and SiO_2_ can be used as structural promoter.^49^ HT-FTS catalysts are also active in the water-gas-shift (WGS) reaction, converting CO and H_2_O into H_2_ and CO_2_ (eqn (6)).^22^ Promotion with sodium and sulfur allows decreasing the methane selectivity and increasing the C_2_–C_4_ olefin–paraffin ratio with respect to the ASF distribution.^114,119–122^ This enables 72% C_2_–C_4_ olefins formation, whereas according to the ASF 57% C_2_–C_4_ (olefins + paraffins) at maximum would be formed.^121^

9 *n*CO + 2*n*H_2_ → C_*n*_H_2*n*_ + *n*H_2_O

10 *n*CO + (2*n* + 2)H_2_ → C_*n*_H_2*n*+2_ + *n*H_2_O

Low Temperature Fischer Tropsch Synthesis (LT-FTS, eqn (10)) operates at 200–240 °C and 25–45 bar to produce waxes, and uses either supported iron- or cobalt-based, or precipitated bulk iron catalysts.^49^ Cobalt-based catalysts are often supported on metal oxide supports such as Al_2_O_3_, SiO_2_ or TiO_2_ with weight loadings of 20–30 wt% cobalt.^49,123–125^ A cobalt particle size of around 6 nm is optimum for both high activity and low methane selectivity.^126^ For cobalt particles smaller than 6 nm the surface coverage of CH_*x*_, OH_*x*_ and CO intermediates decreases, while the coverage with H increases, leading to a high methane selectivity and lowered CO conversion rates. Noble metal promoters such as Pt, Re or Ru are added to increase the reducibility of the cobalt oxide precursor catalyst. LT-FTS catalysts preferentially form long-chain hydrocarbons (>95% C_5+_ in the hydrocarbons and <3% CO_2_ due to their limited WGS activity).^125^

Ruthenium based FTS-catalysts can be operated at 140–220 °C and 15–100 bar.^127–129^ However, ruthenium is orders of magnitude more expensive than cobalt and less available.^118,130,131^ Nickel FTS-catalysts can display similar selectivity and activity as cobalt under similar reaction conditions.^132,133^ However, under high carbon monoxide partial pressure volatile nickel carbonyls are formed, leading to metal particle growth and/or nickel entrainment whereby the activity of the catalyst decreases over time.^118^ Bimetallic nickel–cobalt catalysts might be promising as they show increased activity and selectivity to C_5+_ hydrocarbons and better stability when supported on reducible oxide support materials.^132^

### 1.5. Secondary conversion processes

#### 1.5.1. Methanol dehydration to DME, olefins, and aromatics

The conversion of methanol to other oxygenates or hydrocarbons is often associated with dehydration of methanol and the elimination of water. Dimethyl ether is mainly produced from partial dehydration of methanol (eqn (11)), which is typically achieved at relatively low temperatures using mildly acidic sites. More details are given in chapter 2.

11 2CH_3_OH ↔ CH_3_OCH_3_ + H_2_O

Additionally, methanol can be converted into hydrocarbons such as short olefins or aromatics over the acid sites of a zeolite or other strong solid acids at higher temperatures (eqn (12) and (13)).

12 *n*CH_3_OH → (CH_2_)_*n*_ + *n*H_2_O

13
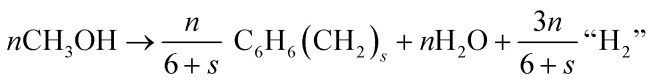
The term “H_2_” in eqn (13) describes the formation of either molecular hydrogen or the hydrogenation of an olefin molecule to the corresponding paraffin.

**Fig. 3:**
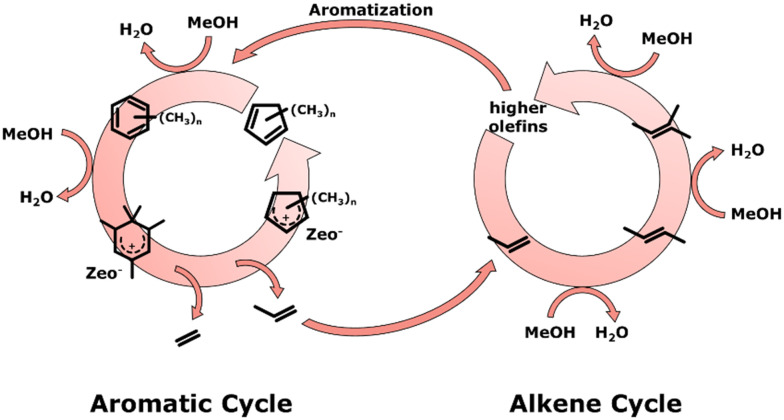
Illustration of the dual cycle mechanism consisting of the aromatic and alkene cycle.^134^

The conversion of methanol to olefins (MTO) and methanol to aromatics (MTA) is believed to follow the dual cycle mechanism (Fig. 3).^134–137^ In the alkene cycle, short olefins are alkylated by the addition of a CH_2_ group that is transferred from methanol, forming longer chain olefins and water. The products in the alkene cycle are higher olefins that either dealkylate forming short olefins or undergo aromatization and enter the aromatic cycle. In the aromatic cycle, light aromatics are alkylated by CH_2_ groups from methanol to form poly-alkylated aromatic species and water. These poly-alkylated aromatic species are protonated by the Brønsted acid sites of the catalyst, followed by dealkylation and consecutive deprotonation. During the dealkylation, short olefins are released which can either enter back into the alkene cycle or yield the final products.

Zeolites with 8-membered ring pores such as SAPO-34 or H-SSZ-13 show high selectivities to short olefins (70% to 96%) at full methanol conversion,^138,139^ when operated at high temperature (300–450 °C) and atmospheric pressure.^140,141^ The zeolites used in the MTA reaction usually are 10-membered ring zeolites such as H-ZSM-5 and TNU-9 or 12-membered ring zeolite such as zeolite type beta or mordenite^142–144^ The MTA reaction yields a variety of products, ranging from 2–40% C_2_–C_4_ olefins, 8–51% aliphatic hydrocarbons with more five carbon atoms (C_5+_) to 17–50% aromatics.^144,145^ The strongly different product spectra of the MTO and the MTA process can be explained by the pore dimensions and topology of the zeolites.^146–148^ The aromatic species formed during the MTO reaction are retained in the small zeolite cavities and participate in alkylation and de-alkylation in the aromatic cycle. The pores of 10- and 12-membered ring zeolites used in the MTA process are wide enough to release the aromatic molecules.

During the MTO and MTA process the zeolite is rapidly deactivated mostly by formation of coke, blocking the active sites. Catalyst lifetimes vary from 20–200 h on stream, depending on material composition, crystallite size, acid site density, porosity and reaction conditions.^138,141,145,149–153^ Co-feeding water increases the lifetime of the catalyst, but reduces its activity due to co-adsorption on the acid sites.^154^ Also, here promoters can play a role. Partially replacing the protons of the Brønsted acid sites with zinc-ions in H-ZSM-5, led to increased selectivity towards aromatics and reduced paraffin selectivity in the MTA process.^155–157^

#### 1.5.2. Cracking and isomerization

Cracking is the fragmentation of long hydrocarbons into smaller molecules, whereas isomerization involves the skeletal rearrangement of hydrocarbon molecules. These reactions are often applied in the petrochemical industry to match the requirements for transportation fuels. Gasoline and diesel fuels are being used to drive spark- and self-ignition engines, respectively. By increasing branching and decreasing chain length the octane number of gasoline increases, whereas the cetane number of diesel increases with chain length and reduced number of branches.^158,159^ The most common catalysts for cracking and isomerization are zeolites and catalysts that next to the zeolite contain a metal or metal sulfide.

**Fig. 4:**
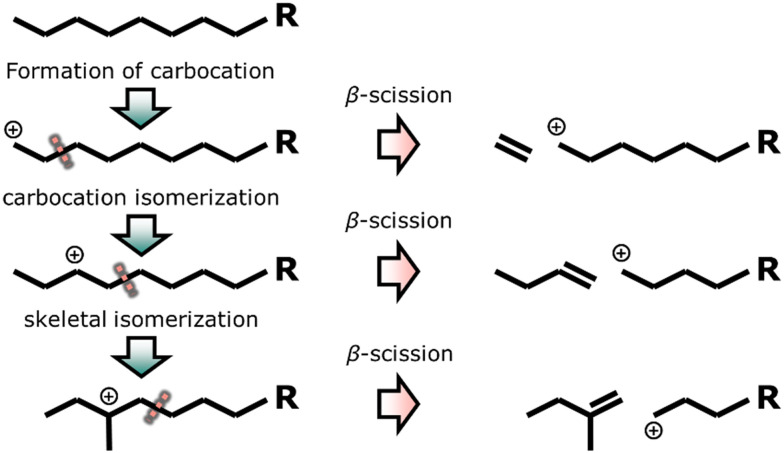
Catalytic cracking and isomerization with the formation of carbocations, isomerization and consecutive cracking with different positions of the positive charge.^160,161^

The mechanism of mono-functional acid catalyzed cracking and isomerization over zeolites involves carbocations at elevated temperatures (Fig. 4).^160,161^ A primary carbocation (positive charge on a carbon atom at the end of the chain) is relatively unstable and undergoes carbocation isomerization, resulting in a more stable secondary carbocation (positive charge being stabilized by two alkyl groups). Tertiary carbocations show even higher stability and are formed from skeletal rearrangement of secondary carbocations. These carbocations can be cracked at the β-position (one carbon atom further than positive charge, called β-scission) forming olefins and another carbocation. Acid cracking and isomerization yields few *n*-paraffins (alkanes) and gases, whereas high yields of aromatics, olefins and *i*-paraffins are produced.^162^ The majority of the hydrocarbon products is isomerized due to the high stability of the tertiary carbocation intermediate.^163^

Alternatively, bifunctional catalysts can be used for cracking and isomerization at lower temperatures. The feed molecules are dehydrogenated on the metal sites forming olefins and hydrogen. The olefin molecules undergo carbocation formation, carbocation and skeletal isomerization, and β-scission to some extent similar to the mechanism of acid cracking and isomerization on the acid sites of the zeolite. The resulting olefins, however, are hydrogenated on the metal sites, forming paraffins.^161^ This means that transport of molecules between the two different sites is very important.

Bifunctional cracking and isomerization are performed in the presence of hydrogen and at pressures between 5–150 bar,^14,164,165^ in the so-called hydrocracking or hydroisomerization process. The product spectrum strongly depends on the reaction temperature. High reaction temperatures (between 300 °C and 400 °C) favor hydrocracking,^165–168^ whereas hydroisomerization is more likely at lower temperatures (between 200 °C and 260 °C).^14,164,169–171^ The distance between the metal and acid site is crucial to achieve high yields of branched isomers in the hydroisomerization reaction. Relatively large distances, in the range of micrometers, can cause strong concentration gradients of intermediates and reactants within the catalyst.^172–174^ Closest proximity, however, can lead to an increased degree of cracking forming more gaseous products.^14^

### 1.6. Side reactions

#### 1.6.1. Water-gas-shift reaction

The reaction of carbon monoxide and water to form carbon dioxide and hydrogen (eqn (6)), known as the water-gas-shift (WGS) reaction, is used traditionally in industry on a scale of ∼50 million tons per year.^175^ Steam reforming of natural gas yields a mixture primarily consisting of hydrogen and carbon monoxide. This carbon monoxide is further converted using WGS to produce additional hydrogen and carbon dioxide.^176^ The WGS reaction is also relevant to adjusting the H_2_/CO ratio of synthesis gas. The WGS reaction is readily catalyzed by metals and metal oxides. Catalysts based on iron oxides (previously also chromium-based catalysts) are employed at intermediate temperatures (400–500 °C) and copper-based catalysts at lower temperatures (150–200 °C).^177,178^ This reaction is moderately exothermic, favored thermodynamically at lower temperatures and kinetically at elevated temperatures.^179^

However, the WGS reaction is usually undesired when converting CO-based synthesis gas. Although the feed in this case does not contain water, product formation is often accompanied by water formation, especially in FT. The presence of both water and carbon monoxide can lead to the production of important concentrations of CO_2_, for instance in HT-FTS.^180^ This has a negative effect on the efficiency of the process, and hence a common challenge in these processes is to limit the WGS activity of the catalyst.

#### 1.6.2. Olefin secondary hydrogenation

Hydrogenation of desired products or reaction intermediates (denoted as secondary hydrogenation), most notably of olefins, can decrease the final yield of the desired product. For instance, when a cobalt catalyst is used in FTS, 1-olefins can re-adsorb on the metal catalyst surface and either participate in further chain growth or undergo secondary hydrogenation forming paraffins.^181–186^ The presence of alkaline promoters on iron-based FT catalysts can reduce secondary hydrogenation activity.^187–189^ They increase the conversion of metallic iron into iron carbide,^113^ from which it was concluded that secondary hydrogenation is predominantly catalyzed by metallic iron sites.^190,191^

OX–ZEO catalysts consist of metal oxides combined with zeolites and can convert synthesis gas to olefins and aromatics.^192^ In the first step synthesis gas is converted into reactive intermediates such as methanol, dimethyl ether, or ketene over the metal oxide, followed by the formation of olefins on the zeolites. Olefins also act as intermediates in the OX–ZEO to aromatics process.^193,194^ Metal oxide functions with a high hydrogenation activity can cause secondary hydrogenation of olefins to paraffins.^195^ The hydrogenation of aromatics requires the presence noble metals such as platinum or palladium^196,197^ and does not take place in the OX–ZEO process with the commonly used catalysts.^198^ A detailed analysis of secondary hydrogenation can be found in chapter 3.3 and 4.3.

#### 1.6.3. Coke formation

**Fig. 5:**
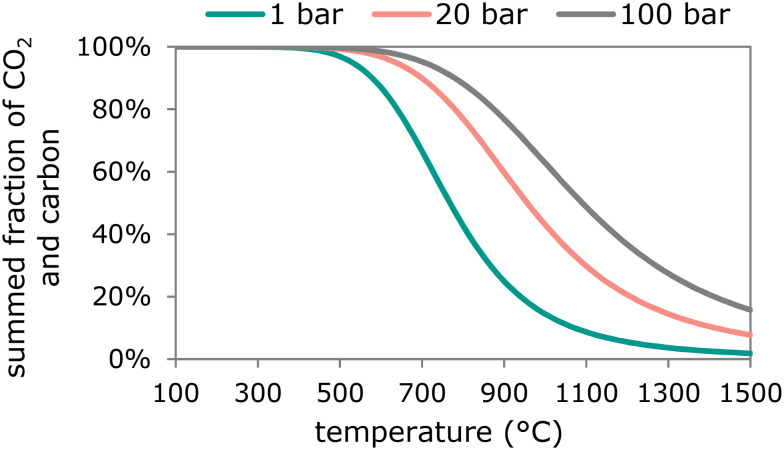
Fraction of CO_2_ + C at equilibrium as a function of temperature with CO, C and CO_2_ at either 1 bar, 20 bar or 100 bar pressure.

The formation of coke is a major cause for catalyst deactivation in synthesis gas and hydrocarbon conversion reactions.^121,199^ Either carbon or polyaromatic hydrocarbons can be formed. On metallic catalysts carbon may be formed by the disproportionation of carbon monoxide into carbon dioxide and solid carbon (Boudouard reaction, eqn (14)) and the extent to which this may occur depends on the reaction temperature and pressure (Fig. 5, calculated with Outotec HSC 9.6.1).

14 2CO(g) ⇄ CO_2_(g) + C(s)

Iron-based Fischer–Tropsch catalysts transform into iron-carbide species under operation conditions.^22,113^ The formation of the active iron-carbide phase is often accompanied by the Boudouard reaction leading to carbon deposition on the active site. The presence of alkaline promoters such as K or Na increases the rate of carbon deposition, whereas the type of iron carbide does not influence the carbon deposition.^200^ Additionally, carbon deposition in the pores of an unsupported iron-based Fischer–Tropsch catalyst or the transformation into iron-carbide can lead to fragmentation of the catalyst particles, due to strain effects.^22,201,202^

Alternatively, heavy hydrocarbons formed by oligomerization in acid catalyzed cracking and isomerization can condense onto and hence deactivate active sites at temperatures below 200 °C.^203^ At high temperatures (350 °C and above) hydride transfer reactions take place causing the formation of polyaromatic hydrocarbons.^203,204^ The temperature for the formation of polyaromatic hydrocarbon species is reduced for hydrocracking and hydroisomerization, because (de)hydrogenation is catalyzed by metal sites.^203^

The mechanism of the MTO and MTA reactions is based on the alkylation and de-alkylation of light aromatic species inside zeolite crystals. The main cause for catalyst deactivation is the formation of large and heavy poly-aromatic hydrocarbons inside the zeolite pores or cavities, limiting accessibility to the acid sites of the zeolites.^205,206^ Using an H-SSZ-13 zeolite in the MTO process, it was shown that also at lower reaction temperatures pore filling of the zeolites with methylated bicyclic aromatics plays a role, whereas at higher temperatures the deactivation is caused by the formation of 3- and 4-cyclic aromatic species.^207^

In the following sections we analyze the recent literature of bifunctional catalysis for the conversion of synthesis gas to DME, short olefins, aromatics, and gasoline. Additionally, we compare the performance of these catalysts with established processes consisting of sequential individual catalytic steps in terms of overall selectivity and conversion.

## 2. DME

**Fig. 6:**
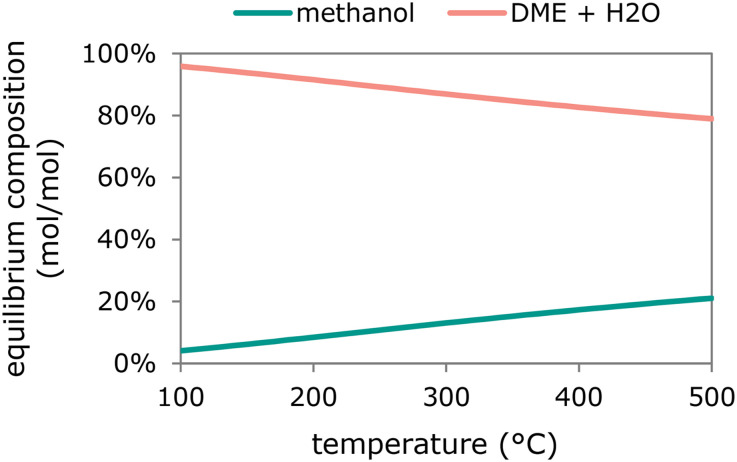
Equilibrium composition of methanol dehydration to DME and water as function of reaction temperature calculated at 1 bar pressure (calculated with Outotec HSC 9.6.1).

Methanol dehydration to DME is usually performed at atmospheric pressure, high space velocities and temperatures between 190 °C and 400 °C.^208–210^ The catalysts most widely used are solid oxide acids such as γ-Al_2_O_3_ or aluminosilicates, or zeolites.^208,211^ The active sites can be both Lewis and Brønsted acid sites.^212^ Processes using γ-Al_2_O_3_ catalysts proceed at the higher end of the mentioned temperature range.^209,213^ γ-Al_2_O_3_ has mainly Lewis acid sites, which might adsorb the formed water particularly at low temperatures, inhibiting the reaction with methanol.^209^ Increased reaction temperatures facilitate the desorption of water from the acid sites of the Al_2_O_3_ catalysts, but also decrease the maximum attainable one-pass DME yield due to equilibrium limitations (Fig. 6).

Although for methanol dehydration most commonly γ-Al_2_O_3_ is used, other acidic compounds can also be used as catalyst.^214^ Mixed metal oxides such as aluminosilicates and ZrO_2_/TiO_2_ have Brønsted acid sites next to Lewis acid sites, and display enhanced activity and stability compared to Al_2_O_3_ under the same reaction conditions.^208,209^ Zeolites have both Lewis and Brønsted acid sites and allow lower operation temperatures. Additionally, the strong Brønsted acid sites might allow sequential olefin formation at higher temperatures.^214^

### 2.1. Recent developments

Direct DME synthesis has attracted large interest, which is reflected by the extensive investments in direct DME synthesis pilot plants^215–217^ and several academic reviews on DME synthesis published in the recent years.^211,218–223^ Based on these reviews and several other publications, we give an overview of the optimal reaction conditions as well as catalysts.

Colloidal synthesis of nanoparticles has emerged as a tool to prepare and understand catalytic model systems.^224^ Pre-forming the nanoparticles in solution and then depositing them onto a support material enables the preparation of monodisperse, single crystalline, and size-controlled nanoparticles, which is rather challenging for conventional synthesis techniques.^225^ For bifunctional catalysts, colloidal nanoparticles have been employed as a strategy to avoid structure sensitivity effects of the metal-based methanol synthesis catalysts and control its proximity to the acid sites.^226^ Monodisperse colloidal Cu–ZnO-based nanoparticles were either directly supported on the dehydration catalyst (γ-Al_2_O_3_) or mixed with the dehydration catalyst.^227^ Directly supporting the nanoparticles on the solid acid caused partial blockage of the acid sites and a slight decrease in DME selectivity (64% to 59% DME selectivity).

The same approach has been used to study Pd–Ga-based colloidal nanoparticles, as methanol synthesis catalyst from CO and H_2_, supported on γ-Al_2_O_3_.^228^ The interest in Pd–Ga systems arises from its activity in CO_2_ hydrogenation to methanol.^229,230^ The Pd–Ga/γ-Al_2_O_3_ system showed good stability. However, hydrocarbon selectivity remained an issue over the whole temperature range. Methane content increased from 12% at 250 °C to 43% at 300 °C, while Cu–ZnO-based catalyst produced only 1.2% at 250 °C and 8.8% at 300 °C. High methane yields has been reported to be a general problem of Pd-based DME catalysts.^231^

The use of core–shell systems is a popular strategy to circumvent Cu sintering. Typically, the metal-based core is encapsulated by a solid acid shell.^232^ This forces also the methanol formed on the core catalyst to pass through the acidic material before leaving the catalyst system, leading to a high DME selectivity.^233^ The catalytic performance (275 °C, 35 bar, H_2_ : CO_*x*_ = 3 mol mol^−1^ and TOS = 24 h) of CuO–ZnO–ZrO_2_ and SAPO-11 was studied for a physical mixture of the two components, and for a core–shell catalyst with the Cu-based catalyst being covered by SAPO-11.^234^ The highest DME yields were obtained at a CuO–ZnO–ZrO_2_ to SAPO-11 weight ratio of 0.5. The core–shell catalyst showed a more stable catalytic performance than the physical mixture, having a relative decrease in DME yield at the end of the experiment (24 h time on stream) of 22% against 33% for the physical mixture. By comparing the acidity of the catalysts before and after reaction, it was observed that the core–shell catalysts lost around 10% of the initial acid sites after 24 h on stream, while the physical mixture lost around 26%. The loss of acid sites was identified as coke deposition, being lower for the core–shell catalyst. Physical separation of the metallic and acid functions by an intermediate silica layer contributes to reducing coke deposition on the SAPO-11, and therefore to preserve its acidity.

The strategy of a porous intermediate layer in a core–shell catalysts has been previously explored;^235,236^ a silica layer was deposited over the Cu-based catalyst to avoid damaging the integrity of the catalyst while depositing the solid acid overlayer. Alternatively, a mesoporous alumina interlayer has been also employed, on which silicotungstic acid is deposited to improve the shell's acidity and the catalysts DME selectivity.^237^

However, the use of a protective silica layer can cover part of the active sites of the methanol synthesis catalyst,^233,238^ resulting in lower CO conversion. To avoid this, a different coating method was reported in which various solvents (ethanol, water, methanol, and ethylene glycol) were used as binder to coat an H-ZSM-5 shell on a Cu–ZnO-based catalyst.^239^ Ethanol as a solvent showed the best performance, although this could not be explained by more exposed metal sites based on the characterization results.

The use of ultra-small (<5 nm) ZSM-5 zeolite crystals placed on a CuO–ZnO–Al_2_O_3_ methanol synthesis catalyst showed better activity, selectivity to DME, and stability than the methanol synthesis catalyst combined with amorphous aluminosilicate or ZSM-5 zeolites with 20–500 nm crystallite size.^240^ It was concluded that the ultra-small ZSM-5 nano particles had superior diffusion properties compared to larger crystals. Additionally, the medium strength of the Brønsted acid sites resulting from the small crystallite size did not facilitate further dehydration of DME to olefins.

Electrospinning has been employed for the synthesis of various fibrous high-performance materials,^241,242^ in this case for the design of a bifunctional catalysts. A fibrillar system has been reported which circumvents diffusion limitations while maintaining a close contact within functionalities.^243^ The Cu–ZnO/ZrO_2_–ZSM-5 fibrillar bifunctional catalyst was prepared by using an electrospinning technique. The polymeric filaments after calcination, resulted in this case in homogeneous zirconia-based fibers (with a diameter of 1.5 μm) and well-distributed Cu–ZnO and zeolite aggregates throughout the fibers of the bifunctional catalyst. The catalyst showed high DME yields (59–63%), with a low zeolite content of 10 wt%. This could be attributed to the high dispersion of the zeolite over the fibers, and the fact that the methanol synthesis function was not affected by the addition of the zeolite during synthesis. The pressure drop inside the fixed-bed reactor was theoretically calculated for the fibrillar structured catalyst with micrometric size and for the powder catalyst with the same effective dimension. The calculation results showed 5000 times less pressure-drop for the fibrillar packed bed than for a packed bed of spherical particles (0.3 *vs.* 1650 bar m^−1^). Longer tests than the reported 4 hours on stream might give more insight into the stability of this material.

*In situ* removal of water during DME synthesis, often referred as sorption enhanced dimethyl ether synthesis, has emerged as a relatively new approach to avoid the detrimental effects of water on the catalyst and boost the DME selectivity by inhibiting the water-gas-shift reaction. This idea is promising for process intensification and can be applied to different reactions in which water is a by-product as recently reviewed.^244,245^ Water can be removed from the catalyst bed using membrane technology or selective adsorption.^246^ The former requires large H_2_O partial pressures differences and high permselectivity of water over the reactants, the latter is preferred at low H_2_O partial pressures (<1 bar). Theoretical simulations have confirmed higher DME yields under H_2_O removal conditions, particularly upon addition of CO_2_ due to an increased methanol production and preventing the water-gas-shift reaction.^247^

Experimentally, enhanced DME production has been reported for a commercial copper-based catalyst mixed with a water absorbent material (commercial zeolite LTA-type with 3 Å pore size).^245,248^ Adsorption of water by the zeolite during DME synthesis led to a decrease in CO_2_ formation. The DME yield was 65% at around 70% CO conversion (275 °C, 25 bar and H_2_ : CO = 2 v/v). Upon saturation of the zeolite after some minutes of the reaction, a regeneration step was carried out by switching to nitrogen, depressurizing to 1.7 bar and heating to 400 °C. More recently, the same concept has been studied using a Cu–ZnO-based catalyst in combination with γ-Al_2_O_3_ as solid acid and a zeolite 3A as water sorbent.^249^ The methanol catalyst alone showed a carbon conversion of 9.7% and 100% selectivity to methanol at 270 °C and 25 bar with a syngas composition of CO : CO_2_ : H_2_ = 1 : 1.9 : 7.7 (v/v/v). When the methanol synthesis catalyst was combined with the methanol dehydration catalyst and the water sorbent zeolite at 275 °C, the carbon conversion increased to 83% (54% CO conversion and 97% CO_2_ conversion) with a DME selectivity of 99% in the early stage of the reaction (∼20 min). After the zeolite was saturated (after ∼100 min.) the carbon conversion dropped to 19% (41% CO conversion and 8% CO_2_ conversion) and DME was formed with 81% selectivity.

However, information on the stability of the catalysts in these studies is lacking, especially after regeneration cycles. Identifying sorbent materials that operate under DME synthesis conditions without suffering deactivation remains challenging.^250^ Research efforts have been focused on improving the regeneration procedure.^251^ It has been shown that a pressure swing (from 25 bar at reaction conditions to 1–3 bar) followed by purging with an inert gas can remove the water of the zeolite 3A and regenerate the activity without changing the temperature of the reactor. This swing process to remove the adsorbed water required 1 h, which is faster than the alternative thermal treatment at 400 °C which can require 6 h.

The conversion of CO_2_ or CO_2_-containing synthesis gas in the direct DME synthesis has attracted recently attention in research.^252–258^ Published data has shown that 48% CO_2_ conversion^252,256^ and high DME selectivities up to 100% (ref. 255 and 257) can be reached. Additionally, a comparison between CO_2_-rich and CO-rich synthesis gas revealed that a CO : CO_2_-ratio of 1 : 4 (v/v) in the synthesis gas led to higher conversion (65.6%, sum of CO + CO_2_) compared to a CO : CO_2_-ratio of 4 : 1 (v/v) (35.4% conversion) The DME selectivity resulting from the CO_2_-rich synthesis gas was slightly lower (73.2% compared to 88.7%), however, the yield of DME was higher (48% compared to 31.4%).^237^ Direct CO_2_ hydrogenation to DME also showed advantages in energy efficiency and net CO_2_ mitigation in a techno-economic study compared to different routes (indirect route *via* CO or direct CO_2_ hydrogenation).^259^ Compared to methanol synthesis from CO_2_, direct DME synthesis can result in higher CO_2_ conversions (+20%) and higher yields of valuable products (+70%).^260^

### 2.2. Benefits

**Fig. 7:**
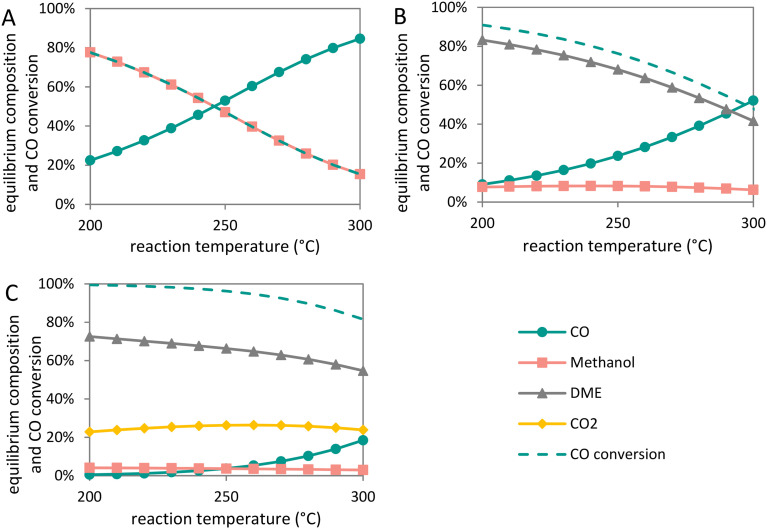
Equilibrium composition (based on carbon atoms) and CO conversion as a function of temperature at 40 bar for carbon species involved in A: methanol synthesis, B: direct synthesis of DME without water-gas-shift reaction, and C: direct synthesis of DME with water-gas-shift reaction. The thermodynamic calculation considering all species in the gas phase was carried out with a synthesis gas composition of H_2_ : CO = 2 v/v and as possible products methanol (A) and additionally DME (B) and DME and CO_2_ (C). HSC software from Outotec (v 7.14) was used to perform the calculations.

Fig. 7 shows the CO conversion and yields of methanol, DME and CO_2_ as function of reaction temperature at 40 bar total pressure for methanol synthesis (Fig. 7-A), direct DME synthesis without WGS (Fig. 7-B) and direct DME synthesis with WGS (Fig. 7-C) in equilibrium (calculated with Outotec HSC 9.6.1). For the methanol synthesis the CO conversion is limited to 40% at 260 °C and 40 bar. If instead of pure CO (also) CO_2_ is added to the synthesis gas feed, the conversions are even lower. The direct DME synthesis shows an equilibrium conversion of 72%, whereas the direct DME synthesis with WGS shows a maximum CO conversion of 95% at these conditions. Removing methanol by subsequent dehydration hence increases the conversion of synthesis gas, reaching CO conversions as high as 96% and DME selectivities up to 87%.^261,262^ The additional removal of water *via* the WGS reaction drives the equilibrium to even higher conversions. Although the WGS reaction compromises the selectivity to DME, it increases the DME yield per single pass, especially at higher temperatures.

Cu-based methanol synthesis catalysts display a high water-gas-shift activity.^263–265^ Water formed during dehydration can react with CO forming CO_2_ and H_2_ (see section 1.3 eqn (6)). This can be beneficial when using hydrogen-lean synthesis gas. Furthermore, the presence of a few percent of CO_2_ enhances the activity of the methanol synthesis catalyst.^90^ High water concentrations result in accelerated deactivation, which can be circumvented by water removal *via* the WGS reaction or *via* a membrane.^266,267^

### 2.3. Challenges

Methanol dehydration to form DME can be catalyzed by Brønsted as well as Lewis acid sites, and all acid strengths. However, particularly at high temperatures, strong acid sites can facilitate the further dehydration of DME to olefins and other hydrocarbons, compromising the DME yield.^209,268,269^ This does not represent a problem for the dual reactor process, since the methanol dehydration step can be operated at relatively low temperatures (≤200 °C, Fig. 6). At higher temperatures needed for methanol synthesis, less strong acid sites are preferred. Indeed, acid sites with a weak to medium strength have shown an excellent selectivity to DME under direct DME synthesis conditions.^240,261,270^

Industrial methanol synthesis catalysts are copper-based.^86^ Copper nanoparticle growth and hence loss of active metal surface area, is the main deactivation mechanism.^271^ It is enhanced by higher water concentrations, to which it will be exposed when used in direct DME synthesis or in CO_2_ rich feeds.^272,273^ Faster deactivation was observed when co-feeding water using a Cu–ZnO methanol synthesis catalyst only.^274,275^ Recently, the stability of a Cu–ZnO catalyst physically mixed with a ZSM-5 zeolite was studied under DME synthesis conditions (260 °C, 20 bar, 90 000 or 3600 cm^3^ g_cat_^−1^ h^−1^ and H_2_ : CO = 2 v/v) by *in situ* synchrotron-based EXAFS and XRD experiments.^276^ Results show an increase of the copper crystallite size from 9 nm to 12 nm during the first hours under reaction conditions, while copper remained in the metallic state within the technique's detection limit. Decreasing the gas space velocity or co-feeding water led to larger crystallite sizes, 17 nm and 20 nm respectively. The authors concluded that the water generated during DME synthesis has a detrimental effect in the stability of the Cu–ZnO catalyst mainly by particle growth.

For the methanol dehydration catalysts, the challenges vary according to the nature of the material. Zeolites in the proton form typically have strong Brønsted acid sites which can lead to further DME dehydration to hydrocarbons, although some strategies have been developed to tune the zeolite acidity and to improve DME yields.^277,278^ Another main challenge is the microporous structure of zeolites which can limit the diffusion of reactants and products leading to hydrocarbon and coke formation, deactivating and blocking the active sites.^279^ H-ZSM-5 in a physical mixture with Cu–ZnO catalysts showed a decrease in activity of ∼20% due to accumulation of hydrocarbon species formed in the pores (250 °C, 10 bar and TOS = 100 h).^280^ The synthesis gas composition in this case was important for the zeolite stability, the presence of CO_2_ directly affected the partial pressure of water and hence aided to avoid accumulation of carbonaceous species in the pores.

γ-Al_2_O_3_ is a very selective solid acid catalyst to produce DME due to its mild Lewis acid sites, active for methanol dehydration. However, it can loss activity in the presence of water due to competitive water adsorption on the acid sites or by recrystallization.^280^ The γ-Al_2_O_3_ to boehmite phase transition has been investigated in the range of 250–400 °C and H_2_O partial pressures up to 15 bar.^281,282^ Results over γ-Al_2_O_3_ at 250 °C and water partial pressure of 13–14 bar led to the conversion of γ-Al_2_O_3_ into γ-AlO(OH). This was linked to a decrease in catalytic activity of methanol dehydration, from ∼60% methanol conversion to ∼15%. However, the phase transition was reversible under more standard reaction conditions or calcination at 350 °C, recovering its catalytic activity. Niobium oxide-based dehydration catalysts are less active but can form a stable NbO_4_–H_2_O phase and do not show water induced deactivation.^270,283–288^

The interaction between both catalytic materials can result in activity and/or selectivity loss, therefore the distance between functionalities is a key factor for the stability of the catalyst. Two distances in the micrometer range have been studied by co-tableting powders with different sieve fractions of a Cu–ZnO–Al_2_O_3_ methanol synthesis catalyst and silica-alumina dehydration catalyst.^289^ A fine sieve fraction of 50–100 μm of the individual catalysts and a coarse sieve fraction of 600–1000 μm were used to prepare bifunctional catalysts. During direct DME synthesis (285 °C, 60 bar, TOS = 700–800 h), the finer particle catalyst deactivated faster than the catalyst pelletized from larger particles. Characterization of the used catalysts revealed migration of zinc from the methanol synthesis catalyst into the dehydration component and silicon from the dehydration component diffused into the methanol synthesis catalyst particles. The authors concluded that the faster poisoning of the more finely sieved catalyst relates to the larger contact area between the two catalyst materials. Analog, it has been observed that species exchange between the solid acid and Cu–ZnO catalyst with H-ZSM-5 as solid acid.^290–293^ The extent of deactivation was linked to the amount of zeolite's extra-framework Al species and surface acid sites.^292,294^ Migration of copper from the methanol synthesis catalyst to niobium-based solid acids has also been observed after DME synthesis (260 °C, 40 bar, H_2_ : CO = 2 v/v and TOS = 120 h).^270^

### 2.4. Process comparison

**Fig. 8:**
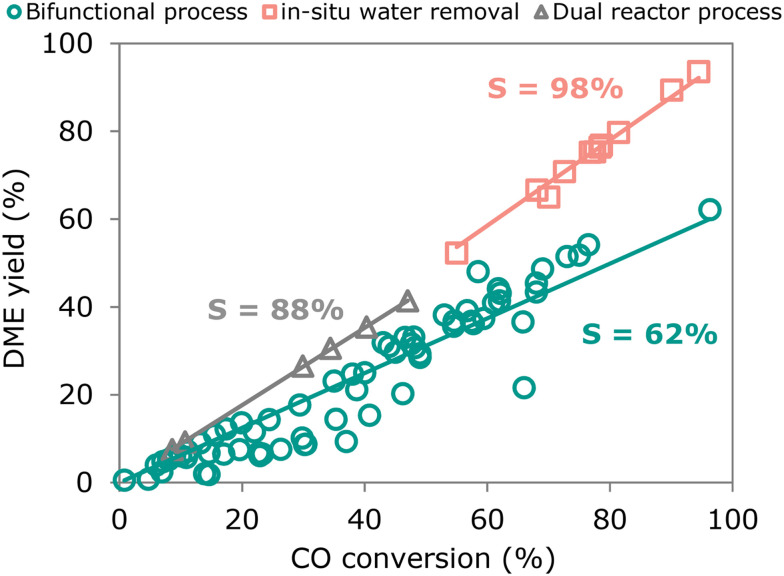
DME yield as a function of CO conversion for the dual reactor process, bifunctional process, and bifunctional process with *in situ* removal of water. Data points were obtained from recent reports in which DME is the principal product, the complete data set with the corresponding references can be found in ESI.†

The direct production of DME from synthesis gas can be effectively carried out by use of bifunctional catalysts. Combining both functionalities in a single catalyst comes with clear advantages, in particular a higher conversion of CO in a single pass, over the dual reactor process. In Fig. 8 we have gathered experimental data for both types of processes from published literature, showing the DME yield as a function of CO conversion. The slope of the line corresponds to the overall selectivity with which CO is converted to DME. Complete data sets can be found in the ESI.† Values for the dual reactor process were obtained by combining the maximum reported conversion and selectivity values from the methanol synthesis and methanol dehydration reactions, respectively. The resulting slope of the trendline (in grey) shows an overall selectivity of 88% for the dual reactor process with the methanol synthesis being detached from the methanol dehydration reaction in separate reactors.

For the bifunctional process, most catalysts follow a similar trend (in green) independently of the catalyst synthesis method, with a selectivity of 62%. This is lower than the dual reactor process due to the production of CO_2_ from the water-gas-shift reaction at the expense of DME yield. Still, the bifunctional process allows to reach much higher CO conversions hence also higher DME yields in a single one-pass conversion compared to the dual reactor process. Bifunctional systems have catalysts consisting of physical mixtures or core–shell catalysts. This uniform trend also indicates that the distance between functionalities does not have a strong effect on the selectivity to DME. This might be explained by fast diffusion of chemical species in this process (*i.e.*, methanol and DME) in contrast to species in other bifunctional processes (*e.g.*, long-chain paraffins or aromatic compounds). This in agreement with the recent work of Li *et al.*^295^ A physical mixture of methanol synthesis and dehydration catalysts displays an effective system for high DME yields. However, as discussed in the previous section, a very fine physical mixture might negatively affect the stability of the catalyst.^289^

Within the different catalysts' configurations for bifunctional process, the *in situ* water removal strategy^248–250^ follows a different trend (in red) and therefore has been plotted separately. Here the slope corresponds to a 98% DME selectivity, with also high CO conversion. By capturing the formed water, most of the water-gas-shift reaction is inhibited and thus CO is converted mainly to DME. This seemingly is the most attractive pathway for an efficient DME production. However, aspects such as cost, energy consumption and ease of operation might be significant disadvantages of this method.

Unfortunately, few studies report long time-on-stream results, which makes it difficult to assess the stability of the various catalysts configurations. At least 100 h-on-stream results would give a good indication of the stability of the catalysts. Sintering of the metal functionality in the methanol synthesis catalyst and ion migration within functionalities seem to be the main phenomena responsible for activity loss. Solid acids with mild acid strength are readily active and selective for DME synthesis. Their stability seems less problematic than that of the methanol synthesis catalyst, the copper and copper–zinc interphase in these catalysts are susceptible to crystallite growth in the presence of water.

## 3. Olefins

### 3.1. Recent developments

**Fig. 9:**
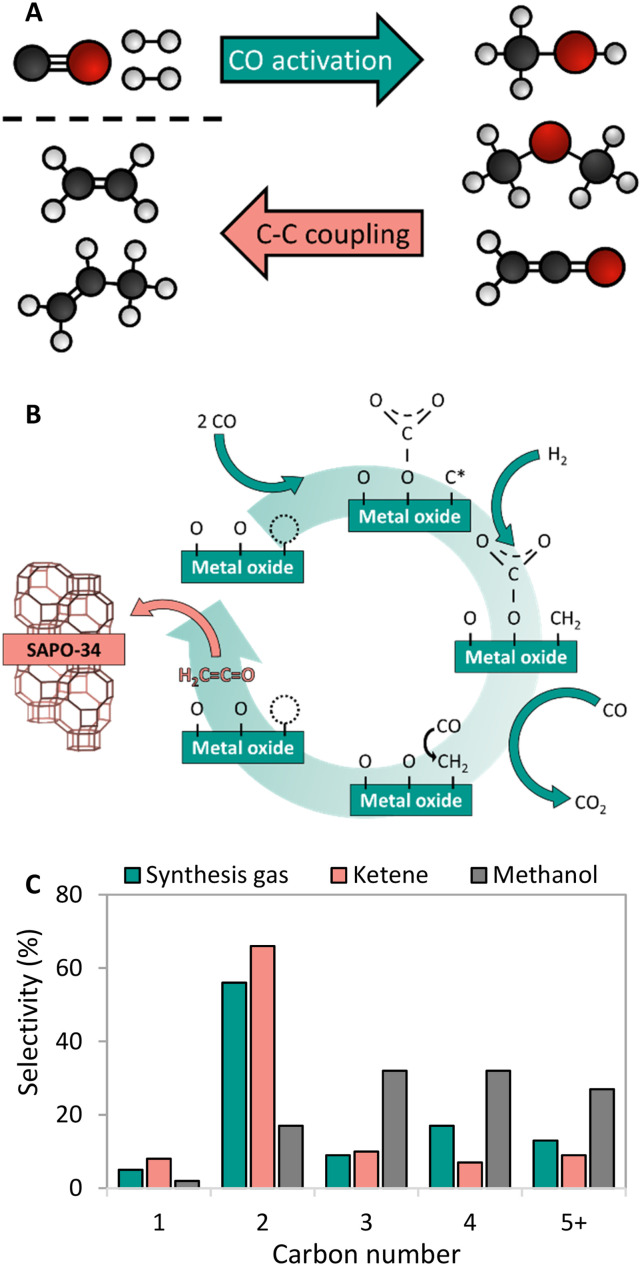
A: General reaction scheme of the OX–ZEO process, whereas CO activation takes place on a metal oxide forming oxygenate intermediates followed by C–C coupling on a zeolite.^298^ B: Proposed mechanism of CO activation over a metal oxide catalyst forming ketene intermediates.^306^ C: Hydrocarbon product spectrum (CO_2_ free) when either feeding synthesis gas to an OX–ZEO catalyst (green bars), or ketene (red bars) or methanol (grey bars) to a MOR-zeolite with 8 membered ring pores and 12 membered ring pores being accessible. For the experiments with synthesis gas feed zinc–chromium oxide particles were attached to the MOR-zeolite for synthesis gas activation. Synthesis gas over ZnCrO_*x*_/MOR gave similar products as ketene fed over MOR-zeolite with high C_2_ selectivities.^297^

A process called OX–ZEO, developed by the groups of Prof. Bao and Prof. Wang, to convert synthesis gas to short olefins in the range of C_2_–C_4_ can be considered a breakthrough in bifunctional catalysis.^192,296^ The OX–ZEO catalysts consist of metal oxides (based on for instance zinc, zirconium and/or chromium oxides) and a zeolite. Synthesis gas is first converted over the CO activation catalyst (metal oxide) to reactive oxygenate intermediates such as methanol/dimethyl ether or ketene (Fig. 9-A).^297–300^ These intermediates are further converted to short olefins *via* C–C coupling over the acid sites of a zeolite with usually 8-membered ring pores,^298,301–303^ such as SAPO-34 or H-SSZ-13, which are well known in the methanol-to-olefins reaction for their high selectivity.^138,304,305^

In general, the OX–ZEO process is operated at high temperatures (300–400 °C) and pressures (10–100 bar),^192,195,307–311^ achieving selectivities to C_2_–C_4_ olefins between 63% and 87% within the hydrocarbon products (excluding CO_2_)^298,301^ at 10–85% CO conversion.^298,301,308,309,311^ These very high selectivities are well beyond the maximum predicted for a single conversion process based on the Anderson–Schulz–Flory distribution of the C_2_–C_4_ fraction (sum of olefins and paraffins) of 58%,^312^ such as the Fischer–Tropsch synthesis. This is a clear example of how using a bifunctional catalyst can improve the selectivity towards a certain product by catalyst design, as is discussed in more detail in the following paragraphs. However, it was shown that with increasing CO conversion the selectivity towards short olefins decreases and it remains a challenge to combine high selectivity with high conversion/activity, albeit that progress in that direction has been made.^308,309,311^ The group of Prof. Bao demonstrated high CO conversion of 85%, while maintaining 83% selectivity to short olefins and reduced CO_2_ selectivity of 32%.^311^

The understanding of the underlaying mechanism in OX–ZEO catalysis is still incomplete. Oxygenate intermediates are key in this process, but there is still discussion about which species is the main intermediate diffusing from the metal oxide to the acid catalyst.^313,314^ The mechanism of the primary conversion of CO to these reactive oxygenate intermediate species on metal oxides was studied with *in situ* near ambient pressure X-ray photoelectron spectroscopy (NAP-XPS) and infrared (IR) spectroscopy.^306^ It was found that upon reduction and exposure to synthesis gas, 36% of the surface lattice oxygen was removed from a manganese oxide (MnO_2_) catalyst, resulting in a high density of oxygen vacancies near the surface. It was proposed that CO is dissociated on these vacancies, after which oxygen is transferred to another CO molecule, forming carbonate species from which CO_2_ is desorbed. The remaining carbon atom is hydrogenated to a CH_2_ species, followed by insertion of CO and desorption of ethenone (C_2_-ketene, Fig. 9-B). Ketenes are highly reactive molecules. The chain propagation (C–C coupling) in the zeolite is reported to follow a direct associative pathway; ketene adsorbs on the acid site of the zeolite and the CH_2_ group is transferred to an olefin in a consecutive step, leaving a CO molecule behind.^315^

However, ketene intermediates are thermodynamically unstable and therefore it might be argued that methanol and/or dimethyl ether are the actual reactive oxygenate intermediate.^316,317^ The group of Prof. He proposed methanol as the intermediate from quasi-CO_2_ hydrogenation using indium–zirconium oxide and SAPO-34 zeolite, based on DFT calculations.^307^ It was found that adsorbed CO formed an O–C–O species (quasi-CO_2_) on the catalyst surface with lattice oxygen, which was then hydrogenated forming the reactive intermediate. Furthermore, the pathway of side products formation such as methane and paraffins was investigated. The formation of methane was caused by hydrogenation of methanol (or surface O–C–O species) on the metal oxide catalysts. The C_2+_ paraffins were formed after methanol traveled from the metal oxide catalyst to the zeolite forming olefins, which were then hydrogenated to paraffins in a consecutive step on the metal oxide.

The nature of the reactive oxygenate intermediate in the OX–ZEO process is hence topic of ongoing debate. Using a zinc–chromium oxide catalyst mixed with a SAPO-34 zeolite, the group of Prof. Bao identified ketene as intermediate with synchrotron vacuum ultra-violet photoionization mass spectrometry (SVUV-PIMS).^192^ To provide more evidence, ketene was flowed over modified mordenite zeolite with only either 8-membered ring (MR) pores or 12-MR pores accessible.^297^ The product spectrum was similar to the spectrum obtained from experiments converting synthesis gas using a bifunctional catalyst consisting of zinc–chromium oxide and modified MOR zeolite (Fig. 9-C).

On the other hand, methanol and dimethyl ether were identified as intermediates in the OX–ZEO process using zinc-doped zirconia catalysts mixed with SSZ-13 zeolites with various degrees of sodium ion exchange to control the density of Brønsted acid sites.^301^ The mixture of zinc-doped zirconia with fully sodium exchanged SSZ-13 in the nano scale (∼250 nm), hence without Brønsted acid sites present, showed a low CO conversion of 5% and selectivities to methanol and dimethyl ether of 65%. The CO conversion and selectivity to C_2_–C_4_ olefins increased with increasing density of Brønsted acid sites, because the intermediates could be removed from the equilibrium and converted to short olefins. The influence of strength and concentration of acid sites of a ZnAlO_*x*_/CHA OX–ZEO catalyst on the conversion of synthesis gas to olefins was investigated.^318^ With increasing Si/Al ratio of the CHA zeolite from Si/Al = 20 to Si/Al = 308 the selectivity to paraffins decreased (from 32% to 6%, CO_2_ free), while olefin selectivity increased (from 59% to 85%, CO_2_ free). This was attributed to the low acid site density in combination with decreased acid site strength by the addition of boron during zeolite synthesis. Additionally, it was found that a high density and strength of acid sites in a ZnCrO_*x*_/SAPO-35 zeolite accelerated the catalyst deactivation by enhanced coke formation and can cause increased paraffin selectivity in large zeolite crystals.^319–321^

Alternatively to the OX–ZEO process, hydrocarbon intermediates can be used to convert synthesis gas to short olefins by combining an iron (carbide) based Fischer–Tropsch core catalyst with a SAPO-34 zeolite shell.^322^ Operating at temperatures of 325 °C, the iron FTS catalyst formed typical heavy hydrocarbon products, that were cracked on the acid sites of the SAPO-34 zeolite forming C_2_–C_4_ olefins with 53% selectivity within the hydrocarbons at 55% CO conversion. Remarkably, the CO_2_ selectivity was only 17%, which allows this approach to compete with the OX–ZEO process, although the olefin fraction in the hydrocarbon products is lower. Similar trends have been observed using a silicalite-1 encapsuled iron-based catalyst.^323,324^ Additionally, an iron-based FTO catalyst capsuled with an H-ZSM-5 zeolite or a hydrophobic SiO_2_ shell showed reduced CO_2_ selectivity (8.5–28%) and slightly increased C_2_–C_4_ olefins selectivity (41%–49%) compared to the FTO catalyst alone (30–39% CO_2_ and 25–38% olefins).^325,326^ A silica-coating of a manganese promoted cobalt carbide nano-prism FTO catalyst showed increased olefins selectivity (from 40% to 59%) and reduced CO_2_ selectivity (from 45% to 15%) compared to the uncoated catalyst.^327^ It was concluded that the silica-coating reduced the adsorption of water on the catalyst and promoted the diffusion of water away from the catalysts' active sites, thereby, lowering the actual concentration near the active FTO sites.^327,328^

In the Fischer–Tropsch to olefins process, CO activation and C–C–Coupling take place on the same catalyst component, while they are spatially separated in the OX–ZEO process (Fig. 9-C). The OX–ZEO process is operated at rather high temperatures, which shifts the equilibrium between synthesis gas and the reactive intermediates far to the side of synthesis gas (see section 3.2). This can partially be counteracted by operating at elevated pressures. However, it is essential to have the two functions for CO activation and C–C–Coupling in optimal proximity to effectively achieve removal of the intermediates, and hence high conversion.

The group of Prof. Wang investigated the influence of the distance between the two functions on the conversion and selectivity in the OX–ZEO process, by combining a zirconium–zinc binary oxide catalyst with a SAPO-34 zeolite in different mixing modes.^298^ Packing in stacked bed mode with the zeolite downstream of the metal oxide gave very low conversions. A physical mixture of catalyst granules with grain size of 250–600 μm (resulting distance between CO activation and C–C coupling catalyst in the range of ∼500 μm) led to 7% CO conversion with 75% selectivity to C_2_–C_4_ olefins. To achieve even closer proximity, the two individual catalysts were ground in a mortar, resulting in ∼500 nm distance between the two functions. The distance of the two functions could be decreased even further to ∼100 nm by a ball-milling procedure for 24 h. Bifunctional catalysts prepared with closer proximity using mortar-mixing and ball-milling techniques achieved CO conversions of 10–11%, with selectivities to C_2_–C_4_ olefins slightly decreasing to 63–70%. The decrease in selectivity was assigned to secondary hydrogenation of olefins to paraffins on the metal oxide catalyst. A ZnCrO_*x*_/SAPO-34 catalyst applied in the OX–ZEO to olefins reaction showed the best performance of 60% CO conversion and 76% C_2_–C_4_ olefin selectivity at medium proximity of 200–300 μm between the metal oxide and zeolite function.^329^ The authors concluded that with greater distance between the functions the removal of reactive intermediates suffered from mass transfer limitation. However, with increasing proximity zinc species migrated from the metal oxide to the zeolite, decreasing the activity of both functions. A MnO_*x*_/SAPO-34 catalyst did not show decreasing activity with increasing proximity.

The catalysts used in the OX–ZEO process are also active for the water-gas-shift (WGS) reaction and usually show CO_2_ selectivities between 32–45%, which is close to the equilibrium concentration of 45–49%, depending on the reaction conditions.^192,301,306,330^ This makes it possible to also convert hydrogen-lean synthesis gas obtained from coal or biomass, because one molecule of hydrogen is formed for every molecule CO that is converted. This comes at the expense of carbon atom economy because CO_2_ is being formed from CO. However, a high hydrogen partial pressure can also facilitate the secondary hydrogenation of olefins products over In_2_O_3_–ZrO_2_/SAPO-34 OX–ZEO catalysts.^331^ Hence, feeding hydrogen lean synthesis gas to the OX–ZEO process can circumvent unwanted side reactions and the WGS reaction can provide additional hydrogen further onwards in the catalyst bed.

The OX–ZEO catalysts can also be applied for the synthesis of short olefins from CO_2_ hydrogenation.^83,321,331–337^ Studies on a Mn_2_O_3_–ZnO/SAPO-34 catalyst showed that the alkaline character and CO_2_ activation over oxygen vacancies of the metal oxide function favor a high activity in CO_2_ conversion.^338^ By ball-milling the components together, hence decreasing the average distance between the two catalyst components, the performance of the OX–ZEO catalyst was further enhanced. The conversion of CO_2_ and selectivity to C_2_–C_4_ olefins increased from 20% to 30% and 49% to 80% (CO free), respectively, compared to a dual bed configuration. Additionally, the CO selectivity resulting from reverse WGS decreased from 90% to 55% upon ball-milling the catalyst.

### 3.2. Benefits

**Fig. 10:**
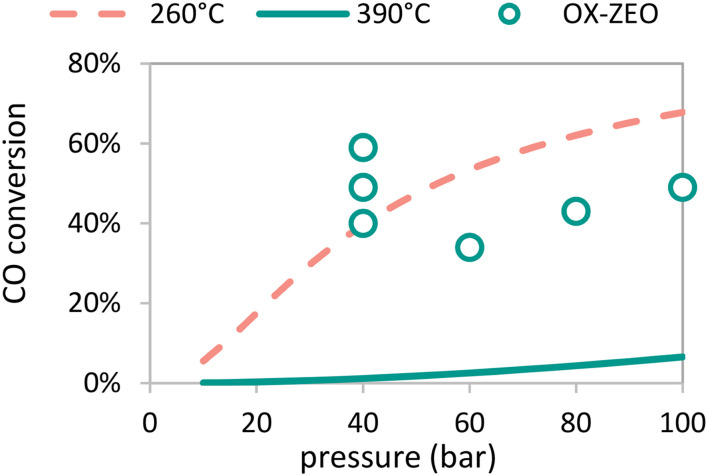
Equilibrium CO conversion for methanol synthesis from synthesis gas (H_2_ : CO = 2 mol mol^−1^) as function of reaction pressure at 260 °C (dotted red line) and 390 °C (solid green line) and reported CO conversion as a function of reaction pressure of the OX–ZEO process operated at 390 °C (open green circles) exceeding the equilibrium CO conversion of the single pass methanol synthesis at 400 °C.^308^

A clear advantage of using bifunctional catalysis to convert synthesis gas to olefins is that CO conversions can be higher than for two separate reactors for which the maximum conversion is dictated by the equilibrium between the synthesis gas and oxygenates (Fig. 10). The thermodynamic limit for CO hydrogenation to methanol is only 0.07% (at 10 bar pressure) and 6.6% (at 100 bar pressure) at 390 °C, but recently 7.5-fold to 51-fold higher conversions were reported, reaching up to 59% conversion.^308^ These CO conversion levels are similar to those for methanol synthesis commonly operated at 260 °C.^271,308^

Another clear advantage of operating the process with two different catalytic functions, whether in a single or double reactor, is the possibility to steer the selectivity. The OX–ZEO process showed a selectivity towards short olefins of up to 87% (ref. 301) caused by the highly selective MTO catalysts used for the C–C coupling step. In comparison, methanol or dimethyl ether feedstock in the DMTO (dimethyl ether or methanol to olefins) process also give 84–87% olefin selectivity.^339,340^ For Fischer Tropsch Synthesis only, the maximum selectivity according to the ASF distribution is limited to 58% C_2_–C_4_ olefins + paraffins^312^), while the FTO process, being able to break the ASF distribution, reaches 61% C_2_–C_4_ olefins.^113^

Furthermore, the product spectrum can be tuned by the choice or modifications of the C–C coupling catalyst.^341,342^ The zeolite pore size is critical. SAPO-34 or SSZ-13 zeolites as C–C coupling catalyst typically give a product spectrum with 13–20% C_2_, 40–59% C_3_ and 14–23% C_4_ products,^192,309^ which is similar to the product distribution in MTO.^139,149^ A modified MOR zeolite with selectively deactivated 12-MR pores and only 8-MR pores accessible as C–C coupling catalyst next to a ZnCrO_*x*_ catalyst for CO activation showed a remarkably high selectivity to ethene of 73%.^297^

A last potential advantage is related to the stability of the catalyst. For the MTO process, the SAPO-34 or SSZ-13 catalyst lifetime is only a few hours due to severe coke formation.^138^ The OX–ZEO catalyst was reported to display much longer lifetimes, beyond 500 h.^308^ However, no clear explanation for the high stability has been offered so far. A possibility is that the productivity of OX–ZEO is lower (∼0.3 kg_olefins_ kg_catalyst_^−1^ h^−1^ (ref. 308)) compared to the MTO process (∼2 kg_olefins_ kg_catalyst_^−1^ h^−1^ for micro- and pilot-scale and ∼5 kg_olefins_ kg_catalyst_^−1^ h^−1^ for demo- and commercial scale^304^) which for OX–ZEO will give rise to much longer catalyst life times expressed in hours. Cheng *et al.* observed a high stability of their zinc-doped zirconia catalyst (CO activation) and H-ZSM-5 zeolite (C–C coupling and aromatization) over the course of 1000 h in the conversion of synthesis gas to aromatics.^343^ They postulated that the high silicon to aluminum ratio of the zeolite played a crucial role in its stability. Furthermore, the low partial pressure of methanol/dimethyl ether or ketene suppressed the excessive alkylation of aromatic species in the dual cycle mechanism that would eventually result in the formation of polycyclic aromatics hydrocarbons and hence catalyst deactivation.^344^ This low partial pressure of intermediates also allows to operate the OX–ZEO process with a zeolite that has a high silicon to aluminum ratio and hence low acid site density, which is beneficial for the zeolite stability.^138^ The reaction conditions of the OX–ZEO process with high temperatures and high hydrogen concentrations compared to the MTO process limit the formation of soft coke,^344^ which also mitigates catalyst deactivation.

### 3.3. Challenges

**Fig. 11:**
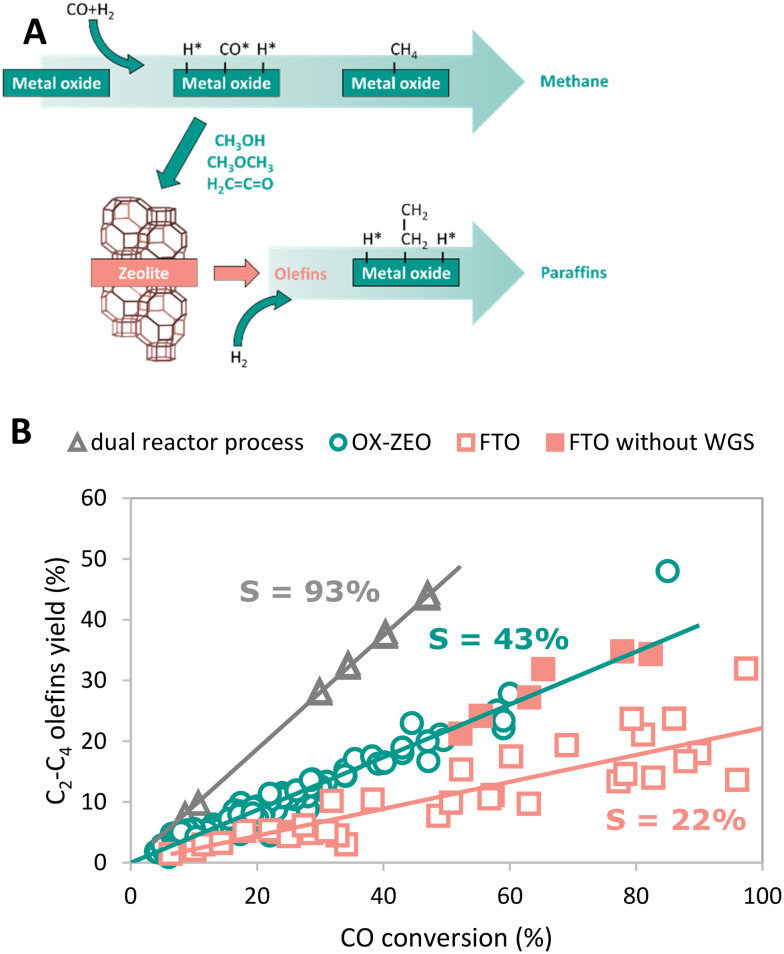
A: Proposed pathway for primary overhydrogenation forming methane on the metal oxide catalyst and secondary hydrogenation forming paraffins from olefins by re-adsorption of olefins on the metal oxide catalyst.^195^ B: Experimentally reported yields of C_2_–C_4_ olefins as function of CO conversion for a dual reactor process (single pass conversion over methanol catalyst and consecutive MTO process, gray triangles), OX–ZEO process (green circles) and FTO process (red squares, open symbols: fully WGS active, filled symbols: reduced WGS activity). The slopes of the fitted lines correspond to the overall selectivity to olefins. A detailed analysis of the catalytic data can be found in the ESI.†

A first challenge is to realize an optimum hydrogenation activity of the metal oxide in the OX–ZEO catalyst, which is crucial for the selectivity.^307,345^ Metal oxide catalysts with high hydrogenation activity, such as zinc oxide, showed primary overhydrogenation of surface carbon species forming methane (Fig. 11-A) as well as secondary hydrogenation of re-adsorbed olefins (that were formed on the C–C coupling catalyst), which is detrimental to the olefin selectivity.^195^ A Mg-HZSM-5/Al_2_O_3_ catalyst applied in the DMTO reaction in the presence of synthesis gas did not show increased hydrogenation of olefins.^346^ Hence the metal oxides' hydrogenation activity of the OX–ZEO catalyst needs to be limited, but still sufficiently high for surface CO* species to undergo moderate hydrogenation to form the reactive oxygenate intermediates.^309^ The hydrogenation performance of the metal oxide catalysts can be influenced by a variety of parameters, such as the nature of the oxide,^195^ particle size,^330,333^ nature of dopants,^347^ promoters^309^ and proximity to the zeolite.^348^ Balancing the hydrogenation activity of the metal oxide catalyst without compromising the overall catalytic performance of the OX–ZEO process remains one of the main challenges for future work. Currently, zinc–chromium or zinc–zirconium binary oxide seem to be the most promising candidates as CO activation catalysts, whereas SAPO-34, SSZ-13, and ion exchanged AlPO-18 are promising solid acids.^301,308,311^

### 3.4. Process comparison

To generate a more global picture regarding attainable product selectivities, we compared the best reported catalytic performances for three different approaches to convert synthesis gas to short olefins. Fig. 11-B shows the yield to C_2_–C_4_ olefins for the FTO process, OX–ZEO and a dual reactor process as a function of the CO conversion. For the dual reactor process, we calculated the overall yields to C_2_–C_4_ olefins that we obtained from the combination of reported data for a methanol synthesis reactor and a reactor for the methanol-to-olefins (MTO) process. The dual reactor approach is based on two consecutive reactors, separating the CO activation catalyst (methanol synthesis) from the C–C coupling catalyst (MTO). The slopes of the yields plotted against the CO conversion per pass (once-through) correspond to the overall selectivity of the process and account for the formation of CO_2_.

The FTO process shows a selectivity to C_2_–C_4_ olefins of ∼22%. The fraction of short olefins in the hydrocarbon products of the FTO process is reported up to 61%.^349^ However, the product stream is not only hydrocarbons, but also contains CO_2_ formed from CO *via* the WGS reaction, giving 30–50% CO_2_ in the product stream.^119,350^ This reduces the overall selectivity to short olefins to 30–42%. A successful strategy in the case of hydrogen-rich synthesis gas might be to mitigate the WGS reaction, as illustrated by the full red square in Fig. 11-B, which shows the yield of short olefins for a bifunctional catalyst consisting of an iron (carbide) based core and a SAPO-34 or silica shell, respectively.^322,326^ The increased selectivity is caused by the reduced WGS activity of this catalyst system and can compete with that in the OX–ZEO process. A selectivity to short olefins of ∼43% can be observed for the OX–ZEO process, caused by a high contribution of short olefins to the hydrocarbon products as high as 87% (ref. 298 and 301) in combination with high CO_2_ selectivities of 40–45% due to the WGS activity of the OX–ZEO catalysts.^192,307^

The approach with two separate reactors (methanol synthesis and MTO) gives an overall selectivity of ∼93%. This is caused by the high selectivity of the methanol synthesis (between 97% and 99.8%^351,352^) and the MTO process with 94–96% selectivity to short olefins.^138^ Furthermore, in this configuration the total selectivity towards CO_2_ from WGS (reaction of water and CO) is neglectable, because the water is mainly formed during the MTO process in the second reactor without CO being present.

Currently, a dual reactor approach to convert synthesis gas to olefins using methanol synthesis and an MTO process shows the most promising overall selectivity and carbon atom economy due to the absence of the WGS reactions and highly selective reactions. A challenge for the OX–ZEO process as well as for the FTO process is the suppression of the WGS reaction if hydrogen-rich synthesis gas is used to achieve higher yields of the desired products. This has been partially achieved with a zinc–cerium–zirconium oxide catalyst combined with a SAPO-34 zeolite.^353^ The selectivity towards carbon dioxide was reduced to 6% at a low CO conversion of 7%. However, the CO_2_ selectivity increased to 26% at 12% CO conversion by increasing reaction temperature. In terms of activity the hydrogenation strength of the metal oxide needs to be finely balanced to achieve higher total activity without intensifying secondary hydrogenation of products. For this, several strategies are already available, such as choice of material,^354^ dopants and promoters,^355^ or intimacy between the metal oxide and zeolite.^356^ Concerning catalysts stability important progress has been achieved with stable times on stream of 500 h and above.^308,343^ In brief, the two-step approach cannot compete with the two-reactor approach in terms of selectivity and carbon yield but does have clear advantages, such as exceeding conversion levels of the methanol synthesis catalysts dictated by the thermodynamic limits. Additionally, as it is a relatively new method, further development can be expected.

## 4. Aromatics

### 4.1. Recent developments

**Fig. 12:**
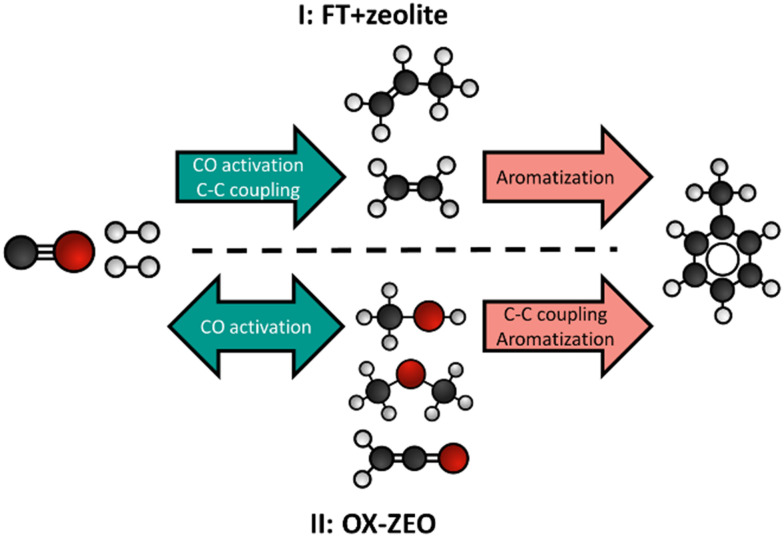
General reaction scheme for the conversion of synthesis gas to aromatics using a combination of an FT catalyst with a zeolite (I) or the OX–ZEO process (II).

In the following paragraphs we introduce two different approaches to convert synthesis gas to aromatics (monoaromatics with a single aromatic ring) using bifunctional catalysis: (I) the combination of an FT catalyst with a zeolite and (II) the OX–ZEO process for aromatics (Fig. 12). One of the main differences between these two approaches is the location of the C–C coupling. In the combination of the FT catalyst and the zeolite (FT + zeolite) the C–C coupling takes place on the CO activation catalyst (the FT catalyst),^357^ while the zeolite is responsible for further oligomerization, cyclization and aromatization. In the OX–ZEO process the CO activation catalyst (metal oxide) forms carbon monomers,^343,358^ while the C–C coupling of these carbon monomers occurs on the zeolite.^347^ In both approaches, H-ZSM-5 zeolites with 10 membered ring pores are typically used, due to their excellent shape selectivity for aromatics.^359^

#### Combination of FT catalyst and zeolite

Iron carbide- or cobalt carbide-based FT catalysts show high selectivities to olefins^119,121,360^ and can be combined with zeolites to convert these olefin intermediates to aromatics.^357,361,362^ FT + zeolite is commonly operated at moderate temperatures (270–320 °C) and medium pressures (10–20 bar),^357,363,364^ reaching aromatics selectivities up to 61% in the hydrocarbon products (excluding CO_2_, 9–41% if CO_2_ is accounted for) at 5–99% CO conversion.^357,364,365^ The FT product spectrum usually follows the Anderson–Schulz–Flory (ASF) distribution. A wide range of products is formed, including methane and longer paraffins, that cannot be converted to aromatics under these reaction conditions.^366^ This explains the moderate overall selectivity to aromatics. Decreasing the reaction temperature causes an increase of average chain length of the FT products and leads to a higher fraction of olefin intermediates with a chain length of C_6_–C_10_, hence suitable for aromatization.

For the aromatization of olefins over H-ZSM-5 higher temperatures (350–480 °C) and lower pressures (1 bar–10 bar) are preferred.^358,367–369^ However, operating an FT catalyst under these conditions leads to rapid deactivation due to coke formation.^113,121^ Furthermore, more methane and less C_2+_ are formed.^370^ For example, a bifunctional catalyst consisting of a cobalt–manganese–aluminum oxide catalyst combined with an H-ZSM-5 zeolite operated at 270 °C and 10 bar led to 4% methane formation and 2–5% aromatics, while at 320 °C a 18–21% methane selectivity and 29–38% aromatics were formed. Alternatively, a tandem reactor design with the same cobalt–manganese–aluminum oxide catalyst upstream at 270 °C and the zeolite downstream at 320 °C allowed to maintain a low methane selectivity of 3% while increasing the selectivity to aromatics to 52%,^357^ albeit at the expense of CO conversion (32–40% at 270 °C compared to 65–72% at 320 °C).

**Fig. 13:**
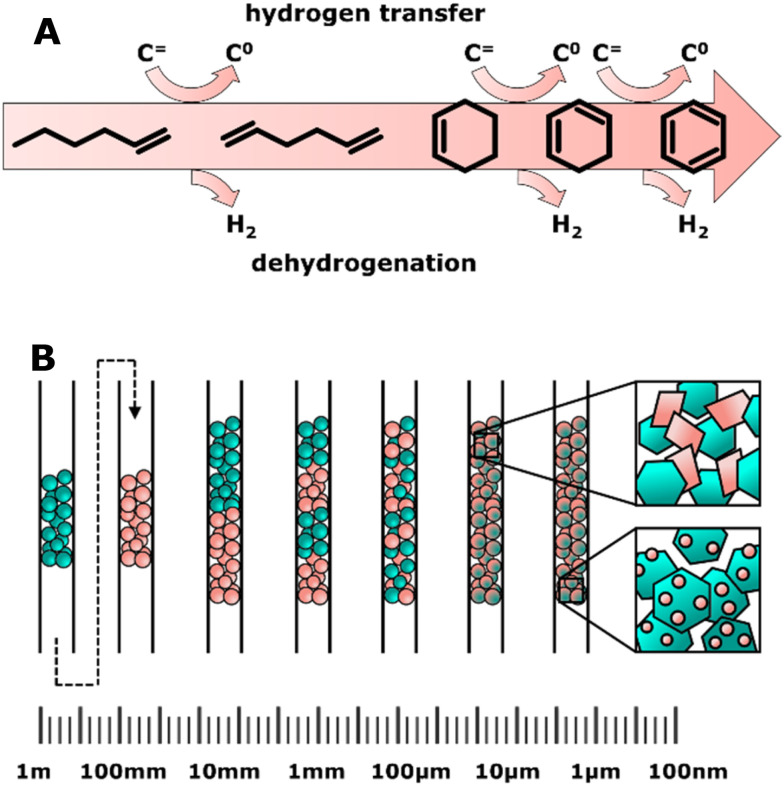
A: Aromatization of higher olefins *via* hydrogen transfer^134^ (top), forming paraffins (C^0^) from olefins (C^=^) and *via* dehydrogenation^372^ (bottom) releasing hydrogen as by-product. B: Overview of different bifunctional catalyst configurations showing distances between the two catalytic functions varying from the meter scale in dual reactor processes to the nano mater scale for closest proximity.

The aromatization of olefins often follows a pathway that involves hydrogen transfer, forming three molecules of paraffins for every aromatic molecule formed (Fig. 13-A).^358,371,372^ However, a high operating temperature and/or low partial pressure of olefins shifts the aromatization towards dehydrogenation instead of hydrogen transfer.^372^ For example, operating a bifunctional catalyst consisting of a Fischer–Tropsch to olefins (FTO) catalyst and an H-ZSM-5 zeolite at 400 °C, 1 bar and low CO conversion (2%) facilitates aromatization of olefins *via* a dehydrogenation pathway with 17% aromatics selectivity and only 4% paraffins.^113^ Alternatively, a bifunctional catalyst consisting of a pyrolyzed iron containing metal–organic-framework (MOF) promoted with sodium and a modified H-ZSM-5 zeolite has been developed for the conversion of CO_2_ and hydrogen to aromatics.^373^ Catalytic tests performed at 320 °C, 30 bar and CO_2_ containing synthesis gas (H_2_ : CO_2_ = 2.95 v/v) as feedstock and in granule stacking mode yielded 9.6% C_2_–C_4_ paraffins and 50.2% aromatics. The authors concluded that the olefin intermediates are converted into aromatics *via* dehydrogenative aromatization and that adsorbed CO_2_ on the pyrolyzed iron containing MOF acted as acceptor for hydrogen species formed during dehydrogenation.^373^

Both structure of the zeolite and acid site density have a major influence on the resulting product spectrum. For hierarchical ZSM-5 zeolites impregnated with iron, a correlation between the concentration of acid sites and the selectivity to aromatics was reported.^374^ Bifunctional catalysts with medium concentrations of acid sites (746 μmol g^−1^) gave a higher selectivity to aromatics (15%) then with a lower or higher acid site concentration. Additionally, increasing the fraction of mesopore volume within the total pore volume from 45% to 68% increased the selectivity to aromatics from 15% to 23%.^374^

A composite catalyst consisting of a copper-promoted bulk iron catalyst and an H-ZSM-5 zeolite was applied in the synthesis of aromatics from CO_2_ containing synthesis gas with H_2_ : CO_2_ = 3 (v/v).^375^ At 320 °C, 30 bar, and the individual catalyst granules being mixed in the catalyst bed showed 57% conversion of CO_2_ and 57% selectivity to aromatics with only 3.5% CO selectivity resulting from the rWGS reaction. Increasing the content of copper promoter in the bulk iron catalyst from 6.25 wt% to 50 wt% caused the methane and short paraffin selectivity to increase (11% to 57% and 8% to 30%, respectively) and the aromatics selectivity to decrease (57% to 10%). This behavior indicates that the increasing copper content is responsible for secondary hydrogenation and methane formation of intermediate species. A similar behavior was also observed when promoting iron-based FT catalysts with copper in the conversion of CO and H_2_ to aromatics.^376^ With a copper content of 1.5%wt in the iron-based FT catalyst the methane selectivity was low (8%) and aromatics selectivity was high (42.5%). However, with lower (0.2% wt) or higher (5% wt) amounts of copper promoter the methane selectivity was higher (13% and 15.5%, respectively) and aromatics selectivity was lower (37% and 35% respectively).

#### OX–ZEO

Analog to the OX–ZEO process to convert synthesis gas to olefins, the OX–ZEO process to form aromatics can be regarded as an important development. The catalyst system consists of metal oxides (zirconium, zinc and chromium-based) and zeolites.^377–380^ CO activation takes place on the metal oxide and leads to reactive intermediates, such as methanol, dimethyl ether and/or ketenes,^363,381–383^ which can be considered as carbon monomers. These intermediates are further converted into aromatics *via* C–C coupling over a zeolite. Mostly zeolites with 10-membered ring pores are used, such as H-ZSM-5 and H-ZSM-11, which combine an excellent pore structure for the synthesis of aromatics with strong Lewis and Brønsted acid sites.^303,343,381^

The OX–ZEO process to convert synthesis gas to aromatics is usually operated at high temperatures (300–450 °C) and high pressures (20–60 bar),^343,354,363,379^ reaching CO conversions between 3% and 55% and selectivities to aromatics of 49–86% within the hydrocarbon products (29–67% selectivity to aromatics if the CO_2_ formation is taken into account),^363,379,383^ which is higher than FT + zeolite. The high temperature of the OX–ZEO process leads to a low equilibrium concentration of intermediates, which steers the aromatization towards a pathway that involves dehydrogenation rather than hydrogen transfer.^372^ Therefore, the formation of paraffins is limited and a high selectivity to aromatics can be achieved. However, the competition between aromatization *via* hydrogen transfer and *via* dehydrogenation strongly depends on the composition of the synthesis gas.^363^ An H_2_ : CO ratio of 2 (v/v) showed a significantly higher C_2_–C_4_ paraffin fraction of 53.1% and low aromatic fraction of 35.2% in the hydrocarbons, compared to a hydrogen lean feed gas with H_2_ : CO = 1 v/v, resulting in 20.1% C_2_–C_4_ paraffins and 56.3% aromatics in the hydrocarbon products.

The nature of the reactive intermediate of the OX–ZEO process to convert synthesis gas to aromatics is under debate. Using zinc chromium oxide, zinc manganese oxide, zinc–zirconium oxide, or zinc alumina catalysts for CO activation, a mixture of methanol and dimethyl ether was found as reactive intermediate.^343,379,381,384,385^ By feeding methanol and carbon monoxide over a zinc zirconium oxide catalyst mixed with an H-ZSM-5 zeolite it was found that the presence of CO showed a self-promoting effect in the conversion of methanol to aromatics, in which methanol was converted to short olefins on the zeolite. These olefins underwent aromatization *via* dehydrogenation, whereas the hydrogen was removed by carbon monoxide on the metal oxide catalyst resulting in the formation of methanol.^343^

Alternatively, ketene was proposed as reactive intermediate using zinc manganese oxide or zinc chromium oxide as CO activation catalyst.^378,381^ The zinc chromium oxide catalyst mixed with a mesoporous SAPO-34 zeolite already has been studied in the OX–ZEO to olefins process, where ketene was identified with synchrotron vacuum ultra-violet photoionization mass spectrometry.^192^ A cerium zirconium oxide catalyst in combination with an H-ZSM-5 zeolite showed improved oxygen vacancies on the surface of the CO activation catalyst and assisted the formation of C_2+_ oxygenates and C_6+_ olefins. This suggested a reactive intermediate other than methanol in the conversion of synthesis gas to aromatics.^363^

To remove the reactive intermediates effectively and hence increase the synthesis gas conversion, close proximity between the CO activation catalyst and the C–C coupling catalyst is crucial.^384^ With increasing proximity going from powder mixed bifunctional catalysts with ∼100 nm distance between the different catalytic functions to nanocomposites, the CO conversion and selectivity to aromatics increases, whereas the methane selectivity decreases.^343,363^ This indicates that a larger distance between the CO activation catalyst and the zeolite gives rise to secondary hydrogenation of reactive intermediates on the metal oxide catalyst, forming methane and C_2+_ paraffins. However, a reduced zeolite crystallite size from 1.5 μm to 200 nm in a physical mixture with a ZnCrO_*x*_ catalyst showed reduced selectivity to aromatics and a 1.7-fold increase of side products^386^ which was assigned to enhanced secondary hydrogenation. Additionally, the zeolites owned different morphologies.

Generally, the formation of *ortho*- and *meta*-xylene and heavier aromatics takes place at the acid sites on the external surface of the zeolite by isomerization and alkylation.^387^ The kinetic diameter of *para*-xylene is smaller than those of *ortho*- and *meta*-xylene and only *para*-xylene can be formed inside the micropores of H-ZSM-5.^379,380^ Surface modification of the zeolites forming aromatics can have a significant influence on the product distribution within the aromatics fraction (Table 1). These modifications can be achieved by passivation of the external surface of the zeolites or by the growth of a shell that is free of acid sites (for example a silicalite-1 shell around H-ZSM-5 crystals), and generally lead to higher fractions of *p*-xylene in the aromatics.

**Table 1:** Influence of surface modifications of zeolites for the formation of aromatics on the selectivity

Reaction/catalyst	Zeolite modification	Aromatics selectivity		Reference
		Without modification	With modification	
MTA	Chemical liquid deposition	24% *p*-xylene in xylenes	90% *p*-xylene in xylenes	388
H-ZSM-5				
Disproportionation of toluene	Silicalite-1 shell		80% *p*-xylene in xylenes	389
H-ZSM-5				
Fe + Z	Zn-promotion and silicalite-1 shell	20–25% *p*-xylene in xylenes	65–70% *p*-xylene in xylenes	390
Mn-promoted FT catalyst + H-ZSM-5				
OX–ZEO	Zn-promotion and silicalite-1 shell		77% *p*-xylene in xylenes	379
CrZnO_*x*_ + H-ZSM-5				
OX–ZEO	USY zeolite downstream of OX–ZEO catalyst	63% BTX in aromatics	88% BTX in aromatics	391
SiO_2_-modified MnCrO_*x*_ + ZSM-5				

OX–ZEO catalysts often show high CO_2_ selectivities due to their strong WGS activity. However, the group of Professor Tsubaki developed a catalyst that allows to convert CO_2_ containing synthesis gas (CO : CO_2_ : H2 = 6.1 : 1 : 12.8 v/v/v) into aromatics.^392^ The rate of CO_2_ formation and consumption was kept in balance by adjusting the feed composition, hence this reaction was operated net-CO_2_ neutral. The OX–ZEO catalyst consisting of Cr_2_O_3_ as metal oxide and H-ZSM-5, or metal ion exchanged ZSM-5 zeolite with a silica coating showed Co_*x*_ conversion between 17.4% and 24.6% (CO conversion: 18.3–28.4% and CO_2_ conversion: 1.5–13.1%, respectively) and aromatics selectivity between 65% and 76%. It is worth mentioning that Cr_2_O_3_ combined with a gallium exchanged and silica coated ZSM-5 zeolite performed with the highest Co_x_ conversion (24.6% Co_*x*_, 28.4% CO, 1.5% CO_2_) and simultaneously with the highest selectivity to aromatics (76.4%) of the tested bifunctional catalysts. Additionally, the silica coating of the ZSM-5 zeolite reduced the alkylation of aromatics on the external acid sites of the zeolite, hence increasing the C_6_–C_8_ aromatics selectivity to 55%. *p*-Xylene was formed with 38.6% selectivity.

Analog to the OX–ZEO to olefins reaction, the production of aromatics can also be achieved by CO_2_ hydrogenation and reach CO_2_ conversion levels between 9% and 41% and selectivities to aromatics of up to 76%.^393^ Using a chromium-doped ZrO_2_ aerogel catalyst combined with an H-ZSM-5@SiO_2_ zeolite CO_2_ conversion of 14% was achieved with 77% aromatics selectivity, of which 2/3 were light aromatics (C_6_–C_8_).^394^ The methane selectivity was low with only 1.1%.

### 4.2. Benefits

#### Combination of FT catalyst and zeolite

The performance of the CO activation catalyst (FT catalyst) does not depend on the equilibrium between synthesis gas and the reactive intermediate. This gives more freedom in the catalyst bed and reactor design. Hence a range of different configurations is reported, from iron nano particles directly anchored on the zeolite,^395^ and mixing individual catalyst grains in the catalyst bed, to stacked bed and tandem reactor design in which the individual catalysts are spatially separated (Fig. 13-B).^357^

Interestingly, combining a sodium and sulfur promoted FTO catalyst and an H-ZSM-5 zeolite in a physical mixture caused a 1.8-fold activity enhancement of the FTO catalyst compared to the FTO catalyst without zeolite or in stacked bed mode, where the two functions are spatially separated.^113^ Although not fully understood, Mößbauer spectroscopy measurements revealed an enhanced formation of iron carbide, which is the active phase in the FTO reaction for the physical mixture of FTO catalyst and zeolite. Here, 83% of the iron was transformed into an iron carbide phase after a 1 h carburization step at 290 °C and atmospheric pressure, whereas the FTO catalyst without zeolite only showed 57% carbide formation. Hence unexpected benefits can arise from the close coupling of the two functions.^396^

#### OX–ZEO

**Fig. 14:**
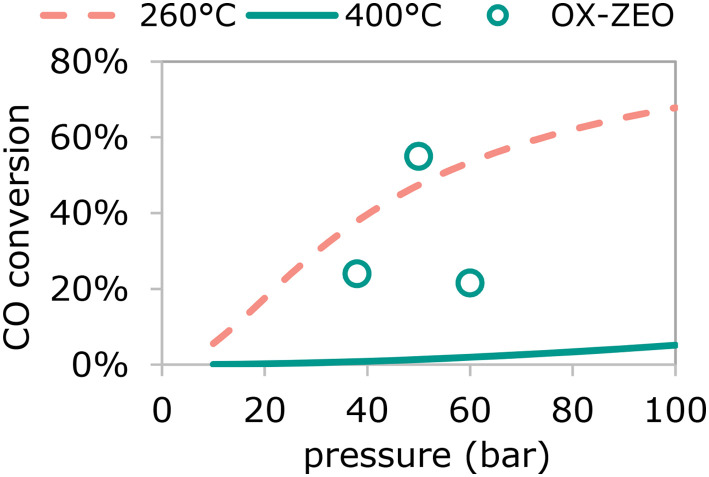
Equilibrium CO conversion for methanol synthesis from synthesis gas (H_2_ : CO = 2 mol mol^−1^) as function of reaction pressure for 260 °C (dotted red line) and 400 °C (solid green line) and reported CO conversion as function of pressure for the OX–ZEO process operated at 400 °C (open green circles) exceeding the equilibrium CO conversion of the single pass methanol synthesis at 400 °C.^354,377,379^

A clear asset of the OX–ZEO process is the enhanced conversion with the bifunctional catalyst system, which exceeds the equilibrium CO conversion in a single pass over a CO activation catalyst alone. Taking methanol as an intermediate, the CO conversion would be thermodynamically limited to 1.4% at 50 bar or 1.9% at 60 bar, when operating at 400 °C (Fig. 14). In the OX–ZEO process the intermediates are effectively removed from this equilibrium by the aromatization reaction, resulting in CO conversions as high as 55% at 50 bar or 22% at 60 bar.^354,379^ This corresponds to an 11-fold to 40-fold activity enhancement at the same temperature and allows the OX–ZEO process to operate at 400 °C with similar CO conversions as methanol synthesis typically operated at 260 °C (Fig. 14 – dotted red line).^271^

Furthermore, the OX–ZEO process has a high fraction of aromatics in the hydrocarbon products compared to FT + zeolite. The formation of aromatics from methanol follows a dual cycle mechanism in which higher olefins are formed in the alkene cycle that undergo aromatization to enter the aromatic cycle as shown in Fig. 3.^134^ The aromatization of higher olefins is based on the following steps: formation of dienes, cyclization to cyclic olefins, formation of cyclic dienes and formation of aromatics (Fig. 13-A). Commonly reported, the formation of dienes, formation of cyclic dienes and the aromatization is based on hydrogen transfer, in which also paraffins are formed at the expense of olefins. However, operating the OX–ZEO process at high temperatures and low reactive intermediate concentrations facilitates the aromatization *via* dehydrogenation, forming molecular hydrogen instead of paraffins.^372^ Therefore, the OX–ZEO shows higher selectivity than FT + zeolite, which operates at lower temperatures and higher concentrations of reactive intermediates. In FT + zeolite, the aromatization is more likely to follow hydrogen transfer and form undesirable paraffins.^357,364^ Furthermore, the product spectrum of the CO activation catalyst (FT catalyst) depends on the ASF distribution and the maximum selectivity of suitable intermediate products to be converted into aromatics is limited.^397^

The OX–ZEO process shows stabilities that exceed the stability of the methanol-to-aromatics process. The group of Prof. Wang presented an OX–ZEO catalyst system consisting of a zinc zirconium oxide catalyst and H-ZSM-5 zeolite, which showed stable performance with 80% selectivity to aromatics and CO conversion of 20% over the course of 1000 h at 400 °C and 30 bar.^343^ In the methanol-to-aromatics reaction the activity in methanol conversion drops significantly after 5–200 h, when operated at the same reaction temperature.^155,398,399^ Due to the low partial pressure of reactive intermediates, zeolites with high silicon-aluminum ratios and hence low density of strong acid sites can be used in the aromatization reaction, which is beneficial for the zeolite stability.^278,343^ Additionally, the low partial pressure of intermediates and the high reaction temperature contribute to the catalyst stability albeit at the expense of activity of the metal oxide catalyst.^372^ Another explanation for the stability expressed in hours could be the low productivity of the OX–ZEO catalysts (∼0.04 kg_aromatics_ kg_catalyst_^−1^ h^−1^) compared to the MTA process (∼0.4–1.4 kg_aromatics_ kg_catalyst_^−1^ h^−1^), which leads to a lower rate of coke formation.^155,343,398^

### 4.3. Challenges

#### Combination of FT catalyst and zeolite

A great challenge for the FT + zeolite approach is to find optimum reaction conditions. High temperatures generally give a high methane and lower olefin production in the first step, not allowing aromatization. Hence a high temperature FT catalyst with a suitable alpha value, low methane selectivity and high olefin to paraffin ratio is needed to be combined with an H-ZSM-5 zeolite in a high temperature process. Alternatively, a zeolite or another solid acid capable of converting olefins at low temperatures into aromatics needs to be identified. Dopants such as gallium or zinc can increase the performance at lower temperatures, as it was shown for the aromatization of propane.^400^ Furthermore, these dopants can lead to increased dehydrogenation activity, shifting the aromatization pathway away from hydrogen transfer towards dehydrogenation.^401,402^ However, the dehydrogenation activity of the dopants incorporated in the zeolites can also facilitate secondary hydrogenation of the olefins intermediates that are formed on the FT catalyst.

The addition of sodium and sulfur promoters to a supported iron carbide based Fischer–Tropsch to olefins (FTO) catalyst decreased the methane production from synthesis gas and gave a high selectivity towards short olefins.^403,404^ Combining this promoted FTO catalyst with an H-ZSM-5 zeolite enabled the direct synthesis of aromatics from synthesis gas.^113^ However, in close proximity of the FTO catalyst to the zeolite, higher methane selectivities (15% in stacked bed mode and 30–35% in close proximity) and lower aromatics selectivities (12% in stacked bed mode and 5% in close proximity) were observed, probably due to migration of alkaline promoters from the FTO catalyst to the zeolite, which led to neutralization of the acid sites on the zeolite.^15,405^ The migration of promoters and the accompanying effects on the catalytic performance could be circumvented by placing the zeolite downstream of the FTO catalyst in a stacked bed mode. Alternatively, by using carbon nanofibers as support material, the migration of promoters was suppressed, despite close proximity of the two catalytic functions.^15^ This shows that controlling the mobility of mobile species, such as promoters or dopants, is crucial for the design of bifunctional catalysts for this process.

#### OX–ZEO

For effective OX–ZEO catalysts, optimizing the hydrogenation activity of the metal oxide is crucial. It needs to be low enough to avoid significant secondary hydrogenation, but still provide sufficient activity to convert synthesis gas into reactive intermediates. The hydrogenation activity of the metal oxide can be controlled among others by the molar composition of mixed oxides.^343^ Using a zinc zirconium oxide catalyst in combination with an H-ZSM-5 zeolite, it was shown that zinc oxide is mainly responsible for hydrogen activation in the metal oxide catalyst. A low zinc content of Zn : Zr = 1 : 1000 mol mol^−1^ resulted in low CO conversion (14%) in combination with high selectivity to aromatics (76%), whereas the CO conversion increased (43%) and the selectivity to aromatics decreased (7%) with increasing zinc content (Zn : Zr = 1 : 5 mol mol^−1^). Furthermore, the selectivity to short paraffins increased from 18% to 52% with the same change in zinc fraction, indicating that a high zinc content enables secondary hydrogenation of reactive intermediates. Hence, a high hydrogenation activity is beneficial for the CO conversion but has a negative effect on the selectivity towards aromatics.

This hypothesis was supported by experiments performed with a cerium zirconium oxide catalyst mixed with an H-ZSM-5 zeolite, for which the selectivity towards aromatics and olefins decreased with increasing hydrogen content in the synthesis gas.^363^ Operating at 450 °C and 20 bar, the OX–ZEO catalyst showed 13.3% olefins in the range of C_2_ to C_4_ and 56.3% aromatics selectivity at a hydrogen to carbon monoxide ratio of 1 (v/v). Increasing the hydrogen content of the synthesis gas to H_2_ : CO = 2 (v/v) led to decreased selectivity to short olefins of 3.4% and aromatics of 35.2%. This shows that for the OX–ZEO catalyst the hydrogenation activity needs to be carefully optimized.

### 4.4. Process comparison

**Fig. 15:**
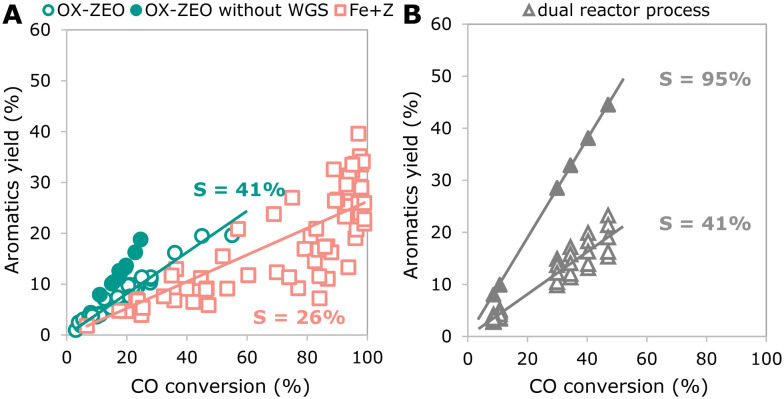
A: Reported yields of aromatic hydrocarbons as function of CO conversion for the OX–ZEO process (green circles) and combination of FT catalyst and zeolite (red squares). The filled green circles show the yield to aromatics as function of CO conversion of the OX–ZEO process with reduced WGS activity B: Calculated overall aromatic selectivity resulting from the combination of a methanol synthesis reactor and a reactor for methanol aromatization *via* hydrogen transfer (open gray triangles) and dehydrogenation (solid gray triangles) in a dual reactor process. The slopes of the fitted lines correspond to the overall selectivity of the process. A detailed analysis of the catalytic data can be found in the ESI.†

In this section we focus on the overall selectivity to aromatics. Fig. 15-A shows the best reported aromatic yields as function of the CO conversion for the OX–ZEO process and for FT + zeolite. To compare, we added the aromatic yields of a dual reactor process in Fig. 15-B, calculated from a combination of reported data for a methanol synthesis reactor^351,352^ and a reactor for the methanol-to-aromatics (MTA) process.^155,398,402,406^ The slopes of the yields plotted against the CO conversion correspond to the overall selectivity of the processes (taking the formation of CO_2_ as one of the alternative products into account).

The combination of an FT catalyst with a zeolite showed an average selectivity to aromatics of ∼26%. The results are distributed over a range of 9–41%. Li *et al.* found that in the conversion of synthesis gas to aromatics the intimacy within the bifunctional catalysts played a crucial role for the selectivity, which can explain these wide-spread selectivities.^407^ The overall selectivity is rather low which can be explained by limited selectivity to suitable olefinic intermediates in the first step. Furthermore, the temperature needed for the CO activation catalyst favors the aromatization to follow the hydrogen transfer pathway, forming three molecules of paraffins from olefins for every aromatic molecule being formed.^372^

The OX–ZEO process for aromatics showed a higher overall selectivity of 41% to aromatics, resulting from a high fraction of aromatics in the hydrocarbon products between 49% and 86% but also high selectivities to CO_2_ in the range of 17% to 49%.^363,378^ The high aromatics fraction can be explained by the low partial pressure of reactive intermediates and the high reaction temperature, shifting the aromatization towards dehydrogenation.^372^ The OX–ZEO process shows a selectivity to paraffinic hydrocarbon side products as low as 6%.^343,378^ The high CO_2_ selectivity is caused by the WGS activity of the OX–ZEO catalysts and is in the same range as for the FT + zeolite systems, showing 16–49% CO_2_ selectivity.^364^ However, tailoring an OX–ZEO catalyst to reduced WGS activity and adapted feed compositions showed that selectivities to aromatics of ∼70% are possible (Fig. 15-A).^392,408^

The calculated overall aromatic selectivity resulting from the combination of a methanol synthesis reactor and a reactor for the MTA process shows a selectivity to aromatics of ∼41% (Fig. 15-B, open triangles), which is higher than FT + zeolite and in the same range as the OX–ZEO process. We based these calculations on reported catalytic data of single pass conversions of synthesis gas over methanol synthesis catalysts with CO conversion ranging from 9% to 47% and methanol selectivities of 97–99.8%.^351,352^ Consecutively, methanol is converted into aromatics *via* suitable zeolite catalysts in a separate MTA process. A moderate selectivity to aromatics from methanol was reported between 33% and 50%, which is in good agreement with the dual cycle mechanism in combination with hydrogen transfer, in which a substantial amount of paraffins of usually ∼40% is formed.^155,398,402,406^ This decreases the overall selectivity to aromatics, despite the absence of WGS activity and therefore no significant formation of CO_2_ in this approach. However, a zinc doped H-ZSM-5 zeolite operated at high temperature of 475 °C showed a selectivity to aromatics of 96% in the conversion of methanol, due to aromatization *via* dehydrogenation.^156^ The theoretically calculated maximum overall selectivity to aromatics from the combination of methanol synthesis and aromatization *via* only dehydrogenation in a dual reactor process was ∼95% (Fig. 15-B, solid triangles).

The high CO_2_ production in both the OX–ZEO process and FT + zeolite presents a great challenge for the conversion of hydrogen-rich synthesis gas to aromatics. According to the proposed reaction mechanism for the OX–ZEO process to form olefins with ketene intermediates, the formation of CO_2_ is inevitable, since the oxygen from carbon monoxide is removed from the surface of the metal oxide catalyst *via* CO oxidation.^306^ Iron carbide-based FT catalysts with high olefin selectivity commonly show high WGS activity and CO_2_ selectivities between 21% and 50%.^350,409^ The WGS of cobalt carbide-based FT catalysts is slower and leads to CO_2_ selectivities between 2% and 13%.^360^ However, for the cobalt carbide-based catalysts the selectivity to short olefins is rather low (17–30% in the hydrocarbons). Hence, an important challenge for bifunctional catalysis is the suppression of CO_2_ formation and the combination of cobalt carbide-based FT catalysts (with increased olefin selectivity) with a zeolite seems to have the potential to achieve this.^410^

However, selectivity is not the only important factor determining the feasibility of a process. To analyze the economic feasibility of a process for the direct conversion of synthesis gas to aromatics, technical-economical aspects were simulated by Song *et al.* using ASPEN software.^411^ Here, the direct conversion of synthesis gas to aromatics was compared to a dual reactor process, whereas synthesis gas was first converted to methanol and the methanol was further converted to aromatics. The simulation did not only include the reaction unit, but also units for quenching the reaction mixture, compression, distillation, cyclic absorption separation and pressure swing absorption. The catalytic data were based on 70% CO conversion per pass over the methanol synthesis catalyst and a fraction of 70–80% aromatics in the liquid products after passing an H-ZSM-5 zeolite. It was shown that a low CO conversion resulted in a low yield of aromatics and therefore low partial pressure, which gave additional challenges in product condensation and separation. To compete with a dual reactor approach with separate methanol synthesis and aromatization at individual reaction conditions, a novel bifunctional process needs to give a minimum of 66% CO conversion per pass with an aromatic fraction of 70–80% in the liquid products and very low CO_2_ selectivities. Both approaches, the OX–ZEO process and FT + zeolite, are currently not meeting these requirements.

## 5. Liquid fuels

**Fig. 16:**
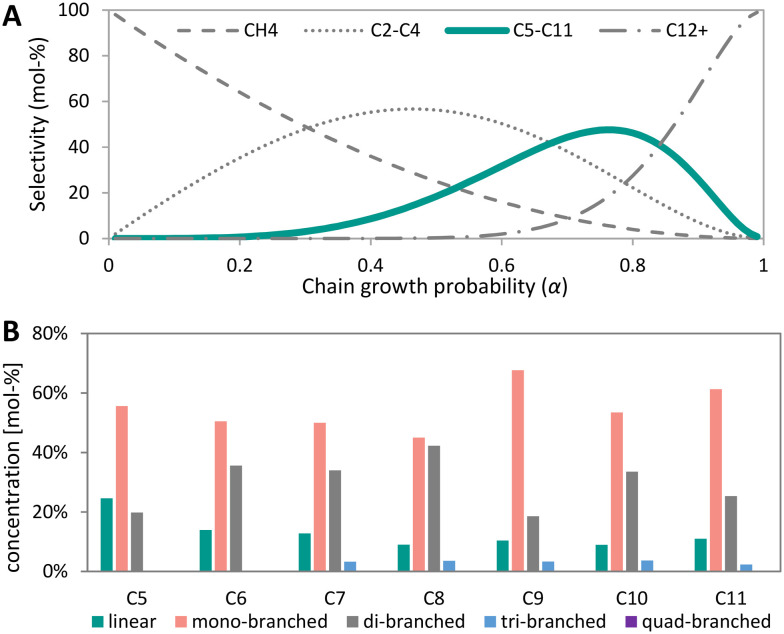
A: Anderson Schulz–Flory distribution of Fischer–Tropsch products showing a maximum selectivity to the C_5_–C_11_ fraction of 48% at chain growth probability of *α* = 0.76;^381^ B: equilibrium distribution of C_5_–C_11_ paraffin isomers at 250 °C and 20 bar. This represents the thermodynamic limitation for isomerization of *n*-paraffins (calculated with Outotec HSC 9.6.1).

The products formed in the Fischer–Tropsch synthesis are always a mixture of hydrocarbons with various chain lengths. The maximum selectivity to C_5_–C_11_ products in the Fischer–Tropsch synthesis is 48% for a chain growth probability of *α* = 0.76 according to the ASF distribution (Fig. 16-A).^381^ Increasing the production of liquid transportation fuels requires operating at higher *α*-values and cracking the resulting Fischer–Tropsch products with a too high chain length to the desired fraction.

The octane number is relevant for gasoline and strongly increases with a smaller size and a higher degree of branching of hydrocarbons.^412^ In general, the octane numbers of a hydrocarbon molecule with the same number of carbon atoms follow the trend of paraffins < olefins < aromatics (*e.g.*, *n*-hexane: 19, 1-hexene: 85 and benzene: 108).^158,413–415^Fig. 16-B shows the thermodynamic distribution of isomers according to the ASF distribution at 250 °C and 20 bar for C_5_–C_11_ paraffins, grouped by degree of branching. The maximum conversion of linear paraffins in the secondary isomerization is between 75% and 91%, leaving 9–15% *n*-paraffins un-isomerized. The main products are mono- or di-branched paraffins with 45–68% and 19–42% shares, respectively.^412^ The highly branched paraffins are key for a high-octane number, but only constitute 2–4% of the product mixture for tri-branched and 0.1–0.2% quad-branched paraffins. To convert paraffinic FT waxes into suitable gasoline fuels with octane numbers of ∼90 by the formation of aromatics, temperatures above 500 °C are applied.^416^

### 5.1. Recent developments

Different bifunctional catalysts, in particular for the direct production of gasoline from synthesis gas have been developed over the past years. These catalysts consist of iron- or cobalt-based Fischer–Tropsch catalysts, methanol or DME catalysts, or (mixed) metal oxides as the primary functional group and zeolites for the secondary conversion to gasoline. Some studies have also employed noble metals supported on zeolites, since these are industrially used as hydrocracking catalysts to upgrade paraffines by isomerization and cracking.^417^

Fischer–Tropsch catalysts can be combined with different zeolites, such as H-ZSM-5, SAPO-11, SSZ-13, mordenite, Y or beta, in order to overcome the limitations for the selectivity to C_5_–C_11_ products according to the ASF model.^418–421^ Both pore structure and acidity of these zeolites play a crucial role in the final product distribution.^418,422–424^ Acidity plays a major role for primary cracking and isomerization, whereas porosity affects the secondary olefin isomerization by micropore diffusion limitations.^425^ Larger pore sizes of zeolites facilitate the formation of multi-branched isomer products and strong acidity can cause over-cracking to lighter products.^426^ Hydrocracking catalysts such as Pt/ZSM-5 were also effective for the secondary conversion of the Fischer–Tropsch products.^417,427^

Co/SiO_2_ catalysts with different average pore diameters (10 nm and 50 nm) were used as silicon source for a Co containing zeolite catalyst with a hierarchical pore structure for the direct conversion of syngas to gasoline fuel.^41^ The resulting catalysts with the zeolite in Na-form showed high selectivities towards C_5_–C_11_ of 65–68% and 14–25% iso-paraffins in the hydrocarbon products, whereas the Co/SiO_2_ catalyst alone displayed 48–49% selectivity to C_5_–C_11_ products with 11–19% iso-paraffins. After ion-exchange to convert the zeolite into the proton form, the C_5_–C_11_ selectivity remained at the same level, but the fraction of iso-paraffins increased to 35–37%. The selectivity to C_5_–C_11_ products was further increased by the introduction of mesopores to the catalyst.^428^ Additionally, the presence of mesopores in an H-ZSM-5 support can increase the dispersion of the Co nanoparticles and hence the overall activity.^429^ Co/Al_2_O_3_ catalyst with multimodal porosity in a dual bed configuration with a Pt/nano-ZSM-5 hydrocracking catalyst showed a 2-fold increase of hydrocarbon products in the middle distillate fraction (C_10_–C_24_) compared to the configuration with a mono-modal Co/Al_2_O_3_ FT catalyst.^427^

Experiments with different average distances between Co and acid sites of an H-ZSM-5 zeolite showed a maximum selectivity to C_5_–C_11_ products for proximity in the μm-range.^430^ C_5_–C_11_ products were formed with 89.5% selectivity and 35.5% isomers in the C_5+_ products at 270 °C, 20 bar and conversion between 71% and 92%. Additionally, mesoporous H-ZSM-5 zeolite coated with a pyrolytic carbon layer prior to impregnation with Co precursor showed enhanced reducibility of the cobalt oxide, low CH_4_ selectivity and higher selectivity to C_5_–C_11_ than the catalyst system without carbon layer, due to reduced metal–support-interaction.^431^

The influence of the amount of acid sites has also been studied. Co/MCF and nano-sized H-ZSM-5, with different mass ratios in the physical mixture, showed that with increasing zeolite mass content, the selectivity to C_12+_ products decreased from 50% (Co/MCF alone) to 6% for Co/MCF : Z = 1 : 4 m/m.^432^ Also, the C_2_–C_4_ selectivity increased from 6% to 23% and the sum of iso-paraffins and olefins increased from 17% to 52% in the hydrocarbon products. The C_5_–C_11_ selectivity showed a plateau at medium zeolite content (34% for Co/MCF, 54% for Co/MCF : Z = 1 : 1 m/m, 45% for Co/MCF : Z = 1 : 4 m/m).

Iron-based Fischer–Tropsch catalysts allow to form a larger fraction of olefins in the products and are usually operated at higher temperatures compared to cobalt-based Fischer–Tropsch catalysts.^433^ The addition of a zeolite to an iron-based Fischer–Tropsch catalyst can promote the formation of aromatics, which significantly raises the octane number of the C_5_–C_11_ product fraction. A co-precipitated iron-based Fischer–Tropsch catalyst containing Cu, Mg and K as promoters showed 53% selectivity to C_5_–C_11_ products of which 4% were aromatics (300 °C, 10 bar, CO conversion 70–90%).^434^ The addition of an H-ZSM-5 with medium concentration of acid sites (Si/Al = 240) by physical mixing increased the selectivity to C_5_–C_11_ products to 67% with 73% aromatics in this fraction. A physical mixture of the iron-based Fischer–Tropsch catalyst with an H-ZSM-5 with high acid site concentration (Si/Al = 40) increased content of aromatics in the C_5_–C_11_ hydrocarbon fraction to 90%, however, the total C_5_–C_11_ fraction decreased to 58% due to over-cracking and increased formation of C_1_–C_4_ products. Additionally, the olefins/paraffin ratio increased 3–7-fold upon zeolite addition compared to the Fischer–Tropsch catalyst alone. All experiments showed high water-gas-shift activities with 40–44% CO_2_ formed.

Coating the iron-based catalyst with a hydrophobic methylated silica layer decreased the formation of CO_2._^435^ Further addition of an HZSM-5 zeolite packed below the FTS catalyst in the reactor led to a high C_5_–C_11_ selectivity (62.5% at 260 °C, 20 bar and 50% CO conversion) and low CO_2_ selectivity (14.3%). The authors showed that the diffusion of water through the hydrophobic layer was unidirectional, which led to a reduced CO_2_ formation by hampering the water-gas shift reaction on the iron-based catalyst.

An Fe/SiO_2_ core/shell catalyst was tested in the direct conversion of synthesis gas to gasoline.^436^ The silicalite-1 membrane applied onto the core catalyst served as protection as well as anchor point for the functional H-ZSM-5 membrane. This catalyst showed similar CO conversions (55–60%) and C_5_–C_11_ selectivities (49–53%, CO_2_-free) as the base core catalyst or the core catalyst in physical mixture with H-ZSM-5 at 280 °C and 10 bar. The selectivity to iso-paraffins was 30% higher for the core/shell catalyst than for the core catalyst alone and the physical mixture with hereof, which was ascribed to hydrogenation and isomerization of olefins, next to hydrocracking and isomerization of C_12+_ hydrocarbons.

Combining a zinc–manganese-oxide catalyst with different 10-membered ring zeolites revealed that the OX–ZEO process allows to form C_5_–C_11_ products with a high selectivity of up to 77%.^381^ The product spectrum of the ZnMnO_*x*_ catalyst mixed with SAPO-11 showed only 6% *n*-paraffins in the C_5_–C_11_ aliphatics as well as 16% aromatics in C_5_–C_11_. The CH_4_ selectivity was remarkably low (2.3%). Introducing mesopores into an H-ZSM-5 zeolite of a zinc–chromium-oxide containing OX–ZEO catalyst enhanced the selectivity to C_5+_ from 20% to 61%, while maintaining the low CH_4_ selectivity.^194^ Conversion of ketene, which is thought to be an intermediate in OX–ZEO catalysts, using H-SAPO-11 has been studied to elucidate the reaction mechanism to form C_5_–C_11_ hydrocarbons.^437^ The authors showed by *in situ* IR and quasi-*in situ* ssNMR spectroscopy that ketene transforms *via* either an acetic acid ketonization pathway or an acetoacetic acid decarboxylation pathway to acetone, butene, and C_5_–C_11_ hydrocarbons.

**Fig. 17:**
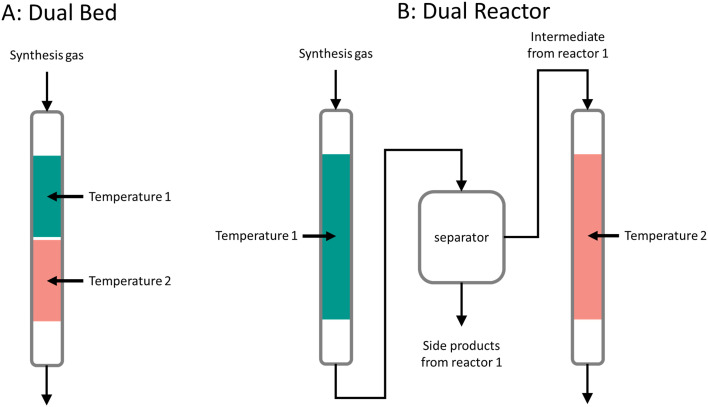
Illustration of bifunctional catalysis performed in (A) dual bed and (B) dual reactor configuration.

A DME catalyst (Cu/ZnO/Al_2_O_3_+γ-Al_2_O_3_) allows to convert synthesis gas to DME with 90% selectivity (CO_2_ free) at 300 °C, 30 bar and 65% CO conversion.^438^ In a physical mixture with a nano-sized H-ZSM-5, a DME-to-gasoline (DTG) catalyst, the CO conversion increased to 75%, C_5_–C_11_ products were formed with 26% selectivity and C_1_–C_2_ products with a selectivity of 20%. Fig. 17 illustrates the difference between the dual bed and dual reactor configurations. The dual bed configuration can have dedicated temperatures for the individual catalyst beds applied in a single reactor. The dual reactor configuration consists of two consecutive reactors with removal of intermediates between the reactors.^357,438^ Placing the two different catalysts in a dual bed configuration (Fig. 17-A) with the DME catalyst upstream led to an increase of C_5_–C_11_ selectivity to 76% and only 5% C_1_–C_2_. When the dual bed configuration was operated with dedicated temperatures for each catalyst (DME catalyst at 260 °C and DTG catalyst at 320 °C) the CO conversion increased to 87% and the C_1_–C_2_ selectivity decreased to 1.7%. C_5_–C_11_ products were formed with 79% selectivity, of which 34% were aromatics. It was also found that C_5_–C_11_ aliphatics consisted of 95% isomerized products. Increasing the temperature of the DTG catalyst bed caused the C_5_–C_11_ and aromatics selectivity to decrease. Additionally, nano-sized H-ZSM-5 DTG catalysts with medium Si/Al ratio and medium acid sites concentration show superior stability in the DME conversion to C_5_–C_11_ products, compared to zeolites with a higher acid site concentration due to reduced coke formation on the zeolite.

### 5.2. Benefits

In the following paragraphs we illustrate the potential benefits of bifunctional catalyst systems for the direct production of fuels compared to the operation in multiple individual reactors.

An OX–ZEO catalyst capable of converting synthesis gas into gasoline showed a high content of 95% of branched isomers in the C_5_–C_11_ aliphatics (non-aromatic molecules) fraction.^381^ In the OX–ZEO process branched molecules are predominantly by the alkylation of hydrocarbons with oxygenates such as MeOH, DME, or ketene.^439–441^ The dual bed process with a methanol synthesis or FTO catalyst in the first bed and zeolite at high temperature (320 °C) in the bed downstream showed a low selectivity to linear C_5_–C_11_ paraffins of 3% and 4%, respectively.^357,438^ At high temperatures, in the zeolite bed oligomerization of short iso-olefins acts as an additional source of C_5_–C_11_ branched molecules next to isomerization of linear aliphatics, which also holds for medium- and high-temperature operation of iron-based Fischer–Tropsch catalysts combined with zeolites.^397^

**Fig. 18:**
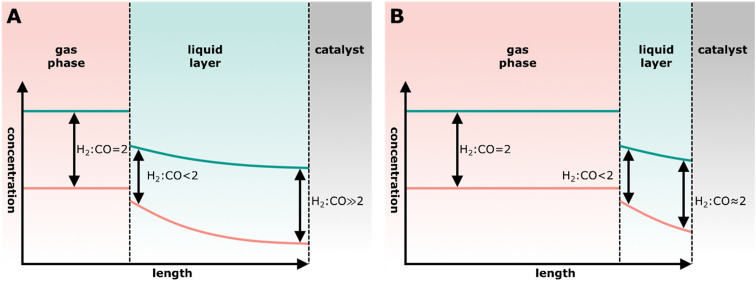
Illustration of the concentration profile of hydrogen and carbon monoxide through the gas phase and the liquid product layer to the catalyst surface with different liquid product layer thicknesses. A: A thick layer of liquid products increases the effective H_2_ : CO ratio on the catalyst surface. B: Reduction of the liquid layer thickness leads to a lower H_2_ : CO ratio on the catalyst surface.

Fischer–Tropsch catalysts can form a liquid product layer around the metal particles, inside the support's pores or in the void space between the catalyst particles. This may result in H_2_ and in particular CO profiles as a function of distance to the catalyst surface as schematically illustrated in Fig. 18.^442–449^ Hydrogen diffuses 2–3 times faster through FT wax than carbon monoxide,^450–452^ although the latter has a ∼20% higher solubility.^453–456^ As a result, the H_2_/CO ratio at the catalyst surface can be significantly higher than in the bulk of the reactor, leading to lower C_5+_ and higher CH_4_ selectivities with increasing catalyst particle size and liquid layer thickness.^457–459^ Experiments with a core/shell catalyst consisting of a Co/SiO_2_ core and H-ZSM-5 shell showed a reduced CH_4_ selectivity compared to the Co/SiO_2_ alone (10.3% *vs.* 25.7%) together with a reduced selectivity to C_11+_ (0.3% *vs.* 15.3%) at 280 °C, 10 bar and full conversion.^236^

An often claimed benefit for the conversion of synthesis gas to gasoline using bifunctional catalysis is the lower investment costs for a single reactor.^430^ However, Fischer–Tropsch reactors may have higher costs per installed unit compared to a dedicated reactor filled with a hydrocracking/isomerisation catalyst.^460^ Hence, the lower investment costs for a larger Fischer–Tropsch reactor (to accommodate the Fischer–Tropsch catalyst and the zeolite) compared to two separate reactors seems to be limited.

### 5.3. Challenges

Catalysts containing noble metals for hydrocracking are widely employed and their performances maximized. However, when employed for *in situ* hydrocracking of Fischer–Tropsch products in bifunctional catalysts, poisoning of the noble metal with carbon monoxide reduces the hydrotreating performance of these catalysts.^417,461–463^ The conversion of long chain paraffins is reduced 4.3-fold over a Pt/ZSM-5 catalyst in the presence of synthesis gas compared to H_2_ atmosphere.^417^ Olefins still undergo isomerization and cracking on the acid sites of the zeolite.

The liquid wax filling the FT catalyst pores can reduce catalyst activity by causing mass transfer limitation for synthesis gas.^442–449^ Removal or reduction of this product layer by operating alternatingly at Fischer–Tropsch and hydrogenolysis conditions can lead to enhanced activity and stability of the Fischer–Tropsch catalysts.^464^ However, a combination of Fischer–Tropsch catalyst with a zeolite to reduce the product layer by cracking did not show significant activity enhancement and displayed a similar turn-over-frequency as the FT catalyst alone,^422,429,465,466^ despite an altered ASF distribution.^431^

### 5.4. Process comparison

**Fig. 19:**
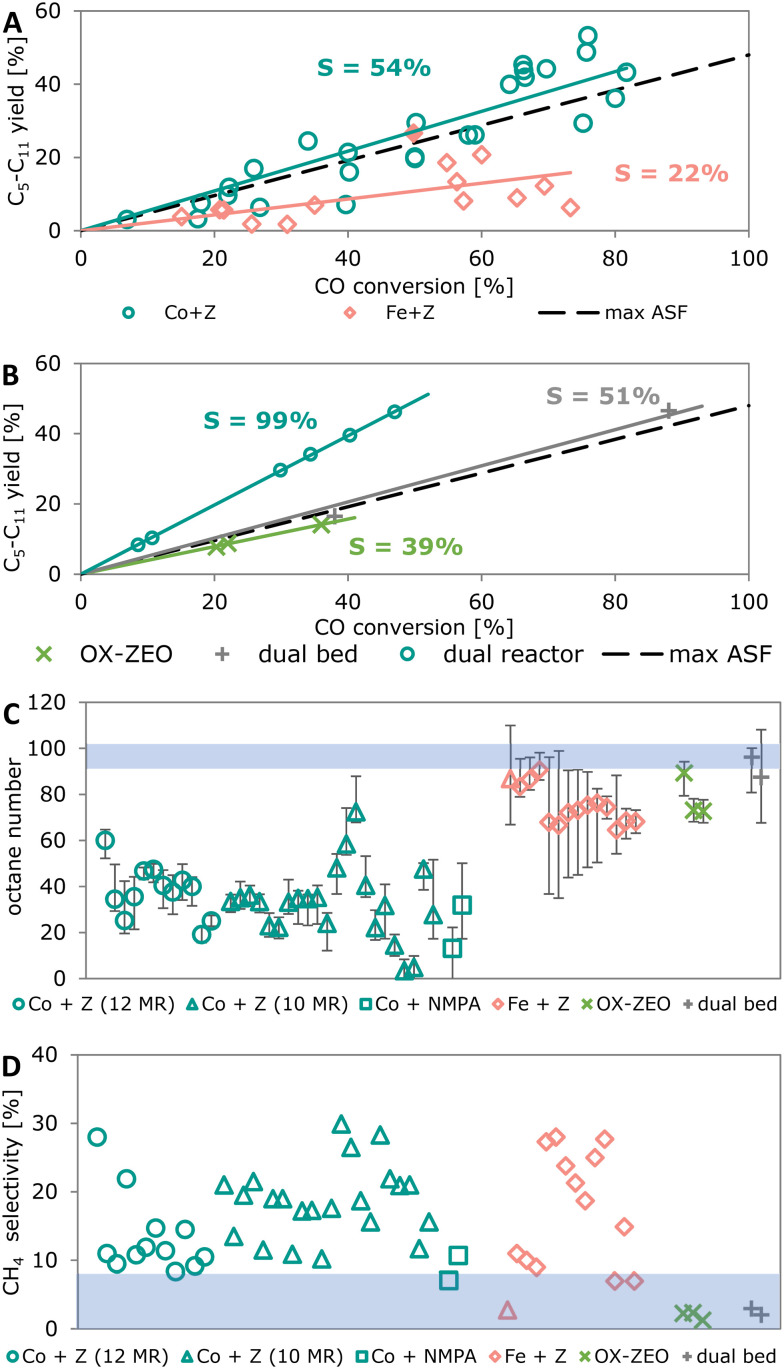
A: Yield of C_5_–C_11_ hydrocarbons as function of CO conversion for Co + Z and Fe + Z; B: yield of C_5_–C_11_ hydrocarbons as function of CO conversion for OX–ZEO, dual bed configuration and separated dual reactor processes C: the calculated octane number and D: methane selectivity for the combination of cobalt-based FT catalysts with zeolites (Co + Z), whereas zeolites were 12-membered ring or 10-membered ring zeolites or non-micro-porous solid acids (NMPA), iron-based FT catalysts with zeolite (Fe + Z), the OX–ZEO process and the combination of DME or FTO catalysts with zeolites in a dual bed configuration. The solid diamond in A indicates a combination of iron-based FT catalyst with zeolite showing reduced CO_2_ selectivity of 14.3%.^435^ A cobalt-carbide based FTO catalyst combined with a zeolite is shown in C and D as red triangle (first point of the Fe + Z series).^410^ The horizontal grey bars in C and D highlight the octane numbers needed to use the C_5_–C_11_ fraction directly as gasoline fuel and the typical methane selectivities of the HT-FTS for comparison, respectively.

Fig. 19-A and B show the yield to C_5_–C_11_ products for the bifunctional catalysts consisting of Co-based Fischer–Tropsch catalysts and solid acids (Co + Z), iron-based Fischer–Tropsch catalysts with zeolite (Fe + Z), the OX–ZEO process for the synthesis of gasoline (OX–ZEO) as function of CO conversion. Additionally, the C_5_–C_11_ yield of dual bed approaches is shown. Furthermore, Fig. 19-B contains the yield of C_5_–C_11_ hydrocarbons as function of CO conversion of two separate processes (dual reactor process, Fig. 17-B) that involves methanol synthesis and separation in the first process and the methanol to gasoline (MTG) as the second process.^357,438^ The slopes for the individual approaches correspond to the overall C_5_–C_11_ selectivity and take the formation of CO_2_ into account. As a reference the maximum yield as function of CO conversion resulting from the ASF distribution (48% selectivity for C_5_–C_11_) is shown as well.

Co + Z, Fe + Z and OX–ZEO show overall selectivities to C_5_–C_11_ of 54%, 22% and 39%, respectively. The dual bed process allows to produce C_5_–C_11_ products with 51% selectivity. The scattering is due to the fact that the C_5_–C_11_ selectivity is influenced by many parameters, such as reaction conditions, type of bifunctional catalyst, and nature of the zeolite. Fe + Z shows a low overall selectivity to C_5_–C_11_ products (22%) resulting from a moderate fraction of C_5_–C_11_ (10–55%) in the hydrocarbon products and much CO_2_ production due to the high WGS activity.^374,397^ Inhibiting the WGS can increase the C_5_–C_11_ selectivity (62%).^435^ Cobalt-based FT catalysts show low WGS activity, (only 1–4% CO_2_) thus Co + Z can have a higher overall selectivity to C_5_–C_11_ products (56%).^420^ The OX–ZEO process displays a large fraction of C_5_–C_11_ in the hydrocarbons (67–77%), but with a high WGS activity (∼50% CO_2_) the overall selectivity is reduced to 39%.^305,381^

The dual bed processes show a C_5_–C_11_ selectivity of 51%, which is comparable to the highest possible selectivity predicted by the ASF distribution. This selectivity results from a high fraction of C_5_–C_11_ in the hydrocarbon products (70–78%) and a significant WGS activity (32–38% CO_2_ formed) which reduces the overall C_5_–C_11_ selectivity.^357,438^ A combination of separate processes (MeOH synthesis, MeOH recovery, MTG process) could potentially show much higher overall selectivity of 96–99%, due to high selectivities of the MeOH synthesis (97–99%)^351,352^ and the MTG process (up to 99%)^441^ (data can be found in the ESI†).

Based on reported catalytic performance of bifunctional catalysts for the direct conversion of synthesis gas to gasoline we calculated the octane number of the corresponding C_5_–C_11_ fraction (Fig. 19-C) for different approaches: Co + Z, Fe + Z and OX–ZEO. Additionally, we added dual bed processes with direct DME synthesis or FTO catalyst in the first bed and a zeolite in the bed downstream. These processes have different temperatures for the individual catalyst beds but do not separate the intermediate products after the first catalytic conversion from unreacted reactants or formed side products. The detailed analysis and calculation of the octane numbers can be found in the ESI.†

The linear C_5_–C_11_ paraffin products of a Co-based FTS catalyst with ASF product distribution at *α* = 0.76 without zeolite have a low octane number (∼1.5). The combination of Co-based Fischer–Tropsch catalysts and a zeolite increases octane numbers to between 13 and 72. The use of zeolites with different pore dimensions, namely 10- or 12-membered-ring pores, does not affect the resulting octane number significantly. Iron-based Fischer–Tropsch catalyst mixed with zeolites show higher octane numbers of the C_5_–C_11_ products of 65–91. Also, the OX–ZEO process allows high octane products with an octane number of 73–89. The dual bed processes with different temperatures for the catalyst beds exhibit high octane numbers between 88 and 96. The horizontal grey bar in Fig. 19-C highlights the octane numbers needed to use the C_5_–C_11_ fraction directly as gasoline fuel. However, the octane number is often boosted by fuel additives, such as MTBE or ethanol.

The relatively low octane number of Co + Z can be explained by the mainly paraffinic products of Co-FTS, which hardly undergo isomerization, oligomerization or aromatization under typical reaction conditions.^467–469^ Fe + Z on the other hand is usually applied at higher temperatures, hence iron-based Fischer–Tropsch catalysts produce a higher fraction of olefins, allowing to form aromatics, boosting the octane number of the C_5_–C_11_ fraction drastically. OX–ZEO is not thermodynamically limited in the iso-paraffin fraction in the product and can additionally form aromatics and olefins with moderate selectivity. The analyzed OX–ZEO catalysts showed selectivity to iso-paraffin of 52–78%, olefin of 17–28% and aromatics of up to 16% in the C_5_–C_11_ hydrocarbon fraction.^305,381^ The dual bed process allows to operate the first bed (CO activation) at lower temperatures (260–270 °C), reducing CH_4_ selectivity and boosting CO conversion to 88% for the DME or FTO catalysts. Operating the second catalyst bed accommodating the zeolite (for gasoline synthesis) at higher temperatures of 320 °C enables the formation of aromatics, oligomerization of short olefins and eventually produce high-octane gasoline with high yields.^438^

Next to the octane number, the methane content is also important. In the past decades, Fischer–Tropsch catalysts and their process conditions have been optimized to reduce the CH_4_ selectivity. However, the addition of a zeolite leads to more methane.^374,419,421,432,470,471^ In Fig. 19-D the reported methane selectivities for Co + Z, Fe + Z, OX–ZEO and the dual bed processes are shown. The horizontal grey bar shows the typical methane selectivities of the HT-FTS for comparison. Co + Z and Fe + Z catalysts show high methane selectivities (7–30%), 2- to 3-fold higher than without the zeolite.^432,436^ OX–ZEO and the dual reactor processes only produce 2–3% methane similar to the values obtained for high-alpha FT catalysts.

The higher operating temperature and the resulting shift to lower alpha-values in the ASF distribution contributes to the high methane selectivity of FT + Z. For Co-based catalysts, the (acidity of the) support plays an important role.^472^ Methane production increases 3–4-fold than that predicted by the ASF model for catalysts supported on oxides with higher acidic character.^473^ XPS studies revealed that cobalt supported on a zeolite showed higher binding energy for Co 2p_3/2_ electrons compared to cobalt on a zeolite that has been covered with a layer of carbon prior to Co impregnation.^431^ Hence, the increased methane selectivity for cobalt catalysts supported directly on a zeolite might be explained by electronic effects.^474^ A reduced electron density of the cobalt particles causes weaker binding of hydrogen and stronger bonds between carbon and hydrogen of adsorbed CH_*x*_ species^475^ making the hydrogenation of CH_*x*_ species to CH_4_ energetically favored. Alternatively, the increased methane formation can be explained by a decreased reducibility of cobalt particles supported on zeolites^476,477^ or by small cobalt particles inside the zeolite pores, which also give rise to high methane selectivity.^478,479^

Co + Z shows the highest selectivity to the C_5_–C_11_ fraction (55%). However, the resulting octane number of this fraction is very low, hence it cannot be used as gasoline fuel directly. It has to be blended with a high fraction of additives. In terms of octane number for bifunctional processes, OX–ZEO and Fe + Z are the most promising, as they allow C_5_–C_11_ products with high octane numbers of up to ∼91 to form. Regarding the methane selectivity, only OX–ZEO and the dual bed processes can compete with the low methane selectivities achieved in FTS which are necessary for industrial application. In summary, the OX–ZEO process can form C_5_–C_11_ fuels with high octane number and little methane. If the WGS activity can be further reduced, this bifunctional catalyst has potential for the direct conversion of synthesis gas to gasoline fuel.

## 6. Summary and perspective

The transition to a more sustainable society is forcing a change in the current production processes of chemicals and fuels. A key step is the use of alternative feedstocks to the traditional fossil-based ones. Synthesis gas plays a crucial role due to the versatility of its sources and the various products it can create. In this regard, carbon sources from feedstocks such as CO_2_, organic waste, or biomass, together with the implementation of hydrogen production from renewable energy sources, can become central in this transition.^480^ Additionally, synthesis gas operation (production and conversion) is also feasible on a smaller scale, allowing a targeted production in remote locations.^76^ The use of bifunctional catalysts can expand the variety of products directly obtained from these sources.

In the past years, great progress has been realized in the field of synthesis gas conversion using bifunctional catalysts. A major advantage is that when combining different catalytic functions in a single reactor, synthesis gas conversion levels can lay far beyond the thermodynamic limitations of a first conversion step in a two-reactor system (Fig. 10 and 14).

In many cases in the first conversion step (*e.g.*, in methanol synthesis) no water is formed. Combining with a second conversion step can enhance the water content in the proximity of the CO activation catalyst, which can lead to the formation of CO_2_*via* the WGS reaction. This has been observed for the OX–ZEO process and DME synthesis, leading to CO_2_ selectivities up to 50% and 33%, respectively. The degree to which this happens is an important parameter. On the one hand CO_2_ formation lowers the carbon atom economy. On the other hand, the removal of water by the WGS reaction can also increase the catalyst lifetime and facilitate *in situ* production of additional hydrogen, favoring the utilization of carbon-rich synthesis gas over bifunctional catalysts. For the use of hydrogen-rich synthesis gas, the water-gas-shift activity of the OX–ZEO catalysts should be reduced, for example by recycling CO_2_. Our detailed analysis of recently published data for the direct synthesis of DME using bifunctional catalysts revealed an average DME selectivity of 62%. In contrast, a process consisting of two consecutive reactors (methanol synthesis and methanol dehydration) can achieve an overall DME selectivity of 88%. The lower selectivity for the bifunctional catalysts relates to the formation of CO_2_. *In situ* water removal by adsorption has proven an effective strategy to circumvent this limitation. The DME selectivity of bifunctional catalysts can be enhanced to 98% by *in situ* water removal by adsorption. Although the application in an industrial production scale still needs to be demonstrated, the Netherlands Organization for Applied Scientific Research (TNO) has taken the first steps in 2022 by building a containerized pilot reactor for sorption enhanced DME synthesis (SEDMES).^245,251,481–483^

In terms of C_2_–C_4_ olefins selectivity, neither OX–ZEO nor FTO can compete with a dual reactor process with methanol or DME synthesis in the first reactor and (D)MTO in a consecutive reactor (93% to C_2_–C_4_ olefins). The OX–ZEO process allows to form a high olefin fraction in the hydrocarbon products, but the high WGS activity and hence CO_2_ production reduces the overall selectivity to short olefins to 43% on average. FTO catalysts enable the production of short olefins with an average selectivity of 22% due to the high WGS activity and a limited high fraction of short olefins in the hydrocarbon products. Specific reduction of the WGS activity of FTO catalysts can increase the overall selectivity to 44%.

The situation is different for direct synthesis of aromatics from synthesis gas. OX–ZEO and the combination of iron-based Fischer–Tropsch catalysts with a zeolite (FT + Z) show selectivities to aromatics of 41% and 26%, respectively. Especially for the OX–ZEO process, this is only slightly lower than for the dual reactor process that comprises the synthesis of methanol or DME accompanied by aromatization based on hydrogen transfer (41%). This can make OX–ZEO competitive to this type of dual reactor process based on hydrogen transfer. A dual reactor process with aromatization based on dehydrogenation could theoretically achieve selectivities as high as 95%.

Three factors were considered for the analysis of the direct production of gasoline fuels using bifunctional catalysts: the overall selectivity to hydrocarbons in the gasoline range (C_5_–C_11_), the methane selectivity and the octane number of the resulting C_5_–C_11_ fraction. The combination of cobalt-based Fischer–Tropsch catalysts and zeolites (Co + Z) showed C_5_–C_11_ selectivities of 54%, which is beyond the maximum predicted by the ASF distribution. However, the methane selectivity was between 7% and 30% and octane numbers were rather low (30–50). Iron-based Fischer–Tropsch catalysts combined with zeolites (Fe + Z) yielded higher octane numbers (65–91). However, the methane selectivity was high (7–28%) and the overall selectivity to C_5_–C_11_ hydrocarbons was reduced to 22%, due to the high WGS activity. OX–ZEO produced a high-octane number of 73–89, low methane selectivity (only 1–2%) and medium overall selectivity to C_5_–C_11_ hydrocarbons (39%). This is slightly less than for a dual bed process with a zeolite downstream of a DME or FTO catalyst and dedicated reaction conditions for every catalyst bed: octane numbers between 88 and 96, methane selectivity 2–3% and medium C_5_–C_11_ selectivity (51%). Further efforts are then necessary to make gasoline production with bifunctional catalysis more attractive, particularly by reducing the water gas shift activity (and hence CO_2_ production).

Interest for bifunctional catalysts at industrial scale has been leaning towards DME and C_2_–C_4_ olefins production. The synthesis of DME from synthesis gas is mainly performed *via* the indirect route with a total annual production capacity of 10^7^ t per year.^23^ In 2003 the JFE Group in Japan finished the construction of a pilot plant designed for the direct DME synthesis with a capacity of 3.6 × 10^4^ t per year in Shiranuka-cho, Hokkaido, Japan using a slurry phase reactor.^484,485^ The Korea Gas Corporation launched a demonstration plant for the direct synthesis of DME with a capacity of 10 t per day already in 2004 at the Incheon KOGAS LNG terminal based on the KOGAS DME process.^23,222,486^ After successful operation, the design of a commercial production plant with 3 × 10^5^ t per year is in progress.^486^

From 2002 to 2007, a 100 ton per day demonstration plant project was successfully conducted by DME Development Corp. funded by 10 companies. Process performance analysis, catalyst life and long-term stable operation were assessed. Building on the technical data, the feasibility studies of commercial scale DME production from natural gas or coal were explored. Total Energies, JAPEX, INPEX and Toyota Tsusho, former members of the DME Development Corp., developed the technology in 2010. In 2016, those four companies transferred the technology patents to RenFud Corporation, which licenses the DME synthesis process technology and supplies proprietary catalysts. The first demonstrating operations with coke oven gas feed showed promising results, with 96% synthesis gas conversion, 93% selectivity to DME and 99.6% purity of DME.

**Fig. 20:**
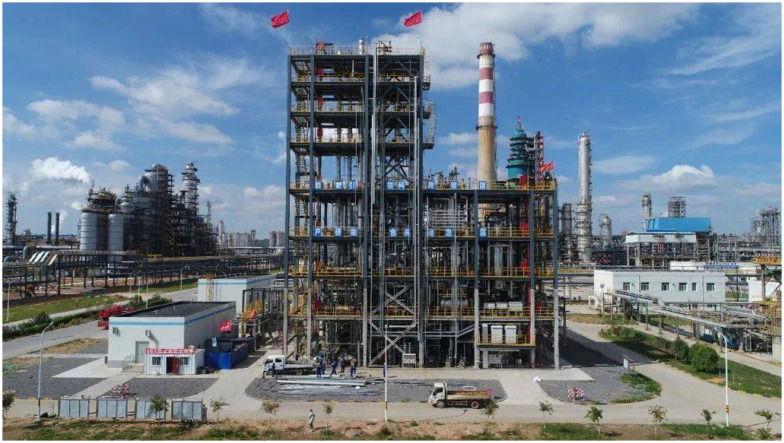
Photo of the industrial pilot plant to produce short olefins *via* the OX–ZEO to olefins (OXZEO^©^-TO) process, located in Shaanxi, China.^489^

In September 2019, the Dalian Institute of Chemical Physics and the Bureau of Major R&D Programs (Chinese Academy of Science) announced a cooperation between the Dalian Institute of Chemical Physics, the Chinese Academy of Sciences and Shaanxi Yanchang Petroleum (Group) Co., Ltd to perform industrial pilot trials with the aim to produce short olefins *via* the OX–ZEO process (Fig. 20). The capacity of this demonstration plant was 1000 t of short olefins per year and it is located in Shaanxi, China.^487,488^ In a first step synthesis gas is produced from coal, followed by the OX–ZEO process, converting 50% of the synthesis gas to C_2_–C_4_ olefins with 75% selectivity in a single pass.

At the moment research also focuses increasingly on the direct conversion of CO_2_ to chemicals and fuels.^490–495^ Bifunctional heterogeneous catalysts can be key to integrating CO_2_ activation and subsequent conversion to chemicals and fuels in an efficient way, contributing to carbon capture and utilization efforts. Despite the challenges for applications such as high costs, development of bifunctional catalysts for effective CO_2_ conversion is a promising topic with potentially beneficial environmental effects.^496^

## Data availability

The data supporting this article have been included as part of the ESI.†

## Author contributions

JLW and CHM contributed to this work by performing formal analysis, investigation, visualization and writing of the original draft. KPdJ and PEdJ contributed by supervision, validation, project administration and writing (review and editing).

## Conflicts of interest

There are no conflicts to declare.

## Supplementary Material

CY-014-D4CY00437J-s001

CY-014-D4CY00437J-s002
