# Supplementary material for: Recent advances in bifunctional synthesis gas conversion to chemicals and fuels with a comparison to monofunctional processes
Source: Catal Sci Technol. 2024 Jul 15;14(17):4799–842. doi: 10.1039/d4cy00437j (PMC11347923; doi:10.1039/d4cy00437j)
Supplement: CY-014-D4CY00437J-s001 [file CY-014-D4CY00437J-s001.pdf]

Electronic Supplementary Material (ESI) for Catalysis Science & Technology.
This journal is © The Royal Society of Chemistry 2024

Recent advances in bifunctional synthesis gas conversion to chemicals and fuels with a comparison to monofunctional processes

J.L. Weber, C. Hernández Mejía, K.P. de Jong, P.E. de Jongh

Content

List of Tables 2

List of Figures 2

1. DME 3

2. Olefins 8

3. Aromatics 16

4. Gasoline 24

4.1. Octane number 24

4.2. Analysis of published literature 27

5. References 38

List of Tables

Table S1: reported catalytic performance of bifunctional catalysts for the direct conversion of synthesis gas to DME 4
Table S2: reported catalytic performance of bifunctional catalysts for the direct conversion of synthesis gas to DME using *in-situ* water removal 6
Table S3: combined reported catalytic performance of catalysts for the conversion of synthesis gas to DME via a dual reactor process by combining methanol synthesis and methanol dehydration. 7
Table S4: reported catalytic performance of bifunctional catalysts for the direct conversion of synthesis gas to C_2-C_4 olefins via the OX-ZEO process 9
Table S5: reported catalytic performance of FTO catalysts for the direct conversion of synthesis gas to C_2-C_4 olefins 13
Table S6: combined reported catalytic performance of catalysts for the conversion of synthesis gas to C_2-C_4 olefins via a dual reactor process 15
Table S7: reported catalytic performance of bifunctional catalysts for the direct conversion of synthesis gas to aromatics via the OX-ZEO process 17
Table S8: reported catalytic performance of bifunctional catalysts for the direct conversion of synthesis gas to aromatics by combining FTO catalysts and zeolites 19
Table S9: combined reported catalytic performance of catalysts for the conversion of synthesis gas to aromatics via a dual reactor process 22
Table S10: average blending research octane numbers of C_5-C_{11} paraffins divided into number of branches. 24
Table S11: average blending research octane numbers of C_5-C_{11} olefins divided into number of branches. 25
Table S12: average blending research octane numbers of C_6-C_{11} aromatics divided into number of side chains. 26
Table S13: thermodynamic distribution of C_5-C_{11} n- and iso-paraffins between 200°C and 300°C. Calculated with Outotec HSC 4 at 20 bar pressure. 28
Table S14: reported catalytic performance of bifunctional catalysts for the direct conversion of synthesis gas to gasoline by combining Co-based FT catalysts and 12-membered ring zeolites 29
Table S15: reported catalytic performance of bifunctional catalysts for the direct conversion of synthesis gas to gasoline by combining Co-based FT catalysts and 10-membered ring zeolites 31
Table S16: reported catalytic performance of bifunctional catalysts for the direct conversion of synthesis gas to gasoline by combining Co-based FT catalysts and non-micro porous solid acids 33
Table S17: reported catalytic performance of bifunctional catalysts for the direct conversion of synthesis gas to gasoline by combining Fe-based FT catalysts and zeolites 34
Table S18: reported catalytic performance of bifunctional OX-ZEO catalysts for the direct conversion of synthesis gas to gasoline. 35
Table S19: reported catalytic performance of bifunctional catalysts for the direct conversion of synthesis gas to gasoline operated in dual bed mode with dedicated temperatures. 36
Table S20: combined reported catalytic performance of catalysts for the conversion of synthesis gas to gasoline combining methanol synthesis and MTG in individual processes. 37

List of Figures

Figure S1: average blending research octane number of C_5-C_{11} paraffins as function of number of branching. .. 24
Figure S2: average blending research octane number of C_5-C_{11} olefins as function of number of branching. 25
Figure S3: blending research octane number of linear C_5-C_{10} olefins as function of double bond position. 25
Figure S4: average blending research octane number of C_6-C_{11} aromatics as function of number of side chains. 26
Figure S5: average blending research octane number of aromatics as function of carbon number. 26

1. DME

To determine the overall carbon atom based selectivity of different processes to convert synthesis gas to DME, we calculated the yield of DME (Equation 1, Equation 2, Equation 3) from published data and plotted this against the corresponding CO conversion (Equation 4). The resulting slope gives the selectivity to DME and can be averaged over a set of data (Equation 5).

$$Y(DME) = \frac{\dot{n}_{out}(DME)}{\dot{n}_{in}(CO_x)} * f(DME) \qquad \text{Equation 1}$$

$$a\, CO_x + b\, H_2 \rightarrow c\, P1 + d\, P2 \qquad \text{Equation 2}$$

$$f(P1) = \frac{a}{c} \qquad \text{Equation 3}$$

$$X(CO_x) = \frac{\dot{n}_{in}(CO_x) - \dot{n}_{out}(CO_x)}{\dot{n}_{in}(CO_x)} \qquad \text{Equation 4}$$

$$S(DME) = \frac{Y(DME)}{X(CO_x)} \qquad \text{Equation 5}$$

Where,

Y: $yield$ [-]

$\dot{n}_{out}$: $molar\ carbon\ flow\ at\ reactor\ outlet\ [mol_C/s]$

$\dot{n}_{in}$: $molar\ carbon\ flow\ at\ reactor\ inlet\ [mol_C/s]$

f: $ratio\ of\ stoichiometric\ coefficients\ from\ the\ reaction\ equation$

$P1, P2$: $reaction\ products$

S: $selectivity$ [-]

X: $conversion$ [-]

We distinguished between bifunctional catalysts, bifunctional catalysts with *in-situ* water removal and a dual reactor process. For the bifunctional catalysts a methanol synthesis function is combined with a methanol dehydration function. Bifunctional catalysts with *in-situ* water removal additionally comprise a molecular sieve material that allows to remove water being formed during the reaction by adsorption. This can push the equilibrium of the reactants further to the side of DME and boost activity. The dual reactor approach shows the combination of methanol synthesis with consecutive methanol dehydration in separate processes. We used reported catalytic data of methanol synthesis catalysts and combined these with reported data of methanol dehydration catalysts. The calculation of the DME yields can be found in Table S1 (bifunctional catalysts), Table S2 (bifunctional catalysts with *in-situ* water removal) and Table S3 (dual reactor process).

Table S1: reported catalytic performance of bifunctional catalysts for the direct conversion of synthesis gas to DME

catalyst	MeOH cat	Solid acid	Temperature °C	Pressure bar(g)	CO conversion %	CO2 selectivity $\%_C$	DME selectivity $\%_C$	MeOH selectivity $\%_C$	Hydrocarbon selectivity $\%_C$	DME yield $\%_C$	ref
Zn@m-Al2O3	CuZnAl	γ-Al2O3	250	30	0.8	0.0	65.3	21.1	13.6	0.5	1
Cr/ZnO-SAPO46-M	CrZn	SAPO46	350	50	4.7	2.9	16.0	69.6	11.5	0.8	2
15.9%Nb/Al + CCMS	CuZnAl	Nb2O5-Al2O3	265	50	6	27.2	66.0	6.1	0.7	4.0	3
Cr/ZnO-SAPO46-PhyC	CrZn	SAPO46	350	50	6.9	6.0	34.8	49.1	10.2	2.4	2
CZA-4	CuZn	γ-Al2O3	250	50	7.1	29.9	67.0	1.1	2.0	4.8	4
5.9%Nb/Al + CCMS	CuZnAl	Nb2O5-Al2O3	265	50	8	27.7	64.9	6.8	0.6	5.2	3
Pd/silica-SZ	Pd-SiO2	HZSM-5	250	50	9	1.7	69.0	4.8	26.5	6.2	5
CZA-Z-IP	CuZnAl	γ-Al2O3	250	50	10	29.0	61.0	9.0	1.0	6.1	6
Pd/Ga(1:2)/γ-Al2O3	Pd	γ-Al2O3	250	50	10.9	33.6	52.4	1.9	12.1	5.7	7
CZA-2	CuZn	γ-Al2O3	250	50	11.1	30.7	64.3	1.2	3.9	7.1	4
Cu@m-Al2O3	CuZnAl	γ-Al2O3	250	30	13.2	25.7	68.5	4.7	1.2	9.0	1
CZA-ZSM5	CuZnAl	HZSM-5	250	40	13.9	20.9	14.4	64.1	0.6	2.0	8
CZA-NaY	CuZnAl	NaY	250	40	14.6	15.2	12.5	71.7	0.6	1.8	8
Pd/Ga(1:2)/γ-Al2O3	Pd	γ-Al2O3	260	50	14.6	35.1	46.2	1.8	16.9	6.7	7
CuZn@m-Al2O3	CuZnAl	γ-Al2O3	250	30	15.5	24.5	70.3	4.3	0.9	10.9	1
CZA-Z-CF	CuZnAl	γ-Al2O3	250	50	17	46.0	38.0	9.0	7.0	6.5	6
CuZn/m-Al2O3	CuZnAl	γ-Al2O3	250	30	17.4	15.9	70.2	11.8	2.1	12.2	1
Pd/Ga(1:2)/γ-Al2O3	Pd	γ-Al2O3	270	50	19.6	37.6	38.2	1.7	22.2	7.5	7
CZA-5	CuZn	γ-Al2O3	250	50	19.9	30.2	68.3	0.9	0.7	13.6	4
CZA-MA	CuZnAl	γ-Al2O3	275	50	22	30.3	52.5	17	0.2	11.5	9
CZA-Y	CuZnAl	Y	250	40	22.7	57.2	29.7	12.5	0.6	6.7	8
CZA-Y	CuZnAl	Y	250	40	22.9	59.8	26.8	12.8	0.6	6.1	10
CZA-ZSM5	CuZnAl	HZSM-5	250	40	23.3	26.2	27.9	45.0	0.9	6.5	10
CZA-1	CuZn	γ-Al2O3	250	50	24.4	31.8	58.7	6.2	3.4	14.3	4
CZA@HZSM-5-SS	CuZnAl	HZSM-5	250	30	26.3	14.3	28.7	56.3	0.6	7.5	11
FCZZ25(N)-10Z	CuZnZr	HZSM-5	250	45	29.4	31.5	60.1	8.3	0.1	17.7	12
CZA/ZrFER(5)	CuZnAl	FER	250	40	29.8	31.6	34.0	34.0	0.4	10.1	13
CZA-FER	CuZnAl	FER	250	40	30.2	27.8	28.7	42.8	0.7	8.7	8
CZA/ZrFER(0)	CuZnAl	FER	250	40	30.4	27.9	28.7	42.8	0.6	8.7	13
CZA-Z-CS	CuZnAl	γ-Al2O3	250	50	35	32.0	66.0	2.0	1.0	23.1	6
CZA/ZrFER(1)	CuZnAl	FER	250	40	35.3	36.7	40.8	22.1	0.4	14.4	13
T-4611+H-MOR 90	CuZnAl	H-MOR 90	250	50	37	43.9	25.2	1.3	29.6	9.3	14
CZAZr/HFER	CuZnAl	FER	250	50	38	33.0	65.0	2.0	0.0	24.7	15
0-CLZ-A	CuZrLa	γ-Al2O3	260	40	38.7	42.4	54.6	2.8	0.2	21.1	16
13 wt-% Cu + HZSM-5 (140)	CuZn	HZSM-5	280	50	40	-	-	2.6	-	25.0	17
CZA/ZrFER(K)	CuZnAl	FER	250	40	40.8	33.4	37.5	27.7	1.4	15.3	13

Table S1: reported catalytic performance of bifunctional catalysts for the direct conversion of synthesis gas to DME (continued)

catalyst	MeOH cat	Solid acid	Temperature °C	Pressure bar(g)	CO conversion %	CO2 selectivity $\%_C$	DME selectivity $\%_C$	MeOH selectivity $\%_C$	Hydrocarbon selectivity $\%_C$	DME yield $\%_C$	ref
CZA/Al(10)-FER	CuZnAl	FER	250	35	43	22.2	74.1	2.8	1.0	31.9	18
g-Al2O3	CuZnAl	γ-Al2O3	260	40	44	25.0	70.5	3.8	0.8	31.0	19
CZA-Z-OX	CuZnAl	γ-Al2O3	250	50	45	32.0	66.0	1.0	1.0	29.7	6
CuZnAl/SAPO11-M	CuZnAl	SAPO11	250	50	46.2	6.1	43.8	48.3	1.9	20.2	20
CZA/Al(0)-FER	CuZnAl	FER	250	35	46.6	24.1	70.9	2.9	2.1	33.0	18
C/Z-PC	CuZnAl	HZSM-5	250	30	47.6	31.1	66.3	3.4	0.2	31.6	21
CZA-Z	CuZnAl	γ-Al2O3	250	50	48	30.0	69.0	1.0	0.0	33.1	6
6-CLZ-A	CuZrLa	γ-Al2O3	260	40	48.2	33.5	63.7	2.6	0.2	30.7	16
CZA-FER	CuZnAl	FER	250	40	49	33.7	58.2	7.8	0.3	28.5	10
CZA/ZrFER(3)	CuZnAl	FER	250	40	49	33.7	58.2	7.8	0.3	28.5	13
25STA@CZA-MA	CuZnAl	γ-Al2O3 + $H_4[SiW_{12}O_{40}]$	275	50	49	31.6	59.8	8	0.4	29.3	9
NbOPO4	CuZnAl	NbOPO4	260	40	53	25.0	72.0	2.3	0.8	38.2	19
C/Z-P	CuZnAl	HZSM-5	250	30	54.5	31.3	65.3	2.3	1.1	35.6	21
18-CLZ-A	CuZrLa	γ-Al2O3	260	40	54.6	30.7	67.3	2.0	0.0	36.7	16
12-CLZ-A	CuZrLa	γ-Al2O3	260	40	56.7	29.3	69.0	1.7	0.0	39.1	16
C/Z-G	CuZnAl	HZSM-5	250	30	57.4	31.4	64	2.1	2.5	36.7	21
FCZZ25(N)-10Z	CuZnZr	HZSM-5	275	45	57.7	32.0	62.7	5.0	0.3	36.2	12
CuZnAl/SAPO11-PhyC	CuZnAl	SAPO11	250	50	58.5	9.1	82.1	8.4	0.5	48.0	20
CZA/ZrFER(NH3)	CuZnAl	FER	250	40	59.4	34.7	62.9	1.9	0.5	37.4	13
T-4611+γ-Al2O3	CuZnAl	γ-Al2O3	250	50	61	31.8	67.0	1.1	0.2	40.9	14
CZA/Al(2.5)-FER	CuZnAl	FER	250	35	61.8	25.6	71.4	2.5	0.5	44.1	18
Nb2O5·nH2O	CuZnAl	Nb2O5·nH2O	260	40	62	25.0	66.8	6.0	2.3	41.4	19
CZA/Al(5)-FER	CuZnAl	FER	250	35	62.1	26.8	69.4	3.0	0.7	43.1	18
CZA(A)	CuZnAl	γ-Al2O3	250	50	65.8	17.0	55.6	26.9	0.5	36.6	22
T-4611+H-MFI 90	CuZnAl	H-MFI 90	250	50	66	49.0	32.7	3.1	15.2	21.6	14
T-4611+H-MFI 400	CuZnAl	H-MFI 400	250	50	68	31.5	66.8	1.5	0.2	45.4	14
FCZZ25(N)-10Z	CuZnZr	HZSM-5	300	45	68	30.6	63.8	5.1	0.5	43.4	12
g-Al2O3	CuZnAl	γ-Al2O3	280	40	69	27.0	70.5	3.0	1.5	48.6	19
NbOPO4	CuZnAl	NbOPO4	280	40	73	27.0	70.5	2.3	1.5	51.5	19
Nb2O5·nH2O	CuZnAl	Nb2O5·nH2O	280	40	75	27.0	69.0	2.3	3.8	51.8	19
CZA@HZSM-5-EtOH	CuZnAl	HZSM-5	250	30	76.5	26.7	70.8	2.5	0.1	54.2	11
CuO–ZnO–Al2O3/MgZ1	CuZnAl	HZSM-5	260	40	96.3	30.5	64.5	4.6	0.4	62.1	23

Table S2: reported catalytic performance of bifunctional catalysts for the direct conversion of synthesis gas to DME using *in-situ* water removal

catalyst	MeOH cat	Solid acid	Temperature °C	Pressure bar(g)	CO conversion %	CO2 selectivity $\%_C$	DME selectivity $\%_C$	MeOH selectivity $\%_C$	Hydrocarbon selectivity $\%_C$	DME yield $\%_C$	ref
CZA_comm	CuZnAl	γ-Al2O3	275	25	55	4.0	95.0	-	-	52.3	24
mech mixture 1/1, 3mm	modelling				68.1[1]		97.8			66.6	25
Cu/ZnO/Al2O3 + zeolite (3Å)	CuZnAl	γ-Al2O3	275	25	70	-	92.9	-	-	65.0	26
MeOH@DME 1/1, 3mm	modelling				72.6[1]		97.5			70.8	25
MeOH@DME 2/1, 3mm	modelling				76.8[1]		97.9			75.2	25
DME@MeOH 1/1, 3mm	modelling				77.5[1]		97.2			75.3	25
hydrid 1/1, 3mm	modelling				78.4[1]		97.4			76.4	25
mech mixture 1/1, 1.5mm	modelling				78.6[1]		97.7			76.8	25
mech mixture 1/1, 1mm	modelling				81.4[1]		97.9			79.7	25
Cu/ZnO/Al2O3 + zeolite (3Å)	CuZnAl	γ-Al2O3	250	24	90.1[1]		99.2			89.4	27,28
Cu/ZnO/Al2O3 + LTA (3Å)	CuZnAl	γ-Al2O3	252	25	94.5[1]		99.0			93.6	29

[1] CO_x conversion, experiments were conducted with mixture of CO, CO_2 and H_2

Table S3: combined reported catalytic performance of catalysts for the conversion of synthesis gas to DME via a dual reactor process by combining methanol synthesis and methanol dehydration.

	CO conversion %	CO_2 selectivity $\%_C$	methanol selectivity $\%_C$	DME selectivity from methanol $\%_C$	DME selectivity from synthesis gas $\%_C$	yield $\%_C$	ref
**dual reactor process**							
**MeOH**							
Cu/ZnO/Al2O3	8.6		97.7			8.4	30
2Cu_MCF 10.7	10.7		97			10.4	31
Cu/ZnO/Al2O3	29.9		99.6			29.8	30
Cu/ZnO/Al2O3	34.4		99.8			34.3	30
Cu/ZnO/Al2O3	40.3		98.7			39.8	30
Cu/ZnO/Al2O3	47.0		98.9			46.5	30
**MeOH + DME**							
Al-HMS-10	8.6	0		100 (at 89% methanol conversion)	87.0	7.5	32
Al-HMS-10	10.7	0		100 (at 89% methanol conversion)	86.3	9.2	32
Al-HMS-10	29.9	0		100 (at 89% methanol conversion)	88.6	26.5	32
Al-HMS-10	34.4	0		100 (at 89% methanol conversion)	88.8	30.6	32
Al-HMS-10	40.3	0		100 (at 89% methanol conversion)	87.8	35.4	32
Al-HMS-10	47.0	0		100 (at 89% methanol conversion)	88.0	41.4	32

2. Olefins

The overall selectivity of the conversion of synthesis gas to C_2-C_4 olefins was analyzed by calculation of the yield to C_2-C_4 olefins (Equation 6) and dividing by the conversion to obtain the selectivity (Equation 7). The olefins analyzed own different carbon atom numbers, hence the yield was directly calculated using the amount of carbon atoms within the C_2-C_4 olefins formed ($n_{out}(C_{olefins})$ in Equation 6).

Three different approaches were analyzed to convert synthesis gas into olefins, namely OX-ZEO, Fischer-Tropsch to olefins (FTO) and a dual reactor process. The OX-XEO and FTO process both include recent studies with decreased water-gas-shift activity and are labeled with *low* CO_2. The dual reactor approach shows the combination of methanol synthesis with consecutive methanol-to-olefins (MTO) reaction in separate processes. We used reported catalytic data of methanol synthesis catalysts and combined these with reported data of MTO catalysts. The calculation of the C_2-C_4 olefin yields can be found in Table S4 (OX-ZEO), Table S5 (FTO) and Table S6 (dual reactor process).

$$Y(olefins) = \frac{\dot{n}_{out}(C_{olefins})}{\dot{n}_{in}(CO_x)} \quad \text{Equation 6}$$

$$S(olefins) = \frac{Y(olefins)}{X(CO_x)} \quad \text{Equation 7}$$

Where,

Y: $yield$

$\dot{n}_{out}$: $molar\ flow\ at\ reactor\ outlet$

$\dot{n}_{in}$: $molar\ flow\ at\ reactor\ inlet$

$C_{olefins}$: $carbon\ atoms\ in\ olefin\ molecules$

S: $selectivity$

X: $conversion$

Table S4: reported catalytic performance of bifunctional catalysts for the direct conversion of synthesis gas to C_2-C_4 olefins via the OX-ZEO process

catalyst	CO conversion %	CO2 selectivity $\%_C$	C_2-C_4 olefins in hydrocarbons $\%_C$	hydrocarbons selectivity $\%_C$	C_2-C_4 olefin selectivity $\%_C$	C_2-C_4 olefin yield $\%_C$	ref
**OX-ZEO**							
ZrO2	4	42.0	79.0	58.0	45.8	1.8	33
ZnCr/SAPO-17, 1 Mpa	4.3	47.9	75.6	52.1	39.4	1.7	34
Mn/Ga2O3	5.3	44.8	61.5	55.2	33.9	1.8	35
ZnO	6	42.0	26.5	58.0	15.4	0.9	33
ZnCrOx/MSAPO	6	45.0	68.0	55.0	37.4	2.2	36
MnxZry/SAPO34 Mn:Zr = 1 : 0	6.9	24.3	68.5	75.7	51.9	3.6	37
ZnAlOx/CHA Si/Al=307	8	40.0	86.0	60.0	51.6	4.1	38
MnxZry/SAPO34 Mn:Zr = 1 : 0,25	8.5	48.4	49.3	51.6	25.4	2.2	37
MG-(SM)	8.6	44.5	68.3	55.5	37.9	3.3	35
MnxZry/SAPO34 Mn:Zr = 1 : 0,5	8.8	46.0	50.2	54.0	27.1	2.4	37
ZnAlOx/CHA Si/Al=237	9	40.0	85.0	60.0	51.0	4.6	38
MnxZry/SAPO34 Mn:Zr = 1 : 4	9.3	47.2	52.2	52.8	27.6	2.6	37
ZnAlOx/CHA Si/Al=138	9.5	40.0	80.0	60.0	48.0	4.6	38
MnxZry/SAPO34 Mn:Zr = 1 : 1	9.7	43.9	43.5	56.1	24.4	2.4	37
ZnAlOx/CHA Si/Al=76	10	45.0	75.0	55.0	41.3	4.1	38
GaCeOx	10	42	79	58.0	45.8	4.6	39
MnxZry/SAPO34 Mn:Zr = 1 : 2	10.6	45.3	59.6	54.7	32.6	3.5	37
ZnCrOx/MSAPO	12	45.0	72.0	55.0	39.6	4.8	36
ZnAlOx/CHA Si/Al=20	12	47.0	56.0	53.0	29.7	3.6	38
ZnAlOx/CHA Si/Al=38	12	46.0	67.0	54.0	36.2	4.3	38
ZnCr/SAPO-17, 2 Mpa	12.6	47.9	87.3	52.1	45.5	5.7	34
ZnCrOx + H−SSZ-13 (27C)	12.6	51.3	60.9	48.7	29.7	3.7	40
InZr/SAPO34	13.1	40.0	79.9	60.0	47.9	6.3	41
ZnCrOx + SAPO-35(0.17)	13.9	46.9	74.2	53.1	39.4	5.5	42
ZnAl2O4/SAPO-35	15	44.0	56.0	56.0	31.4	4.7	43
SP17(48h)	15.6	47.8	88.7	52.2	46.3	7.2	44
ZnCrOx + H−SSZ-13 (23C)	16	50.2	66.1	49.8	32.9	5.3	40
InZr/SAPO34	16.2	40.0	73.7	60.0	44.2	7.2	41
ZnCr/SAPO-17, 370°C	16.4	42.5	91.4	57.5	52.6	8.6	34
ZnCrOx + SAPO-35(0.11)	16.5	47.4	75.1	52.6	39.5	6.5	42
Zn-ZrO2 (1:64)/H-SSZ-13-45H	17	42.0	76.7	58.0	44.5	7.6	33
ZnCrOx/MSAPO	17	45.0	73.0	55.0	40.2	6.8	36
ZnO-ZrO2/SAPO-34 0,12mmol/g	17	43.0	76.0	57.0	43.3	7.4	45
ZnCrOx + SAPO-18(0.030)	17.2	49.9	75.1	50.1	37.6	6.5	42
SP17(72h)	17.2	46.7	86.2	53.3	45.9	7.9	44

Table S4: reported catalytic performance of bifunctional catalysts for the direct conversion of synthesis gas to C_2-C_4 olefins via the OX-ZEO process (continued)

catalyst	CO conversion %	CO2 selectivity %c	C_2-C_4 olefins in hydrocarbons %c	hydrocarbons selectivity %c	C_2-C_4 olefin selectivity %c	C_2-C_4 olefin yield %c	ref
ZnCrOx + H−SSZ-13 (19C)	17.3	49.7	53.9	50.3	27.1	4.7	40
ZnCr/SAPO-17, 360°C	17.4	38.4	91.5	61.6	56.4	9.8	34
ZnCr/SAPO-17, 380°C	17.5	47.0	90.9	53.0	48.2	8.4	34
ZnCrOx + SAPO-18(0.054)	18.2	49.4	69.9	50.6	35.4	6.4	42
ZnCr/Low Si AlPO-18	19					8.4	46
SP17(120h)	19.3	48.5	81.8	51.5	42.1	8.1	44
SP17(96h)	19.4	46.4	87	53.6	46.6	9.0	44
ZnCrOx + H−SSZ-13 (19S)	19.7	48.6	68.1	51.4	35.0	6.9	40
ZnCrOx + SAPO-18(0.048)	19.9	49.2	68.6	50.8	34.8	6.9	42
ZnCrOx/MSAPO	20	45.0	80.0	55.0	44.0	8.8	36
ZnO-ZrO2/SAPO-34 0,16mmol/g	20	40.0	77.0	60.0	46.2	9.2	45
ZnCrOx + H−SSZ-13 (26S)	20	48.9	71.6	51.1	36.6	7.3	40
ZnCrOx + H−SSZ-13 (12S)	20.7	49.0	55.1	51.0	28.1	5.8	40
ZnCrOx + H−SSZ-13 (23S)	20.9	48.0	70.8	52.0	36.8	7.7	40
ZnAl2O4/SAPO-18	21	44.0	69.0	56.0	38.6	8.1	43
Zn-ZrO2 (1:32)/H-SSZ-13-45H	22	42.0	74.4	58.0	43.2	9.5	33
Zn-ZrO2 (4:1)/H-SSZ-13-45H	22	42.0	35.1	58.0	20.4	4.5	33
ZnCrOx/MSAPO	22	45.0	71.0	55.0	39.1	8.6	36
ZnCr/SAPO-17, 390°C	22	48.6	90.0	51.4	46.3	10.2	34
GaMnOx	22	42	89	58.0	51.6	11.4	39
ZnAl2O4/SAPO-17	23	42.0	65.0	58.0	37.7	8.7	43
Zn-ZrO2 (1:16)/H-SSZ-13-45H	24	42.0	74.0	58.0	42.9	10.3	33
ZnO-ZrO2/SAPO-34 0,22mmol/g	24	41.0	81.0	59.0	47.8	11.5	45
ZnAl2O4/SAPO-34	24	44.0	80.0	56.0	44.8	10.8	43
ZnCr/Low Si AlPO-18	25					11.3	46
ZnCr/Low Si AlPO-18	25					10.6	46
ZnCrOx-MOR#2-py	26	45.0	73.0	55.0	40.2	10.4	47
ZnCr/SAPO-17, 400°C	26.2	48.6	88.3	51.4	45.4	11.9	34
ZnCr/SAPO-17, 3 Mpa	26.2	48.6	88.3	51.4	45.4	11.9	34
Zn-ZrO2 (1:4)/H-SSZ-13-45H	27	42.0	65.5	58.0	38.0	10.3	33
ZnO-ZrO2/SAPO-34 0,26mmol/g	27	41.0	75.0	59.0	44.3	11.9	45
InZr/SAPO34	27.7	40.0	73.6	60.0	44.2	12.2	41
Zn-ZrO2 (2:1)/H-SSZ-13-45H	28	42.0	54.2	58.0	31.4	8.8	33
ZnCrOx/MSAPO	28	45.0	71.0	55.0	39.1	10.9	36
ZnCr/SAPO-17, 410°C	28.5	48.4	85.3	51.6	44.0	12.5	34
SP34	28.5	45.2	87.1	54.8	47.7	13.6	44

Table S4: reported catalytic performance of bifunctional catalysts for the direct conversion of synthesis gas to C_2-C_4 olefins via the OX-ZEO process (continued)

catalyst	CO conversion<br>%	CO2 selectivity<br>%$_C$	C_2-C_4 olefins in hydrocarbons<br>%$_C$	hydrocarbons selectivity<br>%$_C$	C_2-C_4 olefin selectivity<br>%$_C$	C_2-C_4 olefin yield<br>%$_C$	ref
SP18	28.7	45	87	55.0	47.9	13.7	44
Zn-ZrO2 (1:1)/H-SSZ-13-45H	29	42.0	61.8	58.0	35.9	10.4	33
ZnCrOx/MSAPO	30	45.0	73.0	55.0	40.2	12.0	36
ZnCr/SAPO-34	30					12.6	46
ZnO-ZrO2/SAPO-34 0,27mmol/g	30	41.0	70.0	59.0	41.3	12.4	45
InZr/SAPO34	30.7	40.0	67.3	60.0	40.4	12.4	41
ZnCr/Low Si AlPO-18	31					13.3	46
ZA-CP	33.9	43.5	75	56.5	42.4	14.4	48
ZnCr/Low Si AlPO-18	34					14.3	46
ZnCr/Low Si AlPO-18	34					15.3	46
ZnCrOx-SAPO-18 Si/Al = 0,011	35.5	41.4	82.0	58.6	48.1	17.1	49
ZnCr/SAPO-17, 4 Mpa	38.2	47.6	87.3	52.4	45.7	17.5	34
ZA-RP	39.2	43.3	73.3	56.7	41.6	16.3	48
ZnCr/SAPO-34	40					16.4	46
ZA-SP	40.2	44.6	74.1	55.4	41.1	16.5	48
ZnCr/Low Si AlPO-18	43					18.1	46
ZnCr/Low Si AlPO-18	43					18.9	46
GaZrOx	44.5	42	89	58.0	51.6	23.0	39
ZnCrOx-SAPO 450-900µm	47	41.0	72.0	59.0	42.5	20.0	50
ZnCrOx-SAPO-18 Si/Al = 0,054	47.1	41.8	61.0	58.2	35.5	16.7	49
ZnCr/Low Si AlPO-18	49					20.6	46
ZnCr/Low Si AlPO-18	49					21.1	46
ZnCrOx-SAPO-18 Si/Al = 0,045	49.5	40.9	69.0	59.1	40.8	20.2	49
ZnCrOx-SAPO 150-74µm	58	40.0	72.0	60.0	43.2	25.1	50
ZnCr/SAPO-34	59					22.1	46
ZnCrOx-SAPO 20-50µm	59	39.0	65.0	61.0	39.7	23.4	50
ZnCrOx-SAPO 200-300µm	60	39.0	76.0	61.0	46.4	27.8	50
ZnCrOx-GeAPO-$18_{0.027}$	85	32	83	68	56.5	48	51
**low CO2 OX-ZEO:**							
Zn0.3Ce2-yZryO4	5	4.0	60	96.0	57.6	2.9	52
Zn0.3Ce2-yZryO4	6.5	5.5	77	94.5	72.8	4.7	52
Zn0.3Ce2-yZryO4	6.5	8.5	78	91.5	71.4	4.6	52
Zn0.3Ce2-yZryO4	7	11.0	76	89.0	67.6	4.7	52
Zn0.3Ce2-yZryO4	7	12.0	73	88.0	64.2	4.5	52
Zn0.3Ce2-yZryO4	7	10.0	77	90.0	69.3	4.9	52
Zn0.3Ce2-yZryO4	7	11.0	78	89.0	69.4	4.9	52

Table S4: reported catalytic performance of bifunctional catalysts for the direct conversion of synthesis gas to C_2-C_4 olefins via the OX-ZEO process (continued)

catalyst	CO conversion<br>%	CO2 selectivity<br>%$_C$	C_2-C_4 olefins in hydrocarbons<br>%$_C$	hydrocarbons selectivity<br>%$_C$	C_2-C_4 olefin selectivity<br>%$_C$	C_2-C_4 olefin yield<br>%$_C$	ref
Zn0.3Ce2-yZryO4	7	12.0	75	88.0	66.0	4.6	52
Zn0.3Ce2-yZryO4	7.5	13.0	75	87.0	65.3	4.9	52
Zn0.3Ce2-yZryO4	7.5	12.0	75	88.0	66.0	5.0	52
Zn0.3Ce2-yZryO4	8	15.0	75	85.0	63.8	5.1	52
Zn0.3Ce2-yZryO4	8	12.5	72	87.5	63.0	5.0	52
Zn0.3Ce2-yZryO4	9	22.0	76	78.0	59.3	5.3	52
Zn0.3Ce2-yZryO4	10	23.0	72	77.0	55.4	5.5	52
Zn-Cr@SAPO capsule catalyst	10.4	36.0	63.8	64.0	40.8	4.2	53
Zn0.3Ce2-yZryO4	12	26.0	59	74.0	43.7	5.2	52

Table S5: reported catalytic performance of FTO catalysts for the direct conversion of synthesis gas to C_2-C_4 olefins

catalyst	CO conversion %	CO_2 selectivity $\%_C$	C_2-C_4 olefins in hydrocarbons $\%_C$	hydrocarbon selectivity $\%_C$	C_2-C_4 olefin selectivity $\%_C$	C_2-C_4 olefin yield $\%_C$	ref
**FTO**							
CoMn carbide nano prisms	6.3	48.3	45.1	51.7	23.3	1.5	54
Fe/SiO2	10.1	29.0	29.6	71.0	21.0	2.1	55
CoMn carbide nano prisms	11.5	48.0	50.0	52.0	26.0	3.0	54
CoMn carbide nano prisms	14.3	48.4	44.3	51.6	22.9	3.3	54
Co1Mn3–Na2S	18	3.0	30.0	97.0	29.1	5.2	56
Co1Mn3–Na2S2O3	22	3.0	25.0	97.0	24.3	5.3	56
CoMn carbide nano prisms	23.6	48.0	41.2	52.0	21.4	5.1	54
Co3Mn1–Na2S	25	13.0	20.0	87.0	17.4	4.4	56
N5 @340°C	27.4	47.8	43.0	52.2	22.4	6.2	57
CoMn carbide nano prisms	28.6	46.6	31.9	53.4	17.0	4.9	54
Co3Mn1	31	2.0	17.0	98.0	16.7	5.2	56
CoMn carbide nano prisms	31.8	47.3	60.8	52.7	32.0	10.2	54
6Fe	32.7	21.2	17.5	78.8	13.8	4.5	58
4Fe-Zn	34.1	33.1	13.3	66.9	8.9	3.0	58
N1 @340°C	38.3	48.0	52.1	52.0	27.1	10.4	57
5Fe-1.2Na	48.7	21.9	20.3	78.1	15.9	7.7	58
FeBi/CNT	50.7	46.0	36.1	54.0	19.5	9.9	55
2Fe.Zn0.2Na (SC-I)3	52.3	41.9	50.5	58.1	29.3	15.3	59
FePb/CNT	56.8	48.0	35.8	52.0	18.6	10.6	55
Fe/CNT	57.3	40.0	32.4	60.0	19.4	11.1	55
2Fe.Zn0.2Na (AH-I)	60.2	39.1	47.7	60.9	29.0	17.5	59
1Fe-Zn-3.4Na	63	22.5	19.9	77.5	15.4	9.7	58
5AFeP	69	45.0	51.0	55.0	28.1	19.4	60
2Fe-Zn-0.81Na	77.2	23.8	22.7	76.2	17.3	13.4	58
FeBi/CNT	78.3	47.0	35.2	53.0	18.7	14.6	55
2Fe.Zn0.2Na (SC-I)2	79.3	40.6	50.3	59.4	29.9	23.7	59
2Fe.Zn0.1Na (AH-I)	81.1	39.25	42.8	60.8	26.0	21.1	59
3Fe-Zn-0.36Na	82.7	25.9	22.9	74.1	16.9	14.0	58
10IMP	86	47.0	52.0	53.0	27.6	23.7	61
N5 @370°C	87.8	44.7	34.4	55.3	19.0	16.7	57
N1 @370°C	90	46.3	37.3	53.7	20.0	18.0	57
FePb/CNT	96	50.0	28.4	50.0	14.2	13.6	55
2Fe.Zn0.2Na (SC-I)1	97.4	34.4	50	65.6	32.8	31.9	59
**low CO2 FTO:**							
FeZn@16.9-SiO2-c	52.2	8.5	44.5	91.5	40.7	21.2	62

Table S5: reported catalytic performance of FTO catalysts for the direct conversion of synthesis gas to C_2-C_4 olefins (continued)

catalyst	CO conversion %	CO_2 selectivity $\%_C$	C_2-C_4 olefins in hydrocarbons $\%_C$	hydrocarbon selectivity $\%_C$	C_2-C_4 olefin selectivity $\%_C$	C_2-C_4 olefin yield $\%_C$	ref
Fe@SAPO-34	55.4	17.1	52.6	82.9	43.6	24.2	63
FeZn@7.3-SiO2-c	63.1	8.8	47.3	91.2	43.1	27.2	62
FeZn@4.1-SiO2-c	65.3	7.2	52.6	92.8	48.8	31.9	62
FeZn@2.4-SiO2-c	77.8	11.9	50.7	88.1	44.7	34.8	62
FeZn@1.3-SiO2-c	82.3	17.2	50.4	82.8	41.7	34.3	62

Table S6: combined reported catalytic performance of catalysts for the conversion of synthesis gas to C_2-C_4 olefins via a dual reactor process

catalyst	CO conversion	CO_2 selectivity	methanol selectivity	C_2-C_4 olefin selectivity from methanol	C_2-C_4 olefin selectivity from synthesis gas	yield	ref
	%	$\%_C$	$\%_C$	$\%_C$	$\%_C$	$\%_C$	
**dual reactor process**							
**MeOH**							
Cu/ZnO/Al2O3	8.6		97.7			8.4	30
2Cu_MCF 10.7	10.7		97.0			10.4	31
Cu/ZnO/Al2O3	29.9		99.6			29.8	30
Cu/ZnO/Al2O3	34.4		99.8			34.3	30
Cu/ZnO/Al2O3	40.3		98.7			39.8	30
Cu/ZnO/Al2O3	47.0		98.9			46.5	30
**MeOH + MTO**							
SSZ-13	8.6	0.0		94.1	91.9	7.9	64
meso-Z	8.6	0.0		95.5	93.3	8.0	64
meso-Z-22-4-4	8.6	0.0		93.5	91.3	7.9	64
meso-Z-22-4-4-sil	8.6	0.0		94.2	92.0	7.9	64
SSZ-13	10.7	0.0		94.1	91.3	9.8	64
meso-Z	10.7	0.0		95.5	92.6	9.9	64
meso-Z-22-4-4	10.7	0.0		93.5	90.7	9.7	64
meso-Z-22-4-4-sil	10.7	0.0		94.2	91.4	9.8	64
SSZ-13	29.9	0.0		94.1	93.7	28.0	64
meso-Z	29.9	0.0		95.5	95.1	28.4	64
meso-Z-22-4-4	29.9	0.0		93.5	93.1	27.8	64
meso-Z-22-4-4-sil	29.9	0.0		94.2	93.8	28.1	64
SSZ-13	34.4	0.0		94.1	93.9	32.3	64
meso-Z	34.4	0.0		95.5	95.3	32.8	64
meso-Z-22-4-4	34.4	0.0		93.5	93.3	32.1	64
meso-Z-22-4-4-sil	34.4	0.0		94.2	94.0	32.3	64
SSZ-13	40.3	0.0		94.1	92.9	37.4	64
meso-Z	40.3	0.0		95.5	94.3	38.0	64
meso-Z-22-4-4	40.3	0.0		93.5	92.3	37.2	64
meso-Z-22-4-4-sil	40.3	0.0		94.2	93.0	37.5	64
SSZ-13	47.0	0.0		94.1	93.1	43.7	64
meso-Z	47.0	0.0		95.5	94.4	44.4	64
meso-Z-22-4-4	47.0	0.0		93.5	92.5	43.5	64
meso-Z-22-4-4-sil	47.0	0.0		94.2	93.2	43.8	64

3. Aromatics

The overall selectivity of the conversion of synthesis gas to aromatics was analyzed analog to the selectivity of C_2-C_4 olefins (Equation 8 and Equation 9).

$$Y(aromatics) = \frac{n_{out}(C_{aromatics})}{n_{in}(CO_x)} \qquad \text{Equation 8}$$

$$S(aromatics) = \frac{Y(aromatics)}{X(CO_x)} \qquad \text{Equation 9}$$

Where,

Y:$yield$

$\dot{n}_{out}$:$molar\ flow\ at\ reactor\ outlet$

$\dot{n}_{in}$:$molar\ flow\ at\ reactor\ inlet$

$C_{aromatics}$:$carbon\ atoms\ in\ aromatic\ molecules$

S:$selectivity$

X:$conversion$

The following processes were analyzed: OX-ZEO, combination of FTO catalysts with zeolites and a dual reactor process. The OX-XEO process also includes recent studies with decreased water-gas-shift activity and are labeled with *low* CO_2. The dual reactor approach shows the combination of methanol synthesis with consecutive methanol-to-aromatics (MTA) reaction in separate processes. Additionally, the resulting yields of a combination of methanol synthesis and MTA process that follows dehydrogenation is added. The calculation of the aromatic yields can be found in Table S7 (OX-ZEO), Table S8 (FTO + zeolite) and Table S9 (dual reactor process).

Table S7: reported catalytic performance of bifunctional catalysts for the direct conversion of synthesis gas to aromatics via the OX-ZEO process

catalyst	CO conversion	CO_2 selectivity	aromatics in hydrocarbons	hydrocarbon selectivity	aromatics selectivity	aromatics yield	ref
	%	$\%_C$	$\%_C$	$\%_C$	$\%_C$	$\%_C$	
**OX-ZEO**							
ZrO2	3	34.0	49.0	66.0	32.3	1.0	65
Ce0.2Zr0.8O2/H-ZSM5-40-350	4	28.0	86.0	72.0	61.9	2.5	65
80Ce-ZrO2	4.8	34.0	69.0	66.0	45.5	2.2	65
CeO2	4.8	34.0	59.0	66.0	38.9	1.9	65
20Ce-ZrO2	5.1	34.0	75.0	66.0	49.5	2.5	65
Ce0.2Zr0.8O2/H-ZSM5-40-380	5.5	33.0	83.0	67.0	55.6	3.1	65
40Ce-ZrO2	5.8	34.0	74.0	66.0	48.8	2.8	65
50% ZnCrOx + 50% H-ZSM-5	6.4	49.0	63.9	51.0	32.6	2.1	66
Ce0.2Zr0.8O2/H-ZSM5-40-400	7.5	33.0	77.0	67.0	51.6	3.9	65
20Ce-ZrO2	8	34.0	83.0	66.0	54.8	4.4	65
40Ce-ZrO2	8	34.0	72.0	66.0	47.5	3.8	65
ZnAlOx/H-ZSM-5H	8.5	44	79	56.0	44.2	3.8	67
80Ce-ZrO2	9	34.0	69.0	66.0	45.5	4.1	65
Ce0.2Zr0.8O2/H-ZSM5-40-450	10	35.0	56.0	65.0	36.4	3.6	65
CeO2	11	34.0	58.0	66.0	38.3	4.2	65
50% ZnCrOx + 50% H-ZSM-5	11.2	49.0	70.4	51.0	35.9	4.0	66
MgZrOx/HZSM5-350°C	12.5	17	68.7	83.0	57.0	7.1	68
t-ZrO2/HZSM-5-mix	14.2	33.5	65.0	66.5	43.2	6.1	69
50% ZnCrOx + 50% H-ZSM-5	14.7	49.0	69.8	51.0	35.6	5.2	66
ZnCr2O4-600&H-ZSM-5	14.7	48.0	70.2	52.0	36.5	5.4	70
50% ZnCrOx + 50% H-ZSM-5	15.4	49.0	67.0	51.0	34.2	5.3	66
MgZrOx/HZSM5-400°C	15.5	18	81.7	82.0	67.0	10.4	68
ZnCrO ZSM-5 powder mixing	16.1	43.0	74.0	57.0	42.2	6.8	71
ZnCr2O4/Sbx-H-ZSM-5	17	47.5	83	52.5	43.6	7.4	72
ZnCrO x -ZSM-5-2.8	18.3	49.0	69.0	51.0	35.2	6.4	73
MgZrOx/HZSM5-450°C	20.5	21	60.2	79.0	47.5	9.7	68
Zn-ZrO2/H-ZSM-5	21	42.0	81.0	58.0	47.0	9.9	74
Z0.8C/s-Z5-150	21	36	56.5	64.0	36.2	7.6	75
ZrO2-H&H-ZSM-5	21.6	44.3	52.4	55.7	29.2	6.3	76
Mo-ZrO2/H-ZSM-5	22	42.0	74.0	58.0	42.9	9.4	77
Ce0.2Zr0.8O2/H-ZSM5-40	22.4	34.1	56.3	65.9	37.1	8.3	65
ZnCr2O4-500&H-ZSM-5	23	47.8	73.3	52.2	38.3	8.8	70
ZnCr2O4-400&H-ZSM-5	23.6	46.9	76.0	53.1	40.4	9.5	70
m-ZrO2/HZSM-5-mix	24	36.4	67.4	63.6	42.9	10.3	69
Z0.8C/c-Z5-150	25	35	70	65.0	45.5	11.4	75

Table S7: reported catalytic performance of bifunctional catalysts for the direct conversion of synthesis gas to aromatics via the OX-ZEO process (continued)

catalyst	CO conversion<br>%	CO_2 selectivity<br>$\%_C$	aromatics in hydrocarbons<br>$\%_C$	hydrocarbon selectivity<br>$\%_C$	aromatics selectivity<br>$\%_C$	aromatics yield<br>$\%_C$	ref
Ce0.2Zr0.8O2/H-ZSM5-40	27.8	35.1	57.0	64.9	37.0	10.3	65
Z0.8C/n-Z5-150	28	36.5	62	63.5	39.4	11.0	75
Z0.8C/i-Z5-150	28	36.5	64	63.5	40.6	11.4	75
2.89%Fe-Zn/Cr+ZSM-5	36	45.5	82.5	54.5	45.0	16.2	78
4.48%Fe-Zn/Cr+ZSM-5	45	46.5	81	53.5	43.3	19.5	78
Cr/Zn–Zn/Z5@S1 hybrid	55			100.0	35.7	19.6	79
**low CO2 OX-ZEO**							
ZnO-ZrO2/H-ZSM-5	11	0.0	72.0	100.0	72.0	7.9	80
ZnO-ZrO2/H-ZSM-5	15	5.0	71.0	95.0	67.5	10.1	80
Cr2O3/Mg-ZSM-5@SiO2	17.4	0.0	64.9	100.0	64.9	11.3	81
Cr2O3/La-ZSM-5@SiO2	17.5	0.0	72.2	100.0	72.2	12.6	81
Cr2O3/H-ZSM-5@SiO2-56.1%	17.8	0.0	68.2	100.0	68.2	12.2	81
Cr2O3/H-ZSM-5@SiO2-13.8%	19.5	0.0	68.0	100.0	68.0	13.3	81
Cr2O3/H-ZSM-5@SiO2-39.0%	19.7	0.0	69.3	100.0	69.3	13.7	81
Cr2O3/H-ZSM-5@SiO2	19.7	0.0	69.3	100.0	69.3	13.7	81
Cr2O3/Zn-ZSM-5@SiO2	22.8	0.0	71.4	100.0	71.4	16.3	81
Cr2O3/Ga-ZSM-5@SiO2	24.6	0.0	76.4	100.0	76.4	18.8	81

Table S8: reported catalytic performance of bifunctional catalysts for the direct conversion of synthesis gas to aromatics by combining FTO catalysts and zeolites

catalyst	CO conversion %	CO_2 selectivity $\%_C$	aromatics in hydrocarbons $\%_C$	hydrocarbon selectivity $\%_C$	aromatics selectivity $\%_C$	aromatics yield $\%_C$	ref
**Fe+Z**							
FeMn-HZSM-5	6.7	26.3	36.5	73.7	26.9	1.8	82
CMA||Z-300	17.5	29.9	38.8	70.1	27.2	4.8	83
FeMn-HZSM-5	19.9	35.1	36.5	64.9	23.7	4.7	82
FeMn-HZSM-5	23.1	22.1	39.4	77.9	30.7	7.1	82
CMA||Z-300	23.7	34.0	43.3	66.0	28.6	6.8	83
FeMn-HZSM-5	24.9	34.6	24.2	65.4	15.8	3.9	82
α-Fe2O3-0.75Na/HZSM-5	25.3	41.5	36.2	58.5	21.2	5.4	84
FeNiOx(5:1)-0.41Na/HZSM-5	32.3	47.4	44.8	52.6	23.6	7.6	84
CMA||Z-300	34.9	39.6	55.5	60.4	33.5	11.7	83
CMA||Z-300	35.8	38.5	31.0	61.5	19.1	6.8	83
CMA||Z-300	36.4	37.5	57.0	62.5	35.6	13.0	83
FeMn-HZSM-5	39.9	47.6	43.4	52.4	22.7	9.1	82
FeMnOx(5:1)-0.4Na/HZSM-5	42.1	45.4	28.3	54.6	15.5	6.5	84
FeMn-HZSM-5	44.6	33.7	37.9	66.3	25.1	11.2	82
FeNiOx(5:1)-0.87Na/HZSM-5	46.3	46.6	36.2	53.4	19.3	9.0	84
FeMn-HZSM-5	46.6	42.0	33.9	58.0	19.7	9.2	82
FeNiOx(5:1)-0.87Na/HZSM-5	47.2	46.6	23.4	53.4	12.5	5.9	84
FeMn@MZ5	51.9	36.6	47.1	63.4	29.9	15.5	85
Fe10Mn1KSi-Hol HZSM-5 (27)	53.4	49.4	33.8	50.6	17.1	9.1	86
Fe1Mn0.5@MZ5-(89)	57	38.0	59.0	62.0	36.6	20.9	85
FeMn-HZSM-5	60.4	42.8	34.1	57.2	19.5	11.8	82
FeMn-HZSM-5	60.4	42.8	34.1	57.2	19.5	11.8	82
CMA||Z-300	68.9	41.6	59.1	58.4	34.5	23.8	83
FeMn-HZSM-5	69.9	45.5	32.4	54.5	17.7	12.3	82
FeMnK/SiO2+HZSM-5 powder mix.	74	47.0	29.0	53.0	15.4	11.4	87
CMA/Hol-Z5-N@S1	75	41	61	59.0	36.0	27.0	88
FeMnK/SiO2+HZSM-5 dual bed	77	48.0	23.0	52.0	12.0	9.2	87
FeMn-HZSM-5	79.1	43.7	38.0	56.3	21.4	16.9	82
FeMn-HZSM-5	81.1	40.9	40.7	59.1	24.1	19.5	82
Fe10Mn0KSi-Hol HZSM-5 (27)	82.5	47.5	33.5	52.5	17.6	14.5	86
3Fe:1Cu:0.5Co/HZ, calc 700°C	83	32.0	37.0	68.0	25.2	20.9	89
Fe10Mn5KSi-Hol HZSM-5 (27)	83.8	46.8	37.7	53.2	20.0	16.8	86
FeMnK/SiO2+HZSM-5 gran. mix.	84	47.0	26.0	53.0	13.8	11.6	87
FeMnOx(5:1)-0.4Na/HZSM-5	84.1	45.4	15.7	54.6	8.6	7.2	84
Fe10Mn10KSi-Hol HZSM-5 (27)	85.9	47.1	38.2	52.9	20.2	17.3	86

Table S8: reported catalytic performance of bifunctional catalysts for the direct conversion of synthesis gas to aromatics by combining FTO catalysts and zeolites (continued)

catalyst	CO conversion %	CO_2 selectivity $\%_C$	aromatics in hydrocarbons $\%_C$	hydrocarbon selectivity $\%_C$	aromatics selectivity $\%_C$	aromatics yield $\%_C$	ref
FeMn-HZSM-5	86.7	41.2	34.2	58.8	20.1	17.4	82
FeMn-HZSM-5	86.8	46.9	24.0	53.1	12.7	11.1	82
3Fe:1Cu:0.5Co/HZ, 3500 h-1	88	34.0	28.0	66.0	18.5	16.3	89
FeZnNa@0.6-HZSM-5-a	88.8	27.5	50.6	72.5	36.7	32.6	90
FeZnNa@0.6-HZSM-5	89.2	26.9	40.5	73.1	29.6	26.4	90
3Fe:1Cu:0.5Co/HZ, calc 350°C	90	26.0	40.0	74.0	29.6	26.6	89
3Fe:2Cu/HZ	92.5	32.0	38.0	68.0	25.8	23.9	89
3Fe:1Cu:0.5Co/HZ, 2 Mpa	92.5	16.0	30.0	84.0	25.2	23.3	89
3Fe:2Cu:0.5Co/HZ	93	30.0	39.0	70.0	27.3	25.4	89
3Fe:1Cu:0.5Co/HZ, calc 400°C	93	25.0	44.0	75.0	33.0	30.7	89
3Fe:1Cu:0.5Co/HZ, calc 600°C	93	25.0	45.0	75.0	33.8	31.4	89
3Fe:1Cu:0.5Co/HZ, 2500 h-1	93	26.0	43.0	74.0	31.8	29.6	89
FeMnOx(5:1)-0.4Na/HZSM-5	93.7	45.3	26.0	54.7	14.2	13.3	84
3Fe:1Cu:0.5Co/HZ, 320°C	94	23.0	40.0	77.0	30.8	29.0	89
3Fe:1Cu:0.5Co/HZ, 1000 h-1	94	17.0	40.0	83.0	33.2	31.2	89
3Fe:1Cu:0.5Co/HZ, calc 450°C	95	26.0	46.0	74.0	34.0	32.3	89
3Fe:1Cu:0.5Co/HZ, H2/CO=1	95	29.0	43.0	71.0	30.5	29.0	89
3Fe:1Cu:0.5Co/HZ, 3 Mpa	95	18.0	43.0	82.0	35.3	33.5	89
Fe/HZ	96	36.0	31.0	64.0	19.8	19.0	89
3Fe:1Cu:0.5Co/HZ, 330°C	96	22.0	45.0	78.0	35.1	33.7	89
KF80M	96.4	36.9	34.1	63.1	21.5	20.7	91
3Fe:1Cu:0.5Co/HZ	97	23.0	53.0	77.0	40.8	39.6	89
KF60M	97	32.8	39.8	67.2	26.7	25.9	91
3Fe:0.5Co/HZ	97.5	27.0	41.0	73.0	29.9	29.2	89
3Fe:1Cu/HZ	97.5	29.0	40.0	71.0	28.4	27.7	89
3Fe:1Cu:0.5Co/HZ, H2/CO=2	97.5	18.0	44.0	82.0	36.1	35.2	89
3Fe:1Cu:0.5Co/HZ, H2/CO=3	97.5	16.0	30.0	84.0	25.2	24.6	89
KF40M	97.6	32.1	36.2	67.9	24.6	24.0	91
KF20M	97.7	31.4	34.4	68.6	23.6	23.1	91
3Fe:1Co/HZ	98	25.0	38.0	75.0	28.5	27.9	89
3Fe:1Cu:1Co/HZ	98	24.0	45.0	76.0	34.2	33.5	89
3Fe:1Cu:0.5Co/HZ, 360°C	98	26.0	40.0	74.0	29.6	29.0	89
3Fe:1Cu:0.5Co/HZ, 5 Mpa	98	31.0	37.0	69.0	25.5	25.0	89
3Fe:1Cu:0.5Co/HZ, 350°C	98.5	23.0	45.0	77.0	34.7	34.1	89
0.2Cu-Fe/Z5	99	41	37.5	59.0	22.1	21.9	92
0.7Cu-Fe/Z5	99	41	39	59.0	23.0	22.8	92

Table S8: reported catalytic performance of bifunctional catalysts for the direct conversion of synthesis gas to aromatics by combining FTO catalysts and zeolites (continued)

catalyst	CO conversion<br>%	CO_2 selectivity<br>$\%_C$	aromatics in hydrocarbons<br>$\%_C$	hydrocarbon selectivity<br>$\%_C$	aromatics selectivity<br>$\%_C$	aromatics yield<br>$\%_C$	ref
1.5Cu-Fe/Z5	99	39	43	61.0	26.2	26.0	92

Table S9: combined reported catalytic performance of catalysts for the conversion of synthesis gas to aromatics via a dual reactor process

catalyst	CO conversion	CO_2 selectivity	methanol selectivity	aromatics selectivity from methanol	aromatics selectivity from synthesis gas	yield	ref
	%	$\%_C$	$\%_C$	$\%_C$	$\%_C$	$\%_C$	
**dual reactor process**							
**MeOH**							
Cu/ZnO/Al2O3	8.6			97.7		8.4	30
2Cu_MCF 10.7	10.7			97.0		10.4	31
Cu/ZnO/Al2O3	29.9			99.6		29.8	30
Cu/ZnO/Al2O3	34.4			99.8		34.3	30
Cu/ZnO/Al2O3	40.3			98.7		39.8	30
Cu/ZnO/Al2O3	47.0			98.9		46.5	30
**MeOH + MTA**							
H-ZSM-5	8.6	0.0	33.0	33.0	32.2	2.8	93
8% Ga/ZSM-5	8.6	0.0	50.0	50.0	48.9	4.2	94
Gd-ZSM-5	8.6	0.0	35.0	35.0	34.2	2.9	95
Zn-ZSM-5	8.6	0.0	46.0	46.0	44.9	3.9	96
Zn-ZSM-5	8.6	0.0	41.0	41.0	40.1	3.4	96
H-ZSM-5	10.7	0.0	33.0	33.0	32.0	3.4	93
8% Ga/ZSM-5	10.7		50.0	50.0	48.5	5.2	94
Gd-ZSM-5	10.7	0.0	35.0	35.0	34.0	3.6	95
Zn-ZSM-5	10.7	0.0	46.0	46.0	44.6	4.8	96
Zn-ZSM-5	10.7	0.0	41.0	41.0	39.8	4.3	96
H-ZSM-5	29.9	0.0	33.0	33.0	32.9	9.8	93
8% Ga/ZSM-5	29.9	0.0	50.0	50.0	49.8	14.9	94
Gd-ZSM-5	29.9	0.0	35.0	35.0	34.9	10.4	95
Zn-ZSM-5	29.9	0.0	46.0	46.0	45.8	13.7	96
Zn-ZSM-5	29.9	0.0	41.0	41.0	40.8	12.2	96
H-ZSM-5	34.4	0.0	33.0	33.0	32.9	11.3	93
8% Ga/ZSM-5	34.4	0.0	50.0	50.0	49.9	17.2	94
Gd-ZSM-5	34.4	0.0	35.0	35.0	34.9	12.0	95
Zn-ZSM-5	34.4	0.0	46.0	46.0	45.9	15.8	96
Zn-ZSM-5	34.4	0.0	41.0	41.0	40.9	14.1	96
H-ZSM-5	40.3	0.0	33.0	33.0	32.6	13.1	93
8% Ga/ZSM-5	40.3	0.0	50.0	50.0	49.4	19.9	94
Gd-ZSM-5	40.3	0.0	35.0	35.0	34.5	13.9	95
Zn-ZSM-5	40.3	0.0	46.0	46.0	45.4	18.3	96
Zn-ZSM-5	40.3	0.0	41.0	41.0	40.5	16.3	96
H-ZSM-5	47.0	0.0	33.0	33.0	32.6	15.3	93

Table S9: combined reported catalytic performance of catalysts for the conversion of synthesis gas to aromatics via a dual reactor process (continued)

catalyst	CO conversion %	CO_2 selectivity $\%_C$	methanol selectivity $\%_C$	aromatics selectivity from methanol $\%_C$	aromatics selectivity from synthesis gas $\%_C$	yield $\%_C$	ref
8% Ga/ZSM-5	47.0	0.0	50.0	50.0	49.5	23.2	94
Gd-ZSM-5	47.0	0.0	35.0	35.0	34.6	16.3	95
Zn-ZSM-5	47.0	0.0	46.0	46.0	45.5	21.4	96
Zn-ZSM-5	47.0	0.0	41.0	41.0	40.5	19.1	96
**MTA via dehydrogenation**							
Zn/ZSM-5	8.6		95.8	95.8	93.6	8.1	97
Zn/ZSM-5	10.7		95.8	95.8	92.9	9.9	97
Zn/ZSM-5	29.9		95.8	95.8	95.4	28.5	97
Zn/ZSM-5	34.4		95.8	95.8	95.6	32.9	97
Zn/ZSM-5	40.3		95.8	95.8	94.6	38.1	97
Zn/ZSM-5	47.0		95.8	95.8	94.8	44.5	97

4. Gasoline

We analyzed recent publications of bifunctional catalysis to convert synthesis gas directly to gasoline. Beside the overall selectivity of the bifunctional process, we also focused on the resulting octane number of the C_5-C_{11} products.

4.1. Octane number

The octane number of the C_5-C_{11} products was estimated by using the blending research octane number (BRON) of the single components. The BRON can describe the effect of a single component being blended into a base gasoline fuel, whereas the pure research octane number (RON) of a component is measured as pure compound [98]. The BRON of the C_5-C_{11} paraffins, iso-paraffins, olefins, iso-olefins and aromatics were either found in literature [98–100] or estimated by extrapolation.

The average C_5-C_{11} paraffins BRON can be found in Table S10 and Figure S1. The individual BRON of all isomers were averaged for every carbon number with the same number of branches. Analog, the average BRON for olefins were determined (Table S11 and Figure S2). However, the olefins were not further divided by the position of the double bond, despite the effect of the double bond position on the BRON (Figure S3). The BRON of C6-C11 aromatics was averaged over the corresponding carbon numbers (Table S12, Figure S4 and Figure S5).

Table S10: average blending research octane numbers of C_5-C_{11} paraffins divided into number of branches.

	number of branches					
	0	1	2	3	4	5
**C_5**	62	99	100			
**C_6**	19	85	93			
**C_7**	0	54	84	113		
**C_8**	-19	31	69	101	120	
**C_9**	-30	20[1]	56[1]	92	121	
**C_{10}**	-41	2	34	70[1]	111	
**C_{11}**	-48[1]	-12[1]	18[1]	55[1]	98[1]	130[1]

[1]: extrapolated

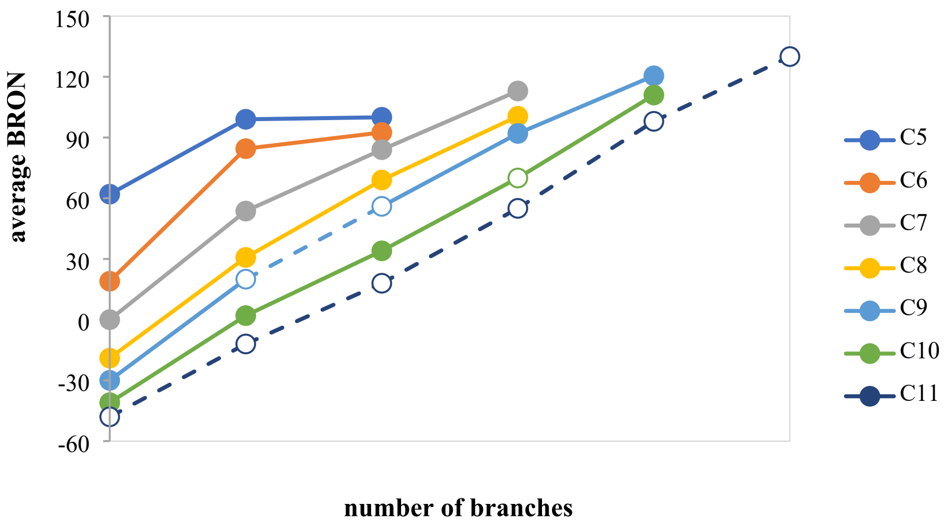

Figure S1: average blending research octane number of C_5-C_{11} paraffins as function of number of branching.

Table S11: average blending research octane numbers of C_5-C_{11} olefins divided into number of branches.

	number of branches					
	0	1	2	3	4	5
C_5	112	125	127			
C_6	100	112	120			
C_7	75	86	98	110[1]		
C_8	61	72[1]	85[1]	100[1]	115[1]	
C_9	48	60[1]	72[1]	87[1]	102[1]	
C_{10}	35	47[1]	59[1]	75[1]	90[1]	
C_{11}	20[1]	32[1]	46[1]	63[1]	78[1]	90[1]

[1]: extrapolated

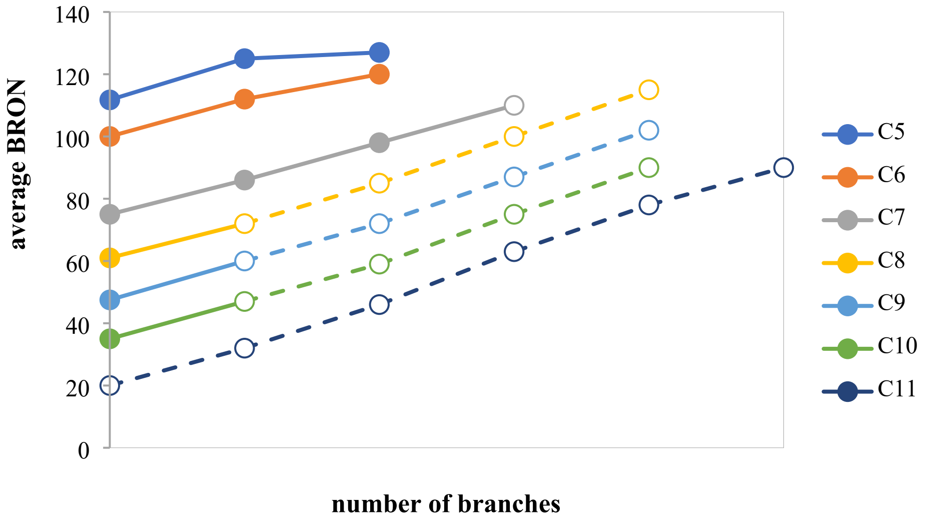

Figure S2: average blending research octane number of C_5-C_{11} olefins as function of number of branching.

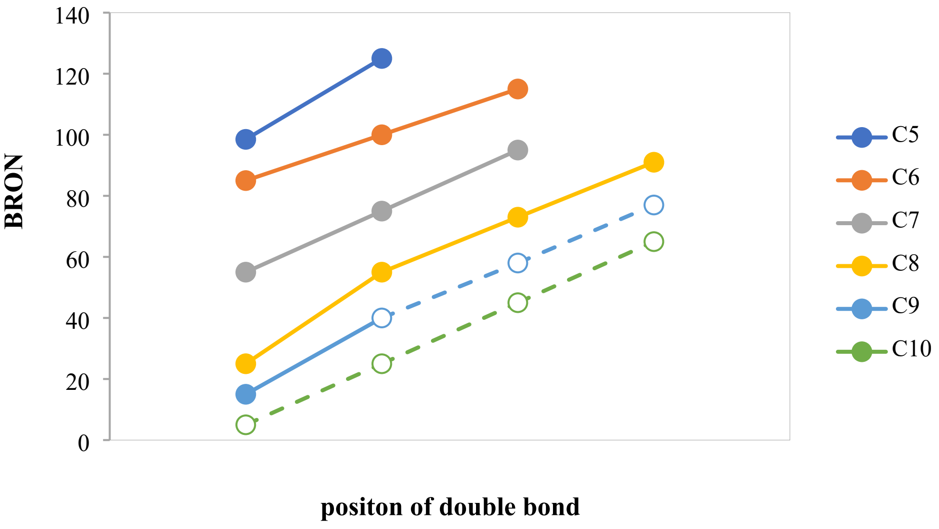

Figure S3: blending research octane number of linear C_5-C_{10} olefins as function of double bond position.

Table S12: average blending research octane numbers of C_6-C_{11} aromatics divided into number of side chains.

	side chains						
	0	1	2	3	4	5	average
**C_6**	108						108
**C_7**		120					120
**C_8**		120.9	131.5				126
**C_9**		124.1	127[1]	131[1]			127
**C_{10}**		116.7	121.8	126.9	133		125
**C_{11}**		101	112.7	120[1]	125[1]	127[1]	117

[1]: extrapolated

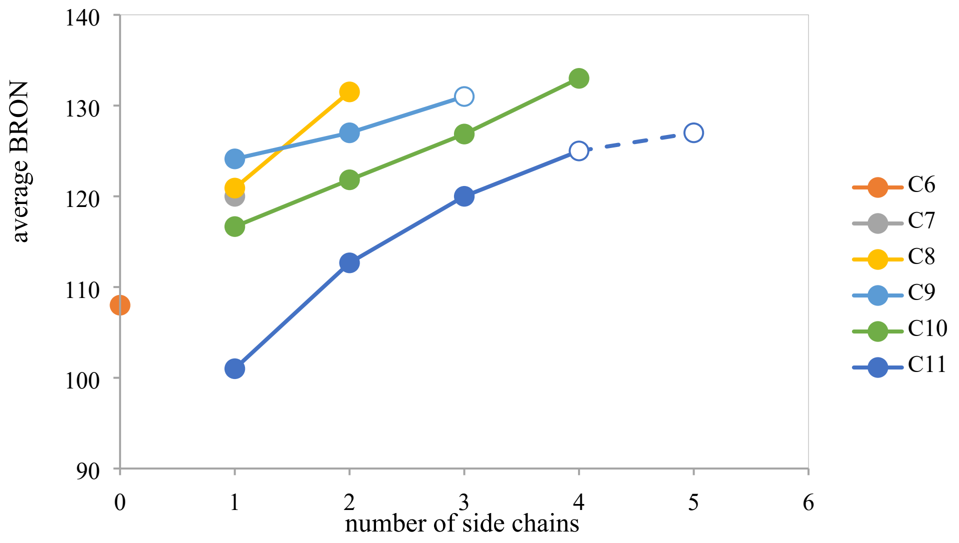

Figure S4: average blending research octane number of C_6-C_{11} aromatics as function of number of side chains.

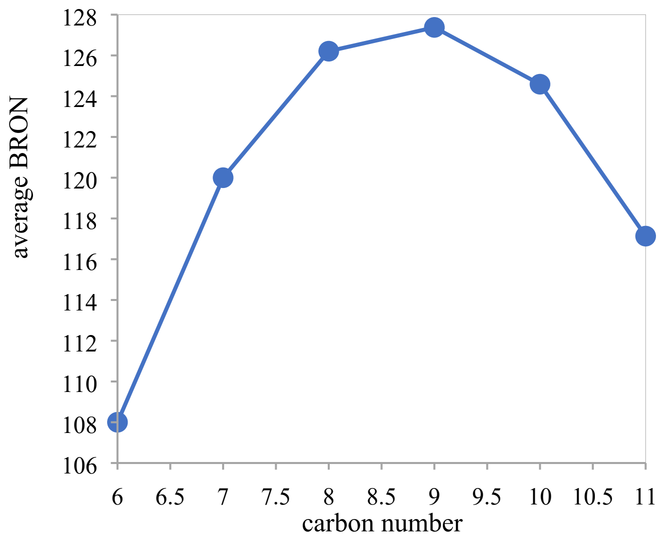

Figure S5: average blending research octane number of aromatics as function of carbon number.

4.2. Analysis of published literature

The overall selectivity of the conversion of synthesis gas to gasoline was analyzed analog to the selectivity of C_2-C_4 olefins (Equation 10 and Equation 11). Here, paraffins, olefins (both including isomers) and aromatics in the range of C_5-C_{11} were considered.

$$Y(gasoline) = \frac{n_{out}(C_{gasoline})}{n_{in}(CO_x)} \quad \text{Equation 10}$$

$$S(gasoline) = \frac{Y(gasoline)}{X(CO_x)} \quad \text{Equation 11}$$

Where,

Y: $yield$

$\dot{n}_{out}$: $molar\ flow\ at\ reactor\ outlet$

$\dot{n}_{in}$: $molar\ flow\ at\ reactor\ inlet$

$C_{gasoline}$: $carbon\ atoms\ in\ the\ C_5 - C_{11}\ fraction$

S: $selectivity$

X: $conversion$

To estimate the octane number of the C_5-C_{11} products the reported selectivities of C_5-C_{11} paraffins, iso-paraffins, olefins, iso-olefins and aromatics were normalized. Isomers (if not reported in detail) were further divided by the number of branches according to the thermodynamic equilibrium at the corresponding reaction temperature. If the fraction of isomers was not reported for paraffins or olefins, the linear components were considered as well (Table S13). The individual concentrations of paraffins, iso-paraffins, olefins, iso-olefins and aromatics were multiplied with the corresponding BON (Table S10 - Table S12) and added up, resulting in the overall octane number of the C_5-C_{11} products. If the concentration of olefins exceeded the allowed amount of 18%, we reduced the concentration of olefins in favor of additional paraffins. Also, when *iso*-paraffins and olefins were reported as a single group we divided the corresponding concentration to olefins and *iso*-paraffins accordingly.

We analyzed recent publications with the following approaches to convert synthesis gas to gasoline: combination of Co-based FT catalysts with zeolite, whereas we distinguished between 12-membered ring (Table S14) and 10-membered ring zeolites (Table S15) and non-micro-porous solid acids (NMPA, Table S16). The combination of iron-based FT catalysts and zeolites (Table S17), the OX-ZEO process (Table S18) were analyzed. Additionally, dual bed configurations with dedicated temperatures for the individual catalyst beds were investigated (Table S19). Finally, the dual reactor approach shows the combination of methanol synthesis with consecutive methanol-to-gasoline (MTG) reaction in separate processes was added as a comparison (Table 20).

These calculations of the octane number of the C_5-C_{11} products are theoretical and based on several assumptions, estimations, and simplifications. To determine the real RON, the mixture of condensed products must be analyzed using validated methods, such as ASTM D2699, GB/T 5487. However, this estimation can give a good indication of the real RON of the corresponding products.

Table S13: thermodynamic distribution of C_5-C_{11} *n*- and *iso*-paraffins between 200°C and 300°C. Calculated with Outotec HSC 4 at 20 bar pressure.

	**temperature**																				
	**200°C**	**205°C**	**210°C**	**215°C**	**220°C**	**225°C**	**230°C**	**235°C**	**240°C**	**245°C**	**250°C**	**255°C**	**260°C**	**265°C**	**270°C**	**275°C**	**280°C**	**285°C**	**290°C**	**295°C**	**300°C**
C5 lin	20.0%	20.5%	21.0%	21.4%	21.9%	22.4%	22.8%	23.3%	23.7%	24.1%	24.6%	25.0%	25.4%	25.8%	26.2%	26.6%	27.0%	27.4%	27.8%	28.1%	28.5%
C5 mono	53.3%	53.6%	53.9%	54.2%	54.5%	54.7%	54.9%	55.1%	55.3%	55.5%	55.6%	55.8%	55.9%	56.0%	56.0%	56.1%	56.2%	56.2%	56.3%	56.3%	56.3%
C5 di	26.7%	25.9%	25.1%	24.3%	23.6%	22.9%	22.2%	21.6%	21.0%	20.4%	19.8%	19.3%	18.7%	18.2%	17.7%	17.3%	16.8%	16.4%	16.0%	15.6%	15.2%
C6 lin	11.0%	11.3%	11.6%	11.9%	12.2%	12.5%	12.8%	13.1%	13.4%	13.7%	13.9%	14.2%	14.5%	14.8%	15.0%	15.3%	15.5%	15.8%	16.1%	16.3%	16.6%
C6 mono	46.5%	47.0%	47.4%	47.9%	48.3%	48.7%	49.1%	49.4%	49.8%	50.1%	50.5%	50.8%	51.1%	51.4%	51.7%	51.9%	52.2%	52.4%	52.7%	52.9%	53.1%
C6 di	42.5%	41.8%	41.0%	40.2%	39.5%	38.8%	38.1%	37.5%	36.8%	36.2%	35.6%	35.0%	34.4%	33.9%	33.3%	32.8%	32.3%	31.8%	31.3%	30.8%	30.3%
C7 lin	10.6%	10.9%	11.1%	11.3%	11.5%	11.8%	12.0%	12.2%	12.4%	12.6%	12.8%	13.0%	13.2%	13.4%	13.6%	13.8%	13.9%	14.1%	14.3%	14.5%	14.7%
C7 mono	46.8%	47.2%	47.6%	47.9%	48.2%	48.6%	48.9%	49.2%	49.4%	49.7%	50.0%	50.2%	50.4%	50.7%	50.9%	51.1%	51.3%	51.5%	51.7%	51.8%	52.0%
C7 di	38.5%	37.9%	37.5%	37.0%	36.5%	36.1%	35.6%	35.2%	34.8%	34.4%	34.0%	33.6%	33.2%	32.9%	32.5%	32.2%	31.9%	31.6%	31.3%	30.9%	30.7%
C7 tri	4.1%	4.0%	3.9%	3.8%	3.7%	3.6%	3.6%	3.5%	3.4%	3.3%	3.3%	3.2%	3.1%	3.1%	3.0%	2.9%	2.9%	2.8%	2.8%	2.7%	2.7%
C8 lin	7.7%	7.8%	7.9%	8.1%	8.2%	8.4%	8.5%	8.6%	8.8%	8.9%	9.0%	9.2%	9.3%	9.4%	9.6%	9.7%	9.8%	9.9%	10.0%	10.2%	10.3%
C8 mono	42.4%	42.7%	43.0%	43.3%	43.5%	43.8%	44.1%	44.3%	44.6%	44.8%	45.0%	45.2%	45.4%	45.6%	45.8%	46.0%	46.2%	46.4%	46.6%	46.7%	46.9%
C8 di	45.9%	45.5%	45.1%	44.7%	44.3%	44.0%	43.6%	43.3%	42.9%	42.6%	42.3%	42.0%	41.7%	41.4%	41.1%	40.8%	40.5%	40.3%	40.0%	39.8%	39.5%
C8 tri	4.0%	4.0%	3.9%	3.9%	3.8%	3.8%	3.8%	3.7%	3.7%	3.6%	3.6%	3.6%	3.5%	3.5%	3.5%	3.4%	3.4%	3.4%	3.3%	3.3%	3.3%
C8 quad	0.1%	0.1%	0.1%	0.1%	0.1%	0.1%	0.1%	0.1%	0.1%	0.1%	0.1%	0.0%	0.0%	0.0%	0.0%	0.0%	0.0%	0.0%	0.0%	0.0%	0.0%
C9 lin	8.9%	9.0%	9.2%	9.3%	9.5%	9.6%	9.8%	9.9%	10.1%	10.2%	10.4%	10.5%	10.6%	10.8%	10.9%	11.1%	11.2%	11.3%	11.4%	11.6%	11.7%
C9 mono	66.5%	66.6%	66.8%	66.9%	67.0%	67.1%	67.3%	67.4%	67.5%	67.6%	67.6%	67.7%	67.8%	67.9%	67.9%	68.0%	68.0%	68.1%	68.1%	68.2%	68.2%
C9 di	21.1%	20.8%	20.5%	20.2%	20.0%	19.7%	19.5%	19.2%	19.0%	18.8%	18.6%	18.4%	18.2%	18.0%	17.8%	17.6%	17.5%	17.3%	17.1%	17.0%	16.8%
C9 tri	3.5%	3.5%	3.5%	3.5%	3.4%	3.4%	3.4%	3.4%	3.4%	3.3%	3.3%	3.3%	3.3%	3.3%	3.3%	3.3%	3.2%	3.2%	3.2%	3.2%	3.2%
C9 quad	0.1%	0.1%	0.1%	0.1%	0.1%	0.1%	0.1%	0.1%	0.1%	0.1%	0.1%	0.1%	0.1%	0.1%	0.1%	0.1%	0.1%	0.1%	0.1%	0.1%	0.1%
C10 lin	7.4%	7.6%	7.7%	7.9%	8.1%	8.2%	8.4%	8.5%	8.7%	8.8%	9.0%	9.1%	9.3%	9.4%	9.6%	9.7%	9.9%	10.0%	10.1%	10.3%	10.4%
C10 mono	50.5%	50.9%	51.2%	51.5%	51.8%	52.1%	52.4%	52.7%	53.0%	53.2%	53.5%	53.7%	53.9%	54.2%	54.4%	54.6%	54.8%	55.0%	55.2%	55.3%	55.5%
C10 di	38.1%	37.6%	37.1%	36.6%	36.1%	35.7%	35.2%	34.8%	34.4%	34.0%	33.6%	33.2%	32.8%	32.5%	32.1%	31.8%	31.4%	31.1%	30.8%	30.5%	30.1%
C10 tri	3.8%	3.8%	3.8%	3.7%	3.7%	3.7%	3.7%	3.7%	3.7%	3.7%	3.7%	3.7%	3.7%	3.7%	3.7%	3.7%	3.7%	3.7%	3.7%	3.7%	3.7%
C10 quad	0.2%	0.2%	0.2%	0.2%	0.2%	0.2%	0.2%	0.2%	0.2%	0.2%	0.2%	0.2%	0.2%	0.2%	0.2%	0.2%	0.2%	0.3%	0.3%	0.3%	0.3%
C11 lin	9.3%	9.5%	9.7%	9.9%	10.0%	10.2%	10.4%	10.5%	10.7%	10.8%	11.0%	11.1%	11.3%	11.4%	11.6%	11.7%	11.9%	12.0%	12.1%	12.3%	12.4%
C11 mono	58.9%	59.2%	59.5%	59.7%	60.0%	60.2%	60.5%	60.7%	60.9%	61.1%	61.3%	61.4%	61.6%	61.8%	61.9%	62.1%	62.2%	62.4%	62.5%	62.6%	62.7%
C11 di	29.2%	28.8%	28.3%	27.9%	27.5%	27.1%	26.7%	26.4%	26.0%	25.7%	25.3%	25.0%	24.7%	24.4%	24.1%	23.8%	23.6%	23.3%	23.1%	22.8%	22.6%
C11 tri	2.5%	2.4%	2.4%	2.4%	2.4%	2.4%	2.4%	2.4%	2.4%	2.3%	2.3%	2.3%	2.3%	2.3%	2.3%	2.3%	2.3%	2.3%	2.2%	2.2%	2.2%
C11 quad	0.1%	0.1%	0.1%	0.1%	0.1%	0.1%	0.1%	0.1%	0.1%	0.1%	0.1%	0.1%	0.1%	0.1%	0.1%	0.1%	0.1%	0.1%	0.1%	0.1%	0.1%

Table S14: reported catalytic performance of bifunctional catalysts for the direct conversion of synthesis gas to gasoline by combining Co-based FT catalysts and 12-membered ring zeolites

catalyst	zeolite	temperature	pressure	CO conversion	CO_2 selectivity	hydrocarbon distribution		C_5-C_{11} yield	C_5-C_{11}				ref
						CH_4	C_5-C_{11}		lin paraffins	iso-paraffins	olefins	octane number	
		°C	bar(g)	%	$\%_C$	$\%_C$	$\%_C$	$\%_C$	$\%_C$	$\%_C$	$\%_C$		
Co/USY-S	USY	260	10	50	0	28	39.4	19.7	29.6	41.5	28.9	64.7	101
									34.1	47.9	18	60.0	
									41.6	58.4	0	52.3	
Co/Y-Ce	Y	250	20	34	2	11	73.5	24.5	28.6	71.4	0	29.3	102
									28.6	53.4	18	34.4	
									28.6	0	71.4	49.6	
Co/Y-La	Y	250	20	40	2	9.5	54.5	21.4	26.6	73.4	0	19.6	102
									26.6	55.4	18	25.2	
									26.6	0	73.4	42.3	
Co/Y-P	Y	260	10	50.2	1.1	21.9	59.4	29.5	27	44	29	44.2	103
									31.2	50.8	18	35.6	
									38	62	0	21.4	
Co/Y-A	Y	260	10	66.2	1.5	10.8	69.5	45.3	28.5	64.1	7.4	48.2	103
									29.7	66.8	3.5	46.7	
									30.8	69.2	0	43.1	
Co/Y-B	Y	260	10	69.7	2.9	11.9	65.3	44.2	31	56.9	12.1	49.8	103
									33.1	60.9	6	47.4	
									35.2	64.8	0	41.8	
Co/Y-AB0.25	Y	260	10	66.3	1.9	14.7	67.3	43.8	26.9	46.4	26.7	47.1	103
									30.1	51.9	18	40.4	
									36.7	63.3	0	30.6	
Co/Y-AB1	Y	260	10	75.7	3.5	11.4	66.8	48.8	24.9	51.3	23.8	44.9	103
									26.8	55.2	18	37.6	
									32.7	67.3	0	28.0	
Co/Y-AB4	Y	260	10	75.9	1.8	8.4	71.5	53.3	15	61.2	23.8	49.7	103
									16.1	65.9	18	42.7	
									19.7	80.3	0	34.1	
Co/Y-AB6	Y	260	10	66.5	2	14.5	64.3	41.9	28.3	54.5	17.1	44.1	103
									31.2	60.2	8.6	40.0	
									34.2	65.8	0	31.6	
Co/MOR	MOR	250	20	39.7	0.6	9.2	18.1	7.1	61	29.3	9.7	22.6	104
									64.2	30.9	4.9	19.1	
									67.5	32.5	0	15.6	
Co/BEA	BEA	250	20	17.5	0.7	10.5	18.7	3.2	56	37.5	6.5	27.4	104

Table S14: reported catalytic performance of bifunctional catalysts for the direct conversion of synthesis gas to gasoline by combining Co-based FT catalysts and 12-membered ring zeolites (continued)

catalyst	zeolite	temperature	pressure	CO conversion	CO_2 selectivity	hydrocarbon distribution			C_5-C_{11}				ref
						CH_4	C_5-C_{11}	C_5-C_{11} yield	lin paraffins	iso-paraffins	olefins	octane number	
		°C	bar(g)	%	$\%_C$	$\%_C$	$\%_C$	$\%_C$	$\%_C$	$\%_C$	$\%_C$		
									57.9	38.8	3.3	25.1	
									59.9	40.1	0	22.7	

Table S15: reported catalytic performance of bifunctional catalysts for the direct conversion of synthesis gas to gasoline by combining Co-based FT catalysts and 10-membered ring zeolites

catalyst	zeolite	temperature	pressure	CO conversion	CO2 selectivity	hydrocarbon distribution			C5-C11				ref
						CH4	C5-C11	C5-C11 yield	lin paraffins	iso-paraffins	olefins	octane number	
		°C	bar(g)	%	$\%_C$	$\%_C$	$\%_C$	$\%_C$	$\%_C$	$\%_C$	$\%_C$		
Z/Co/SiO2	ZSM5	260	10	83	4	21	39.7	31.7	70.6	29.4	0	28.8	
									70.6	11.4	18	33.5	105
									70.6	0	29.4	36.5	
Co/SiO2+ZSM5	ZSM5	260	10	82	15	13.5	40.4	28.2	55.3	44.7	0	30.3	
									55.3	26.7	18	35.1	105
									55.3	0	44.7	42.2	
Z/Co/SiO2-crushed	ZSM5	260	10.0	81	7	19.5	42.1	31.7	66.1	33.9	0	31.5	
									66.1	15.9	18	36.2	105
									66.1	0	33.9	40.4	
Z/Co/SiO2-no TEOS	ZSM5	260	10	90	12	21.5	37.8	30.0	70.1	29.9	0	28.7	
									70.1	11.9	18	33.5	105
									70.1	0	29.9	36.6	
Z/Co/SiO2	ZSM5	260	10.0	34	9	11.5	53.6	16.6	61.4	38.6	0	18.1	
									61.4	20.6	18	22.9	105
									61.4	0	38.6	28.5	
Co/ZSM5	ZSM5	240	15	31	1	19	43.6	13.4	65.7	34.3	0	17.4	
									65.7	16.3	18	22.3	105
									65.7	0	34.3	26.6	
Co/meso-ZSM5	ZSM5	240	15.0	80	3	19	46.6	36.2	47.3	52.7	0	28.1	
									47.3	34.7	18	33.1	105
									47.3	0	52.7	43.0	
ZSM-5/Co-Al2O3/M	ZSM5	230	12	78.7		10.9	*89.0*	*70.0*	25.7	50.9	23.3	38.2	
							in liquid products	*in liquid products*	27.5	54.5	18	34.8	106
									33.6	66.4	0	23.7	
ZSM-5/Co-Al2O3/M	ZSM5	250	12	78.9		17.2	*91.4*	*72.1*	26.1	53	20.8	36.5	
							in liquid products	*in liquid products*	27.1	54.9	18	34.7	106
									33	67	0	23.1	
ZSM-5/Co-Al2O3/M	ZSM5	230	6	81.6		17.3	*92.1*	*75.2*	24.6	49.3	26.1	40.5	
							in liquid products	*in liquid products*	27.3	54.7	18	35.3	106
									33.3	66.7	0	23.7	
ZSM-5/Co-Al2O3/M	ZSM5	230	20	63.2		10.2	*72.9*	*46.1*	28.8	46.2	25.1	28.5	
							in liquid products	*in liquid products*	31.5	50.5	18	23.9	106
									38.4	61.6	0	12.2	
Co/MZ	meso ZSM5	260	10	25.9	0	17.6	65.5	17.0	23.6	48.6	27.7	54.1	107

Table S15: reported catalytic performance of bifunctional catalysts for the direct conversion of synthesis gas to gasoline by combining Co-based FT catalysts and 10-membered ring zeolites (continued)

catalyst	zeolite	temperature	pressure	CO conversion	CO2 selectivity	hydrocarbon distribution		C5-C11 yield	C5-C11				ref
						CH4	C5-C11		lin paraffins	iso-paraffins	olefins	octane number	
		°C	bar(g)	%	$\%_C$	$\%_C$	$\%_C$	$\%_C$	$\%_C$	$\%_C$	$\%_C$		
									26.8	55.2	18	48.0	
									32.7	67.3	0	36.8	
Co/Z	ZSM5	250	20	22	0.99	29.9	44.3	9.7	22.2	77.8	0	53.7	
									22.2	59.8	18	58.4	108
									22.2	0	77.8	74.1	
Co/M-4Z	ZSM5	250	20	6.9	1.08	26.5	44.8	3.1	20.7	79.3	0	67.8	
									20.7	61.3	18	72.4	108
									20.7	0	79.3	87.9	
Co/M-Z	ZSM5	250	20	22.2	0.96	18.7	54.0	11.9	37.1	62.9	0	35.4	
									37.1	44.9	18	40.5	108
									37.1	0	62.9	53.2	
4Co/M-Z	ZSM5	250	20	40.2	0.63	15.6	40.1	16.0	56.5	43.5	0	16.8	
									56.5	25.5	18	22.1	108
									56.5	0	43.5	29.7	
Co-4.5/Z5	ZSM5	240	20	18	0	28.3	41.9	7.5	52.5	18.1	29.4	40.9	
									61	21	18	31.8	109
									74.4	25.6	0	17.3	
Co-9.9/Z5	ZSM5	240	20	59	0	21.9	44.4	26.2	69.9	15	15	19.2	
									76.1	16.4	7.5	14.5	109
									82.3	17.7	0	9.8	
Co-14/Z5	ZSM5	240	20	58	0	20.9	45.1	26.1	72.9	11.3	15.8	8.4	
									79.8	12.4	7.9	3.4	109
									86.6	13.4	0	-1.6	
Co-18/Z5	ZSM5	240	20	50	0	21	40.3	20.1	73.2	11.8	15	9.8	
									79.7	12.8	7.5	4.8	109
									86.2	13.8	0	-0.2	
Co/ZSM-5	ZSM5	250	20	26.8	0.5	11.7	23.9	6.4	20	56.7	23.2	50.2	
									21.4	60.6	18	47.6	104
									26.1	73.9	0	38.6	
Co-SiO2/ZSM-5/Al2O3	ZSM5	250	10	75.2	2.2	15.6	39.9	29.3	18.3	22.5	59.2	51.7	
									36.8	45.2	18	27.7	110
									44.9	55.1	0	17.3	

Table S16: reported catalytic performance of bifunctional catalysts for the direct conversion of synthesis gas to gasoline by combining Co-based FT catalysts and non-micro porous solid acids

catalyst	zeolite	temperature	pressure	CO conversion	CO_2 selectivity	hydrocarbon distribution			C_5-C_{11}				ref
						CH_4	C_5-C_{11}	C_5-C_{11} yield	lin paraffins	iso-paraffins	olefins	octane number	
		°C	bar(g)	%	$\%_C$	$\%_C$	$\%_C$	$\%_C$	$\%_C$	$\%_C$	$\%_C$		
**Co/SBA15**	SBA15	260	10	81.7	2.4	7.1	54.2	43.3	64.9	7.9	27.2	22.2	
									73.1	8.9	18	13.1	107
									89.2	10.8	0	-4.6	
**Co/Al-SBA15**	SBA15	260	10	64.2	0.9	10.7	62.8	40.0	37.9	21.7	40.5	50.1	
									52.2	29.8	18	31.9	107
									63.6	36.4	0	17.3	

Table S17: reported catalytic performance of bifunctional catalysts for the direct conversion of synthesis gas to gasoline by combining Fe-based FT catalysts and zeolites

catalyst	zeolite	temperature	pressure	CO conversion	CO_2 selectivity	hydrocarbon distribution			C_5-C_{11}					ref
						CH_4	C_5-C_{11}	C_5-C_{11} yield	lin paraffins	iso-paraffins	olefins	aromatics	octane number	
		°C	bar(g)	%	$\%_C$	$\%_C$	$\%_C$	$\%_C$	$\%_C$	$\%_C$	$\%_C$	$\%_C$		
CMA/Hol-Z5-N@S1	H-ZSM-5	280	20	57.3	40.6	2.8	23.9	8.1	8.0	15.0	1.7	75.3	110.0	
									21.0	39.4	4.5	35.0	86.9	88
									32.3	60.7	7.0	0	66.9	
FeK/9mmZ	H-ZSM-5	300	20.0	15.1	50.0	11.0	50.7	3.8	13.4	66.9	0	19.6	78.9	
									13.4	48.9	18	19.6	83.4	111
									13.4	0	66.9	19.6	95.7	
FeK/13mmZ	H-ZSM-5	300	20.0	21.4	50.0	10	52.8	5.7	15.8	57.5	0	26.7	82.0	
									15.8	39.5	18	26.7	86.4	111
									15.8	0	57.5	26.7	96.3	
FeK/17mmZ	H-ZSM-5	300	20.0	20.8	50.0	9	55.1	5.7	16.9	48.8	0	34.3	86.3	
									16.9	30.8	18	34.3	90.7	111
									16.9	0	48.8	34.3	98.3	
Fe-Z-30-5	H-ZSM-5	300	20.0	25.6	*45*	27.3	12.9	1.8	3.3	26.3	3.3	67	96.4	
					estimation				6.6	51.9	6.5	35	67.9	112
									10.1	79.8	10.1	0	36.7	
Fe-Z-50-5	H-ZSM-5	300	20.0	30.9	*45*	28	10.2	1.7	2.9	22.8	3.5	70.8	99.1	
					estimation				6.4	50.8	7.8	35	66.6	112
									9.9	78.2	11.9	0	34.9	
Fe-Z-80-5	H-ZSM-5	300	20.0	69.4	*45*	23.8	32.0	12.2	4.1	30.3	7.8	57.9	90.5	
					estimation				6.3	46.7	12	35	72.0	112
									9.6	71.9	18.5	0	43.9	
Fe-Z-80-10	H-ZSM-5	300	20.0	35	*45*	21.3	36.2	7.0	3.4	26.1	13.4	57	90.7	
					estimation				5.1	39.5	20.3	35	73.1	112
									7.9	60.8	31.3	0	45.0	
Fe-Z-80-15	H-ZSM-5	300	20.0	56.3	*45*	18.7	43.1	13.3	3	23.7	19.6	53.7	89.8	
					estimation				4.3	33.2	27.5	35	75.3	112
									6.6	51.1	42.3	0	48.3	
Fe-Z-100-5	H-ZSM-5	300	20.0	65.3	*45*	25	24.9	8.9	4.5	33.2	18.9	43.4	82.5	
					estimation				5.2	38.1	21.7	35	76.2	112
									7.9	58.6	33.4	0	50.4	
Fe-Z-300-5	H-ZSM-5	300	20.0	73.3	*45*	27.7	15.6	6.3	3.7	27.8	34.5	34	79.2	
					estimation				3.7	44.3	18	34	74.5	112
									3.7	62.3	0	34	69.4	
Fe/SiO2-M	H-ZSM-5	280	10	60	29.9	7	49.3	20.7	14.5	26.2	59.3	0	88.3	
									29.1	52.9	18	0	64.5	113
									35.5	64.5	0	0	54.2	
Fe/SiO2-S-Z	H-ZSM-5	280	10	54.8	33.8	14.9	51.2	18.6	21.3	48	30.7	0	73.8	
									25.2	56.8	18	0	68.4	113
									30.8	69.2	0	0	60.8	
FeNa@Si-c+HZSM-5	H-ZSM-5	260	20	49.8	14.3	7	62.5	26.7	18.3	46.3	10.8	24.6	68.2	114

Table S18: reported catalytic performance of bifunctional OX-ZEO catalysts for the direct conversion of synthesis gas to gasoline.

catalyst	zeolite	temperature	pressure	CO conversion	CO_2 selectivity	hydrocarbon distribution			C_5-C_{11}					ref
						CH_4	C_5-C_{11}	C_5-C_{11} yield	lin paraffins	iso-paraffins	olefins	aromatics	octane number	
		°C	bar(g)	%	$\%_C$	$\%_C$	$\%_C$	$\%_C$	$\%_C$	$\%_C$	$\%_C$	$\%_C$		
Zn2Mn1Ox/SAPO-11 = 2/1	SAPO-11	360	40	20.3	50	2.3	76.7	7.8	3.6	52.3	27.8	16.3	89.4	115
ZnAl2O4/SAPO-11	SAPO-11	350	30	36	44	2.4	70.0	14.1	5.5	77.2	17.3	0	73.1	43
ZnAl2O4/SAPO-31	SAPO-31	350	30	22	40	1.3	66.8	8.8	5	78.1	16.9	0	72.7	43

Table S19: reported catalytic performance of bifunctional catalysts for the direct conversion of synthesis gas to gasoline operated in dual bed mode with dedicated temperatures.

catalyst	zeolite	temperature	pressure	CO conversion	CO_2 selectivity	hydrocarbon distribution			C_5-C_{11}					ref
						CH_4	C_5-C_{11}	C_5-C_{11} yield	lin paraffins	iso-paraffins	olefins	aromatics	octane number	
		°C	bar(g)	%	$\%_C$	$\%_C$	$\%_C$	$\%_C$	$\%_C$	$\%_C$	$\%_C$	$\%_C$		
**CZA + Al2O3[1] nano-H-ZSM-5[2]**	nano-H-ZSM-5	260[1]/320[2]	30	88	32	3	77.8	46.6	2.7	51.1	2.4	43.8	**100.3**	116
									3.1	59.1	2.7	35	**96.4**	
									4.8	91	4.2	0	**80.8**	
**CMA||Z-300**	H-ZSM-5	270[1]/320[2]	10	38	37.5	2.1	69.5	16.5	4.2	21.2	3.1	71.5	**108.1**	83
									9.7	48.3	7	35	**87.5**	
									14.9	74.3	10.8	0	**67.6**	

[1]: upstream bed, [2]: downstream bed

Table S20: combined reported catalytic performance of catalysts for the conversion of synthesis gas to gasoline combining methanol synthesis and MTG in individual processes.

catalyst	conversion %	CO_2 selectivity $\%_C$	methanol selectivity $\%_C$	gasoline selectivity from methanol $\%_C$	gasoline selectivity from synthesis gas $\%_C$	yield $\%_C$	ref
**MeOH**							
Cu/ZnO/Al2O3	8.6	0	97.7			8.4	30
2Cu_MCF 10.7	10.7	0	97			10.4	31
Cu/ZnO/Al2O3	29.9	0	99.6			29.8	30
Cu/ZnO/Al2O3	34.4	0	99.8			34.3	30
Cu/ZnO/Al2O3	40.3	0	98.7			39.8	30
Cu/ZnO/Al2O3	47	0	98.9			46.5	30
**MTG**							
CUO/NH4-ZSM-5(%3)	99.6	0		100		99.6	117
CUO/NH4-ZSM-5(%5)	99.7	0		100		99.7	117
CUO/NH4-ZSM-5(%7)	99.9	0		100		99.9	117
CUO/NH4-ZSM-5(%9)	99	0		100		99	117
Zn/HZ5/0.3AT	100	0		99.4		99.4	117
HZ5/0.3AT	100	0		99.3		99.3	117
HZ5/0.1AT	100	0		99.2		99.2	117
**dual reactor process**							
Cu/ZnO/Al2O3	8.6	0			97	8.4	30,117
2Cu_MCF 10.7	10.7	0			96	10.3	31,117
Cu/ZnO/Al2O3	29.9	0			99	29.6	30,117
Cu/ZnO/Al2O3	34.4	0			99	34.1	30,117
Cu/ZnO/Al2O3	40.3	0			98	39.6	30,117
Cu/ZnO/Al2O3	47	0			98	46.2	30,117

5. References

1. Sun, Y. & Zhao, Z. Implanting Copper−Zinc Nanoparticles into the Matrix of Mesoporous Alumina as a Highly Selective Bifunctional Catalyst for Direct Synthesis of Dimethyl Ether from Syngas. *ChemCatChem* **12**, 1276–1281 (2020).

2. Pinkaew, K. *et al.* A new core-shell-like capsule catalyst with SAPO-46 zeolite shell encapsulated Cr/ZnO for the controlled tandem synthesis of dimethyl ether from syngas. *Fuel* **111**, 727–732 (2013).

3. Lima, S. H., Forrester, A. M. S., Palacio, L. A. & Faro, A. C. Niobia-alumina as methanol dehydration component in mixed catalyst systems for dimethyl ether production from syngas. *Appl. Catal. A Gen.* **488**, 19–27 (2014).

4. Gentzen, M. *et al.* Bifunctional hybrid catalysts derived from Cu/Zn-based nanoparticles for single-step dimethyl ether synthesis. *Catal. Sci. Technol.* **6**, 1054–1063 (2016).

5. Yang, G., Wang, D., Yoneyama, Y., Tan, Y. & Tsubaki, N. Facile synthesis of H-type zeolite shell on a silica substrate for tandem catalysis. *Chem. Commun.* **48**, 1263–1265 (2012).

6. Ahmad, R. *et al.* Zeolite-based bifunctional catalysts for the single step synthesis of dimethyl ether from CO-rich synthesis gas. *Fuel Process. Technol.* **121**, 38–46 (2014).

7. Gentzen, M. *et al.* An intermetallic Pd2Ga nanoparticle catalyst for the single-step conversion of CO-rich synthesis gas to dimethyl ether. *Appl. Catal. A Gen.* **562**, 206–214 (2018).

8. Sai Prasad, P. S., Bae, J. W., Kang, S.-H., Lee, Y.-J. & Jun, K.-W. Single-step synthesis of DME from syngas on Cu–ZnO–Al2O3/zeolite bifunctional catalysts: The superiority of ferrierite over the other zeolites. *Fuel Process. Technol.* **89**, 1281–1286 (2008).

9. Karaman, B. P., Oktar, N., Doğu, G. & Dogu, T. Heteropolyacid Incorporated Bifunctional Core-Shell Catalysts for Dimethyl Ether Synthesis from Carbon Dioxide/Syngas. *Catalysts* **12**, (2022).

10. Kang, S.-H., Bae, J. W., Jun, K.-W. & Potdar, H. S. Dimethyl ether synthesis from syngas over the composite catalysts of Cu–ZnO–Al2O3/Zr-modified zeolites. *Catal. Commun.* **9**, 2035–2039 (2008).

11. Guo, Y. & Zhao, Z. Ethanol as a Binder to Fabricate a Highly-Efficient Capsule-Structured CuO−ZnO−Al2O3@HZSM-5 Catalyst for Direct Production of Dimethyl Ether from Syngas. *ChemCatChem* (2019) doi:10.1002/cctc.201901938.

12. Palomo, J., Rodríguez-Cano, M. Á., Rodríguez-Mirasol, J. & Cordero, T. ZSM-5-decorated CuO/ZnO/ZrO2 fibers as efficient bifunctional catalysts for the direct synthesis of DME from syngas. *Appl. Catal. B Environ.* **270**, 118893 (2020).

13. Jung, J. W. *et al.* Effect of copper surface area and acidic sites to intrinsic catalytic activity for dimethyl ether synthesis from biomass-derived syngas. *Appl. Catal. B Environ.* **126**, 1–8 (2012).

14. Stiefel, M., Ahmad, R., Arnold, U. & Döring, M. Direct synthesis of dimethyl ether from carbon-monoxide-rich synthesis gas: Influence of dehydration catalysts and operating conditions. *Fuel Process. Technol.* **92**, 1466–1474 (2011).

15. Flores, J. H., Peixoto, D. P. B., Appel, L. G., de Avillez, R. R. & Silva, M. I. P. da. The influence of different methanol synthesis catalysts on direct synthesis of DME from syngas. *Catal. Today* **172**, 218–225 (2011).

16. Li, Z., Li, J., Yang, C. & Wu, J. Enhanced catalytic performance for direct synthesis of dimethyl ether from syngas over a La2O3 modified Cu-ZrO2/γ-Al2O3 hybrid catalyst. *J. Nat. Gas Chem.* **21**, 360–365 (2012).

17. Gentzen, M. *et al.* Bifunctional catalysts based on colloidal Cu/Zn nanoparticles for the direct conversion of synthesis gas to dimethyl ether and hydrocarbons. *Appl. Catal. A Gen.* **557**, 99–107 (2018).

18. Lee, Y. J. *et al.* Single-step synthesis of dimethyl ether from syngas on Al2O3-modified CuO–ZnO–Al2O3/ferrierite catalysts: Effects of Al2O3 content. *Catal. Today* **228**, 175–182 (2014).

19. Mejía, C. H., Verbart, D. M. A. & de Jong, K. P. Niobium-based solid acids in combination with a methanol synthesis catalyst for the direct production of dimethyl ether from synthesis gas. *Catal. Today* **369**, 77–87 (2021).

20. Phienluphon, R. *et al.* Designing core (Cu/ZnO/Al2O3)–shell (SAPO-11) zeolite capsule catalyst with a facile physical way for dimethyl ether direct synthesis from syngas. *Chem. Eng. J.* **270**, 605–611 (2015).

21. Guo, X. *et al.* One-step synthesis of dimethyl ether from biomass-derived syngas on CuO-ZnO-Al2O3/HZSM-5 hybrid catalyst: Combination method, synergistic effect, water-gas shift reaction and catalytic performance. *Catal. Today* **407**, 125–134 (2023).

22. Baek, S.-C. *et al.* Effect of Copper Precursors to the Activity for Dimethyl Ether Synthesis from Syngas over Cu–ZnO/γ-Al2O3 Bifunctional Catalysts. *Energy & Fuels* **25**, 2438–2443 (2011).

23. Mao, D. *et al.* Highly effective hybrid catalyst for the direct synthesis of dimethyl ether from syngas with magnesium oxide-modified HZSM-5 as a dehydration component. *J. Catal.* **230**, 140–149 (2005).

24. Liuzzi, D. *et al.* Increasing dimethyl ether production from biomass-derived syngas by in situ steam adsorption. *Sustain. Energy Fuels* (2020) doi:10.1039/D0SE01172J.

25. Guffanti, S., Visconti, C. G. & Groppi, G. Model Analysis of the Role of Kinetics, Adsorption Capacity, and Heat and Mass Transfer Effects in Sorption Enhanced Dimethyl Ether Synthesis. *Ind. Eng. Chem. Res.* **60**, 6767–6783 (2021).

26. Boon, J. & Berkel, F. P. F. Van. Separation Enhanced Dimethyl Ether Synthesis. *Fifth Int. Conf. 2017 Tailor Made Fuels from Biomass* (2017).

27. van Kampen, J., Boon, J., Vente, J. & van Sint Annaland, M. Sorption enhanced dimethyl ether synthesis under industrially relevant conditions: experimental validation of pressure swing regeneration. *React. Chem. Eng.* **6**, 244–257 (2021).

28. van Kampen, J., Boon, J., Vente, J. & van Sint Annaland, M. Sorption enhanced dimethyl ether synthesis for high efficiency carbon conversion: Modelling and cycle design. *J. CO2 Util.* **37**, 295–308 (2020).

29. Guffanti, S., Visconti, C. G., van Kampen, J., Boon, J. & Groppi, G. Reactor modelling and design for sorption enhanced dimethyl ether synthesis. *Chem. Eng. J.* **404**, 126573 (2021).

30. Reubroycharoen, P. *et al.* Continuous low-temperature methanol synthesis from syngas using alcohol promoters. *Energy and Fuels* **17**, 817–821 (2003).

31. Pompe, C. E. *et al.* Stability of mesocellular foam supported copper catalysts for methanol synthesis. *Catal. Today* **334**, 79–89 (2019).

32. Sabour, B., Peyrovi, M. H., Hamoule, T. & Rashidzadeh, M. Catalytic dehydration of methanol to dimethyl ether (DME) over Al-HMS catalysts. *J. Ind. Eng. Chem.* **20**, 222–227 (2014).

33. Liu, X. *et al.* Design of efficient bifunctional catalysts for direct conversion of syngas into lower olefins: Via methanol/dimethyl ether intermediates. *Chem. Sci.* **9**, 4708–4718 (2018).

34. Su, J. *et al.* High Conversion of Syngas to Ethene and Propene on Bifunctional Catalysts via the Tailoring of SAPO Zeolite Structure. *Cell Reports Phys. Sci.* **2**, 100290 (2021).

35. Yang, G., Meng, F., Zhang, P., Yang, L. & Li, Z. Effects of preparation method and precipitant on Mn–Ga oxide in combination with SAPO-34 for syngas conversion into light olefins. *New J. Chem.* **45**, 7967–7976 (2021).

36. Jiao, F. *et al.* Selective conversion of syngas to light olefins. *Science (80-.).* **351**, 1065–1068 (2016).

37. Du, C. *et al.* One-step conversion of syngas to light olefins over bifunctional metal-zeolite catalyst. *Chinese J. Chem. Eng.* **36**, 101–110 (2021).

38. Ren, L. *et al.* Syngas to light olefins over ZnAlOx and high-silica CHA prepared by boron-assisted hydrothermal synthesis. *Fuel* **307**, 121916 (2022).

39. Meng, F. *et al.* Unraveling the role of GaZrOx structure and oxygen vacancy in bifunctional catalyst for highly active and selective conversion of syngas into light olefins. *Chem. Eng. J.* **467**, 143500 (2023).

40. Huang, Y. *et al.* Direct Conversion of Syngas to Light Olefins over a ZnCrOx + H-SSZ-13 Bifunctional Catalyst. *ACS Omega* **6**, 10953–10962 (2021).

41. Su, J. *et al.* Direct Conversion of Syngas into Light Olefins over Zirconium-Doped Indium(III) Oxide and SAPO-34 Bifunctional Catalysts: Design of Oxide Component and Construction of Reaction

Network. *ChemCatChem* **10**, 1536–1541 (2018).

42. Huang, Y. *et al.* Utilization of SAPO-18 or SAPO-35 in the bifunctional catalyst for the direct conversion of syngas to light olefins. *RSC Adv.* **11**, 13876–13884 (2021).

43. Wang, M. *et al.* Effect of zeolite topology on the hydrocarbon distribution over bifunctional ZnAlO/SAPO catalysts in syngas conversion. *Catal. Today* **371**, 85–92 (2021).

44. Meng, F. *et al.* Effect of zeolite topological structure in bifunctional catalyst on direct conversion of syngas to light olefins. *Microporous Mesoporous Mater.* **362**, 112792 (2023).

45. Wang, M. *et al.* Synthesis of hierarchical SAPO-34 to improve the catalytic performance of bifunctional catalysts for syngas-to-olefins reactions. *J. Catal.* **394**, 181–192 (2021).

46. Su, J. *et al.* Syngas to light olefins conversion with high olefin/paraffin ratio using ZnCrO x /AlPO-18 bifunctional catalysts. *Nat. Commun.* **10**, (2019).

47. Jiao, F. *et al.* Shape-Selective Zeolites Promote Ethylene Formation from Syngas via a Ketene Intermediate. *Angew. Chemie - Int. Ed.* **57**, 4692–4696 (2018).

48. Meng, F., Li, B., Zhang, J., Wang, L. & Li, Z. Role of Zn-Al oxide structure and oxygen vacancy in bifunctional catalyst for syngas conversion to light olefins. *Fuel* **346**, 128351 (2023).

49. Li, G. *et al.* Role of SAPO-18 Acidity in Direct Syngas Conversion to Light Olefins. *ACS Catal.* **10**, 12370–12375 (2020).

50. Ding, Y. *et al.* Effects of Proximity-Dependent Metal Migration on Bifunctional Composites Catalyzed Syngas to Olefins. *ACS Catal.* **11**, 9729–9737 (2021).

51. Jiao, F. *et al.* Disentangling the activity-selectivity trade-off in catalytic conversion of syngas to light olefins. *Science (80-.).* **380**, 727–730 (2023).

52. Wang, S. *et al.* Direct Conversion of Syngas into Light Olefins with Low CO2 Emission. *ACS Catal.* **10**, 2046–2059 (2020).

53. Tan, L. *et al.* Design of a core-shell catalyst: an effective strategy for suppressing side reactions in syngas for direct selective conversion to light olefins. *Chem. Sci.* **11**, 4097–4105 (2020).

54. Zhong, L. *et al.* Cobalt carbide nanoprisms for direct production of lower olefins from syngas. *Nature* **538**, 84–87 (2016).

55. Gu, B. *et al.* Effects of the promotion with bismuth and lead on direct synthesis of light olefins from syngas over carbon nanotube supported iron catalysts. *Appl. Catal. B Environ.* **234**, 153–166 (2018).

56. Xie, J. *et al.* Promoted cobalt metal catalysts suitable for the production of lower olefins from natural gas. *Nat. Commun.* **10**, 1–10 (2019).

57. Liu, Z., Jia, G., Zhao, C. & Xing, Y. Efficient Fischer-Tropsch to light olefins over iron-based catalyst with low methane selectivity and high olefin/paraffin ratio. *Fuel* **288**, 119572 (2021).

58. Zhai, P. *et al.* Highly Tunable Selectivity for Syngas-Derived Alkenes over Zinc and Sodium-Modulated Fe5C2Catalyst. *Angew. Chemie - Int. Ed.* **55**, 9902–9907 (2016).

59. Fatih, Y., Burgun, U., Sarioglan, A. & Atakül, H. Effect of sodium incorporation into Fe-Zn catalyst for Fischer- Tropsch synthesis to light olefins. *Mol. Catal.* **535**, 112866 (2023).

60. Torres Galvis, H. M. *et al.* Effect of precursor on the catalytic performance of supported iron catalysts for the Fischer-Tropsch synthesis of lower olefins. *Catal. Today* **215**, 95–102 (2013).

61. Torres Galvis, H. M. *et al.* Supported iron nanoparticles as catalysts for sustainable production of lower olefins. *Science (80-.).* **335**, 835–838 (2012).

62. Liu, X., Lin, T., Liu, P. & Zhong, L. Hydrophobic interfaces regulate iron carbide phases and catalytic performance of FeZnOx nanoparticles for Fischer-Tropsch to olefins. *Appl. Catal. B Environ.* **331**, 122697 (2023).

63. Di, Z., Zhao, T., Feng, X. & Luo, M. A Newly Designed Core-Shell-Like Zeolite Capsule Catalyst for Synthesis of Light Olefins from Syngas via Fischer–Tropsch Synthesis Reaction. *Catal. Letters* **149**,

441–448 (2019).

64. Wu, L. & Hensen, E. J. M. Comparison of mesoporous SSZ-13 and SAPO-34 zeolite catalysts for the methanol-to-olefins reaction. *Catal. Today* **235**, 160–168 (2014).

65. Huang, Z. *et al.* Ceria-Zirconia/Zeolite Bifunctional Catalyst for Highly Selective Conversion of Syngas into Aromatics. *ChemCatChem* **10**, 4519–4524 (2018).

66. Yang, J., Pan, X., Jiao, F., Li, J. & Bao, X. Direct conversion of syngas to aromatics. *Chem. Commun.* **53**, 11146–11149 (2017).

67. Ji, Y. *et al.* Oxygenate-based routes regulate syngas conversion over oxide–zeolite bifunctional catalysts. *Nat. Catal.* **5**, 594–604 (2022).

68. Ma, D. *et al.* The Direct Synthesis of Aromatic Hydrocarbons from Syngas over Bifunctional MgZrOx/HZSM-5 Catalysts. *Catalysts* vol. 13 (2023).

69. Wang, S., Fang, Y., Huang, Z., Xu, H. & Shen, W. The Effects of the Crystalline Phase of Zirconia on C – O Activation and C – C Coupling in Converting Syngas into Aromatics. *Catalysts* **10**, (2020).

70. Fu, Y. *et al.* Insights into the size effect of ZnCr2O4 spinel oxide in composite catalysts for conversion of syngas to aromatics. *Green Energy Environ.* (2021) doi:https://doi.org/10.1016/j.gee.2021.07.003.

71. Yang, X. *et al.* The influence of intimacy on the 'iterative reactions' during OX-ZEO process for aromatic production. *J. Energy Chem.* **35**, 60–65 (2019).

72. Arslan, M. T. *et al.* Highly Selective Conversion of CO2 or CO into Precursors for Kerosene-Based Aviation Fuel via an Aldol–Aromatic Mechanism. *ACS Catal.* **12**, 2023–2033 (2022).

73. Yang, J. *et al.* Enhanced aromatic selectivity by the sheet-like ZSM-5 in syngas conversion. *J. Energy Chem.* **35**, 44–48 (2019).

74. Cheng, K. *et al.* Bifunctional Catalysts for One-Step Conversion of Syngas into Aromatics with Excellent Selectivity and Stability. *Chem* **3**, 334–347 (2017).

75. Ma, Z. *et al.* Catalytic roles of acid property in different morphologies of H-ZSM-5 zeolites for syngas-to-aromatics conversion over ZnCrOx/H-ZSM-5 catalysts. *Microporous Mesoporous Mater.* **349**, 112420 (2023).

76. Liu, J. *et al.* Nano-ZrO2 as hydrogenation phase in bi-functional catalyst for syngas aromatization. *Fuel* **263**, 116803 (2020).

77. Zhou, W. *et al.* Selective Conversion of Syngas to Aromatics over a Mo−ZrO 2 /H-ZSM-5 Bifunctional Catalyst. *ChemCatChem* **11**, 1681–1688 (2019).

78. Tian, G. *et al.* Accelerating syngas-to-aromatic conversion via spontaneously monodispersed Fe in ZnCr2O4 spinel. *Nat. Commun.* **13**, 5567 (2022).

79. Zhang, P., Tan, L., Yang, G. & Tsubaki, N. One-pass selective conversion of syngas to para-xylene. *Chem. Sci.* **8**, 7941–7946 (2017).

80. Zhou, W. *et al.* Direct conversion of syngas into aromatics over a bifunctional catalyst: Inhibiting net CO2release. *Chem. Commun.* **56**, 5239–5242 (2020).

81. Wang, Y. *et al.* Boosting the synthesis of value-added aromatics directly from syngas via a Cr2O3 and Ga doped zeolite capsule catalyst. *Chem. Sci.* **12**, 7786–7792 (2021).

82. Xu, Y., Liu, D. & Liu, X. Conversion of syngas toward aromatics over hybrid Fe-based Fischer-Tropsch catalysts and HZSM-5 zeolites. *Appl. Catal. A Gen.* **552**, 168–183 (2018).

83. Tao Sun, Tiejun Lin, Yunlei An, Kun Gong, Liangshu Zhong, and Y. S. Syngas Conversion to Aromatics over Co 2 C- based Catalyst and HZSM-5 via Tandem System. *Ind. Eng. Chem. Res.* **59**, 4419–4427 (2020).

84. Wang, T. *et al.* Sodium-Mediated Bimetallic Fe–Ni Catalyst Boosts Stable and Selective Production of Light Aromatics over HZSM-5 Zeolite. *ACS Catal.* **11**, 3553–3574 (2021).

85. Xu, Y. *et al.* Yolk@Shell FeMn@Hollow HZSM-5 Nanoreactor for Directly Converting Syngas to

Aromatics. *ACS Catal.* **11**, 4476–4485 (2021).

86. Xu, Y. *et al.* Selective Conversion of Syngas to Aromatics over Fe3O4@MnO2 and Hollow HZSM-5 Bifunctional Catalysts. *ACS Catal.* **9**, 5147–5156 (2019).

87. Xu, Y. *et al.* Synthesis of aromatics from syngas over FeMnK/SiO2 and HZSM-5 tandem catalysts. *Mol. Catal.* **454**, 104–113 (2018).

88. Wang, H. *et al.* Bifunctional catalysts with versatile zeolites enable unprecedented para-xylene productivity for syngas conversion under mild conditions. *Chem Catal.* **2**, 779–796 (2022).

89. Nawaz, M. A. *et al.* Tailoring the synergistic dual-decoration of (Cu–Co) transition metal auxiliaries in Fe-oxide/zeolite composite catalyst for the direct conversion of syngas to aromatics. *Catal. Sci. Technol.* **11**, 7992–8006 (2021).

90. Zhao, B. *et al.* Direct Transformation of Syngas to Aromatics over Na-Zn-Fe5C2 and Hierarchical HZSM-5 Tandem Catalysts. *Chem* **3**, 323–333 (2017).

91. Kang, S. C. *et al.* Enhancing selectivity of aromatics in direct conversion of syngas over K/FeMn and HZSM-5 bifunctional catalysts. *Mol. Catal.* **533**, 112790 (2022).

92. Wen, C. *et al.* Insight into the direct conversion of syngas toward aromatics over the Cu promoter Fe-zeolite tandem catalyst. *Fuel* **331**, 125855 (2023).

93. Jia, Y. *et al.* Hierarchical ZSM-5 zeolite synthesized via dry gel conversion-steam assisted crystallization process and its application in aromatization of methanol. *Powder Technol.* **328**, 415–429 (2018).

94. Gao, P. *et al.* A Mechanistic Study of Methanol-to-Aromatics Reaction over Ga-Modified ZSM-5 Zeolites: Understanding the Dehydrogenation Process. *ACS Catal.* **8**, 9809–9820 (2018).

95. Kim, S., Kim, Y. T., Hwang, A., Jun, K. W. & Kwak, G. Coke-Tolerant Gadolinium-Promoted HZSM-5 Catalyst for Methanol Conversion into Hydrocarbons. *ChemCatChem* **9**, 1569–1573 (2017).

96. Niu, X. *et al.* Influence of crystal size on the catalytic performance of H-ZSM-5 and Zn/H-ZSM-5 in the conversion of methanol to aromatics. *Fuel Process. Technol.* **157**, 99–107 (2017).

97. Zhang, J. *et al.* Solvent-Free Synthesis of Core-Shell Zn/ZSM-5@Silicalite-1 Catalyst for Selective Conversion of Methanol to BTX Aromatics. *Ind. Eng. Chem. Res.* **58**, (2019).

98. Cai, L., Tripathi, R., Broda, R. & Pitsch, H. A property database of fuel compounds with emphasis on spark-ignition engine applications. *Appl. Energy Combust. Sci.* **5**, 100018 (2021).

99. Perdih, A. & Perdih, F. Chemical interpretation of octane number. *Acta Chim. Slov.* **53**, 306–315 (2006).

100. Eglof, Gusftav And Arsdell, P. M. Van. Octane rating relationships of aliphatic, alicyclic, mononuclear aromatic. *Inst. Pet.* **27**, 121–138 (1941).

101. Lu, P. *et al.* Sputtered nano-cobalt on H-USY zeolite for selectively converting syngas to gasoline. *J. Energy Chem.* **24**, 637–641 (2015).

102. Li, J. *et al.* Integrated tuneable synthesis of liquid fuels via Fischer–Tropsch technology. *Nat. Catal.* **1**, 787–793 (2018).

103. Xing, C. *et al.* Hierarchical zeolite y supported cobalt bifunctional catalyst for facilely tuning the product distribution of Fischer-Tropsch synthesis. *Fuel* **148**, 48–57 (2015).

104. Subramanian, V. *et al.* The Role of Steric Effects and Acidity in the Direct Synthesis of iso-Paraffins from Syngas on Cobalt Zeolite Catalysts. *ChemCatChem* **8**, 380–389 (2016).

105. Sartipi, S., Van Dijk, J. E., Gascon, J. & Kapteijn, F. Toward bifunctional catalysts for the direct conversion of syngas to gasoline range hydrocarbons: H-ZSM-5 coated Co versus H-ZSM-5 supported Co. *Appl. Catal. A Gen.* **456**, 11–22 (2013).

106. Zhu, C. & Bollas, G. M. Gasoline selective Fischer-Tropsch synthesis in structured bifunctional catalysts. *Appl. Catal. B Environ.* **235**, 92–102 (2018).

107. Xing, C. *et al.* Completed encapsulation of cobalt particles in mesoporous H-ZSM-5 zeolite catalyst for direct synthesis of middle isoparaffin from syngas. *Catal. Commun.* **55**, 53–56 (2014).

108. Chen, Y. *et al.* Nano-ZSM-5 decorated cobalt based catalysts for Fischer-Tropsch synthesis to enhance the gasoline range products selectivity. *J. Taiwan Inst. Chem. Eng.* **116**, 153–159 (2020).

109. Wang, H. *et al.* The effect of the particle size on Fischer–Tropsch synthesis for ZSM-5 zeolite supported cobalt-based catalysts. *Chem. Commun.* **57**, 13522–13525 (2021).

110. Yakovenko, R. E. *et al.* Selective Synthesis of a Gasoline Fraction from CO and H2 on a Co-SiO2/ZSM-5/Al2O3 Catalyst. *Catalysts* vol. 13 (2023).

111. Weber, J. L. *et al.* Conversion of synthesis gas to aromatics at medium temperature with a fischer tropsch and ZSM-5 dual catalyst bed. *Catal. Today* **369**, 175–183 (2021).

112. Wen, C. *et al.* Effect of hierarchical ZSM-5 zeolite support on direct transformation from syngas to aromatics over the iron-based catalyst. *Fuel* **244**, 492–498 (2019).

113. Jin, Y. *et al.* Development of dual-membrane coated Fe/SiO2 catalyst for efficient synthesis of isoparaffins directly from syngas. *J. Memb. Sci.* **475**, 22–29 (2015).

114. Xu, Y. *et al.* Insights into the Diffusion Behaviors of Water over Hydrophilic/Hydrophobic Catalysts During the Conversion of Syngas to High-Quality Gasoline. *Angew. Chemie* **135**, (2023).

115. Li, N. *et al.* High-Quality Gasoline Directly from Syngas by Dual Metal Oxide–Zeolite (OX-ZEO) Catalysis. *Angew. Chemie* **131**, 7478–7482 (2019).

116. Ni, Y. *et al.* Realizing high conversion of syngas to gasoline-range liquid hydrocarbons on a dual-bed-mode catalyst. *Chem Catal.* https://doi.org/10.1016/j.checat.2021.02.003 (2021) doi:10.1016/j.checat.2021.02.003.

117. Kianfar, E., Hajimirzaee, S., mousavian, S. & Mehr, A. S. Zeolite-based catalysts for methanol to gasoline process: A review. *Microchem. J.* **156**, 104822 (2020).
